# Triflyl [^18^F]Fluoride as a Solution for Base‐Sensitive Late‐Stage Nucleophilic Aromatic ^18^F‐Fluorination Reactions

**DOI:** 10.1002/chem.202403127

**Published:** 2024-11-26

**Authors:** Lizeth Y. F. Haveman, Anna M. T. de Kruijff, Sjoerd P. P. van Eeden, Albert D. Windhorst, Danielle J. Vugts

**Affiliations:** ^1^ Deparment of Radiology & Nuclear Medicine Amsterdam UMC, location Vrije Universiteit Amsterdam, De Boelelaan 1117 1081 HV Amsterdam The Netherlands

**Keywords:** Triflyl [^18^F]fluoride, Fluorine-18, Radiochemistry, Positron emission tomography

## Abstract

Fluorine‐18 is the predominant radionuclide used to label Positron Emission Tomography (PET) tracers. One outstanding challenge in nucleophilic aromatic radiofluorination reactions is the sensitivity of precursors and catalysts for basic reaction conditions, which are necessary for the work‐up of [^18^F]fluoride, resulting in limited reproducibility. Triflyl [^18^F]fluoride is a new [^18^F]fluoride source that allows freedom in choice of type and amounts of base and cryptand. The aim of the current work is to explore the scope and limitations of triflyl [^18^F]fluoride in the late‐stage nucleophilic aromatic ^18^F‐fluorination of various functionalized precursors, exploring reduced amounts of base and cryptand. The assessment allowed for the application of this new nucleophilic [^18^F]fluoride reagent to the successful radiosynthesis of boron, stannane, hypervalent iodonium ylide and phenol substrates bearing electron‐deficient, ‐neutral and ‐rich functional groups as well as the clinically relevant PET tracers [^18^F]FPEB, [^18^F]*m*FBG and [^18^F]SynVesT‐1.

## Graphical abstract

Here, we present the late‐stage nucleophilic 18F‐fluorination of (hetero)aryls under low‐base conditions using triflyl [^18^F]fluoride. This method avoids the application of base and cryptand and enabled the efficient radiolabeling of a broad scope of (hetero)arenes and the successful production of clinical doses of [^18^F]mFBG, [18F]SynVesT‐1 and [^18^F]FPEB in improved radiochemical yields (RCY) and/or shorter preparation times.

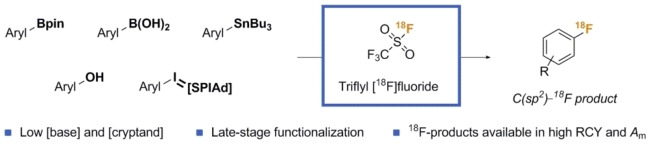

## Introduction

Positron emission tomography (PET) has proven to be an essential imaging technology in modern‐day medicine.^[1]^ PET enables the non‐invasive visualization of physiochemical and pathological processes *in vivo* at a molecular level. The successful application of PET in patient care, clinical research and drug development is highly dependent on the availability of biologically active molecules labeled with a positron‐emitting radionuclide, dedicated to selectively address targets of high relevance in the disease of interest. The excellent nuclear decay properties of fluorine‐18 with respect to half‐life (110 min) and decay energy (*E*β^+^_max_=634 keV) makes this radionuclide the most popular isotope used in PET.^[1]^ Moreover, the abundance of fluorine substituents in marketed drugs and biologically active compounds is increasing, thereby expanding the scope for tracer development.

For long, nucleophilic aromatic ^18^F‐fluorinations were limited to substitution of a nitro or tri‐alkyl ammonium group positioned *ortho* or *para* to an electron‐withdrawing group. Since the establishment of modern ‘late‐stage’ nucleophilic aromatic radiofluorination reactions, especially Cu‐mediated ^18^F‐fluorination reactions, it has become possible to prepare non‐activated and *meta*‐substituted aryl‐^18^F motifs as well as PET imaging agents in routine preclinical and clinical productions.^[2]^ Despite the many new achievements, one outstanding challenge is the reproducibility of these reactions.^[1]^ The work‐up procedure for [^18^F]fluoride requires high amounts of base and phase transfer catalyst, which are often incompatible with the subsequent ^18^F‐fluorination reaction. With careful control of the reaction conditions, these problems can be overcome, however such a delicate balance hampers the reproducibility of the reaction and with that the practical use.

To solve this difficulty, either the work‐up procedure of [^18^F]fluoride has to change or a new [^18^F]fluoride source that avoids strong basic conditions has to be developed. To this end, our lab published a promising approach towards [^18^F]fluoride work‐up by employing triflyl [^18^F]fluoride as a versatile [^18^F]fluoride source, which can be obtained in high yield (>90 %), high purity (>98 %) and high molar activity within a few minutes after [^18^F]fluoride production.^[3]^ The release of [^18^F]fluoridefrom triflyl [^18^F]fluoride can be achieved with a 10–100 fold reduced concentration of base and phase transfer catalyst compared to commonly used in conventional nucleophilic radiofluorinations. Furthermore, the exact conditions of conversion of triflyl [^18^F]fluoride to [^18^F]fluoride can be tailored to the subsequent labeling conditions.

Although triflyl [^18^F]fluoride is finding its way into the radiochemistry field as a practical [^18^F]fluoride source, the flexible radiolabeling conditions have only found application in the radiosynthesis of a limited number of model compounds and useful PET tracers.[^3^, ^4^, ^5^, ^6^] Here, we assessed the reactivity of the [^18^F]fluoride generated from triflyl [^18^F]fluoride in late‐stage nucleophilic aromatic ^18^F‐fluorination reactions in order to implement triflyl [^18^F]fluoride in the ^18^F‐radiochemistry toolbox. This study addresses the influence of the reaction parameters on the radiofluorination procedures of several substrate classes as well as steric and electronic factors of the substrates to define its exploratory power. Also, clinically relevant tracers have been synthesized to prove the applicability of this method. Altogether, it is believed that this assessment facilitates the use of triflyl [^18^F]fluoride in the radiofluorination of structurally different compounds and ultimately complex pharmaceutically relevant molecules.

## Results and Discussion

**Figure 1:**
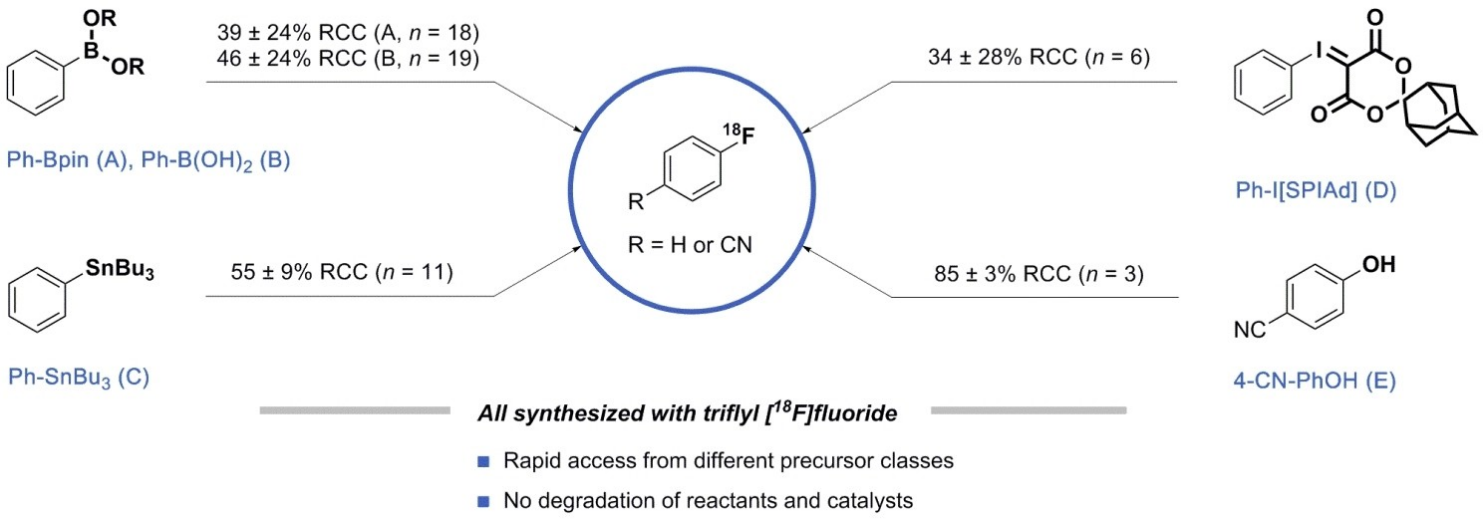
The ^18^F‐fluorination reactions A–E were selected as examples for the sensitivity assessment of triflyl [^18^F]fluoride. The following conditions are the standard conditions used as reference point to examine the influence of key parameters on the RCC as described in the ‘Design of experiments’: A) Ph‐Bpin (60 μmol), Cu(OTf)_2_(py)_4_ (5 μmol), DMF (400 μL), 110 °C, 20 min; B) Ph−B(OH)_2_ (4 μmol), Cu(OTf)_2_ (20 μmol), pyridine (500 μmol), DMF (1.0 mL), 110 °C, 20 min; C) Ph‐SnBu_3_ (30 μmol), Cu(OTf)_2_(py)_4_ (30 μmol), DMA (1.0 mL), 110 °C, 15 min; D) Ph−I[SPIAd] (4 μmol), TEAB (10 μmol), DMF (400 μL), 120 °C, 10 min; E) 4‐cyanophenol (10 μmol), imidazolium chloride (10 μmol), Ag_2_CO_3_ (5 μmol), *n*‐butanol/EtOH (600 μL), 130 °C, 20 min. RCC determined by radioHPLC.

**Figure 2:**
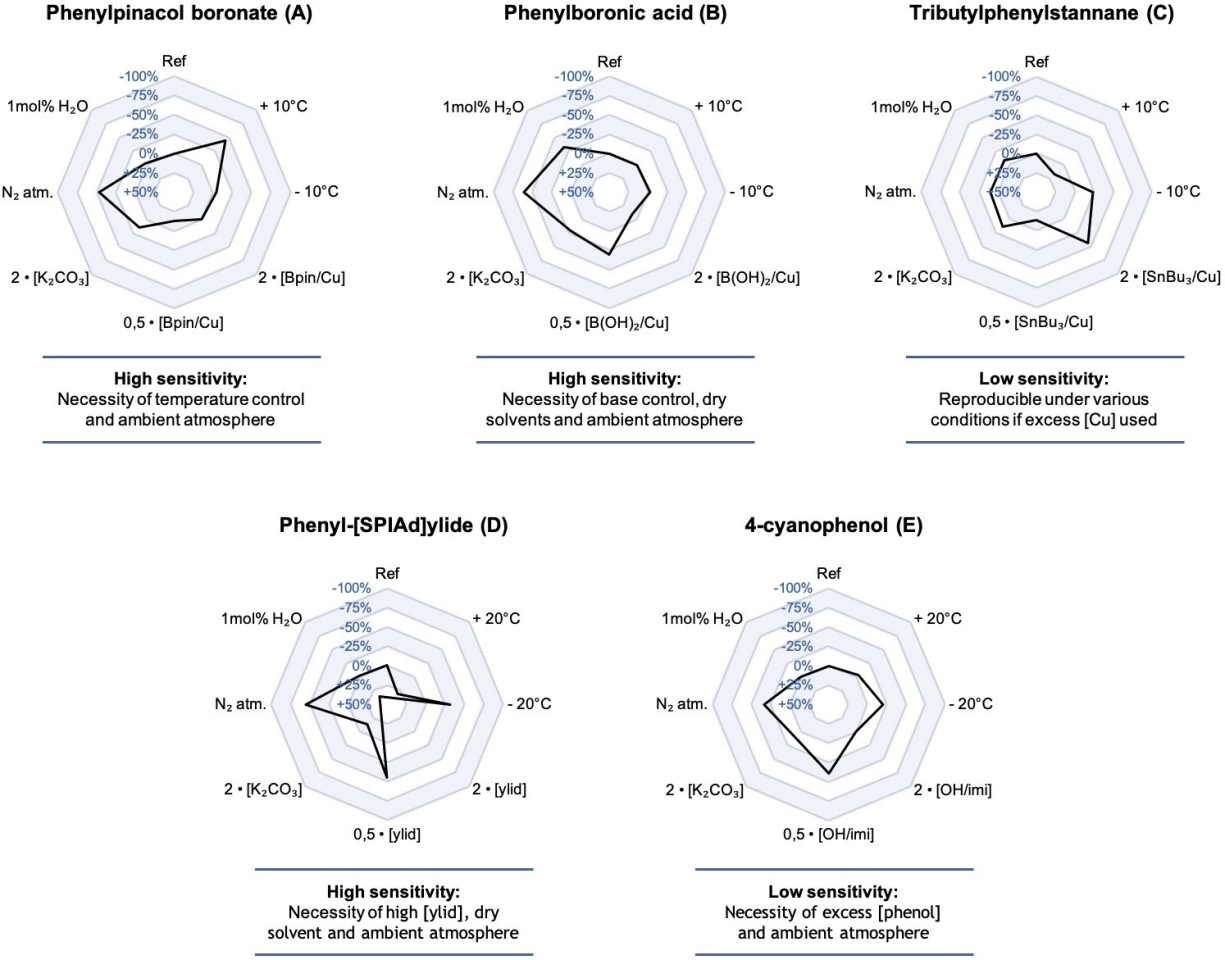
Sensitivity assessment of the five investigated reactions **A–E** illustrated as radar diagrams. An almost round shape around the “0 % deviation line” indicates low sensitivity, any line deflecting from that to the outer zones refers to high sensitivity. See Supporting Information for experimental details.

To exemplify the value of triflyl [^18^F]fluoride in the current field of ^18^F‐fluorination methodologies, the Cu‐mediated radiofluorination of phenylpinacol boronate (Ph‐Bpin; **A**), phenylboronic acid (Ph−B(OH)_2_; **B**) and tributylphenylstannane (Ph‐SnBu_3_; **C**), the ^18^F‐fluorination of the aromatic hypervalent iodonium(III) ylide (Ph−I[SPIAd]; **D**) and the ^18^F‐deoxyfluorination of 4‐cyanophenol (4‐CN‐PhOH; **E**) were selected because of their versatile reactivity profiles (Figure 1).^[1]^ The design of the experiments was based on the variation of one parameter per experiment relative to reaction conditions selected from the hallmark papers for each substrate class.[^7^, ^8^, ^9^, ^10^, ^11^] The influence of each parameter is depicted as a black line in a radar diagram as introduced by Pitzer *et al*. (Figure 2).^[12]^ The following parameters were varied to demonstrate the sensitivity of the reaction using triflyl [^18^F]fluoride as [^18^ F]fluoride source: temperature, solvent, amount of precursor and additive, reaction atmosphere, water and base content.[^13^, ^14^]

### ^18^F‐Fluorination of Phenylpinacol Boronate (A)

The late‐stage Cu‐mediated radiofluorination of phenylboronic acid pinacol ester (Ph‐Bpin) showed a strong dependence on the temperature (Figure 2A). A slight increase of 10 °C already led to almost a 50 % reduction in RCC compared to the reference reaction due to degradation products (22±13 % vs 39±24 %). A pivotal role for oxygen in facilitating the re‐oxidation of the Cu catalyst was observed, since using N_2_ to purge the reaction vial resulted in a drop in RCC of around 50 %. A purge with 5 mL of dried purified air, however, did not further enhance the radiochemical conversion indicating that an ambient atmosphere is sufficient (see Table S2). Predictably, a change in the amount of catalyst and precursor affected the radiochemical conversion, although the effect was only modest. A lower Ph‐Bpin/Cu ratio was found to be advantageous with equimolar amounts of Ph‐Bpin (20 μmol) and Cu(OTf)_2_(py)_4_ (20 μmol) resulting in a still moderate, but now reliable RCC of 43±2 % (see Table S1). Noteworthy, reliable conversions were obtained with Ph‐Bpin/Cu ratios of 2/1 to 1/1.5 in contrast to the low reproducibility of the current methodologies. In addition, an excess of Cu has been reported to be desirable for the efficient ^18^F‐labeling of electron‐deficient arenes,^[15]^ but is not always possible due to the strong basic conditions resulting from the [^18^F]fluoride work‐up. Accordingly, increasing concentration of K_2_CO_3_/K_222_ were detrimental for the ^18^F‐labeling due to decomposition of the Cu catalyst (see Table S2). Finally, re‐evaluation of the reaction solvent and water content did not lead to significant changes (see Tables S1 and S2).

### ^18^F‐Fluorination of Phenylboronic Acid (B)

The assessment of procedure **B** revealed a poor water tolerance and high sensitivity to strong bases (Figure 2B). For example, ca. 25 % reduction in RCC was already observed in aqueous DMF (1 mol % H_2_O) or in the presence of only 1.0/2.0 μmol of K_2_CO_3_/K_222_ (35±3 % and 34±8 %, respectively, vs. 46±24 % RCC) (see Table S4). This effect is presumably caused by the faster decomposition of Cu(OTf)_2_ in the presence of water or base compared to Cu(OTf)_2_(py)_4_. As the reaction also proceeds through a Cu‐catalytic oxidation/reduction cycle, a strong sensitivity for the oxygen level was expected and indeed found. In addition, a dependence on the loading of Ph−B(OH)_2_ and Cu(OTf)_2_ was found. Noteworthy, a 10–50 % increase in RCC was obtained when only 6.0 μmol of Cu(OTf)_2_ was used instead of 12 μmol independent of the concentration of Ph−B(OH)_2_ investigated (see Table S3). However, at least equimolar amounts of Ph−B(OH)_2_ and Cu(OTf)_2_, but preferably a slight excess of Cu, are required in order to obtain reproducible yields. Moreover, a screen of different solvents showed a strong preference for DMF over DMA and *N*‐methyl‐2‐pyrrolidone (NMP) (see Table S3). Both the solvent preference and dependence on the amount of Cu instead of substrate/Cu ratio differs from the results obtained for the ^18^F‐fluorination of Ph‐Bpin. This suggests that a deeper understanding of the crucial conditions for Cu‐mediated radiofluorination reactions is still needed. Finally, a 10 °C variation in the temperature caused no effects making this procedure amenable to clinical productions.

### ^18^F‐Fluorination of Tributylphenylstannane (C)

The Cu‐mediated ^18^F‐fluorination of tributylphenylstannane (Ph‐SnBu_3_) represented a robust reaction compared to the boron precursors (Figure 2C). Parameters such as water concentration and reaction atmosphere seemed not to have a strong influence on the reaction outcome causing only a reduction of ca. 10 % in RCC. This observation is in accordance with the significantly faster transmetallation step of tin versus boron and is expected to be unperturbed by minor condition changes.^[9]^ Notably, an increase in temperature from 110 °C to 120 °C was optimal resulting in a RCC of 62±7 % (see Table S5). This elevated temperature is still sufficiently mild to work with in the case of more sensitive substrates. As expected, DMA and NMP afforded good conversions (61±6 % and 61±3 %, respectively), whereas in DMF only 17±2 % ^18^ F‐incorporation was observed (see Table S5). Finally, the reaction was strongly dependent on the Ph‐SnBu_3_/Cu ratio. An excess of Cu(OTf)_2_(py)_4_ was required, but Ph‐SnBu_3_/Cu(OTf)_2_(py)_4_ ratios of 1/4 to 2/3 all provided good RCCs (65–70 %, see Table S5). A marked decrease of conversion was first observed at equimolar amounts of substrate and Cu or if the amount of Cu catalyst was less than 30 μmol. Hence, based on the physical chemical nature of the stannane precursor it can be determined whether an increased excess of Cu catalyst is desirable and in which concentrations both substrate and catalyst should be applied. Finally, in accordance with the observed sensitivity of Cu(OTf)_2_(py)_4_ for high base concentrations in the ^18^F‐fluorination of Ph‐Bpin, increased K_2_CO_3_/K_222_ concentrations were detrimental for the ^18^F‐labeling of Ph‐SnBu_3_ as well (see Table S6). This suggests that a practitioner should mainly focus on a low‐base procedure with an ambient atmosphere when using this ^18^F‐fluorodestannylation reaction.

Since the report from Zischler *et al*. on the beneficial effect of alcohols in Cu‐mediated radiofluorinations, improvement in RCYs of several pre‐clinical tracers (e. g. [^18^F]darapladib and [^18^F]TRACK) have been achieved.[^16^, ^17^, ^18^] Amongst the investigational reports, mostly DMA/*n*BuOH mixtures have been used as reaction medium with generally a good tolerance of up to 20–30 % of alcohol. Accordingly, with DMA/*n*BuOH (2 : 1) as solvent mixture the radiolabeling yields exceeded 75 % for stannane substrates as well as for the boron precursors (see Tables S1, S3 and S5).

Altogether, the Cu‐mediated aromatic ^18^F‐fluorination of boron and stannane substrates with triflyl [^18^F]fluoride occurs in the same reaction medium, within the same temperature range, with a wider concentration range than generally described and with the potential to interchange the Cu catalysts (see Tables S2, S4 and S6). Hence, the currently described method has the potential to offer a general method for the radiofluorination of a larger range of boron and stannane substrates than previously performed.

### ^18^F‐Fluorination of Phenyl‐[SPIAd]ylide (D)

The ^18^F‐fluorination of (1r,3r,5r,7r)‐5′‐(phenyl‐l3‐iodanylidene)spiro[adamantane‐2,2′‐[1,3]dioxane]‐4′,6′‐dione (Ph−I[SPIAd]) showed to be very sensitive to several parameters including the reaction atmosphere (Figure 2D). The necessity of oxygen in the reaction mixture was not expected, since an inert atmosphere was reported to have a positive effect on the RCC.^[19]^ Although this observation was explained by preventing oxidation of the triphenylphosphine catalyst used, the reaction in the absence of this catalyst but with a N_2_ purge also showed an increase in conversion. Why we observe such a contradicting behavior is not clear. In addition, the temperature showed only modest effects on the conversion rate. Moderate incorporations were achieved at 120–150 °C (26–34 % RCC), with 140 °C being optimal with respect to reliability (see Table S7). Figure 2D also shows a strong sensitivity to the reagent amounts. A reduction of the mass of Ph−I[SPIAd] from 4.0 to 2.0 μmol caused a significant decrease in RCC from 34±28 % to 11±3 %, whilst an increase to 32 μmol resulted in an almost linear increase in RCC up to 61±14 % (see Table S7). Hence a higher amount of precursor seems preferable and it is on account of the chemist to decide how much starting material is optimal. In contrast, decreasing the amount of TEAB from 40 to 10 μmol did not lead to a change in RCC due to decomposition of the precursor. The use of 10–50 μmol of TEAB has been shown to be used for the radiofluorination of iodonium ylides in past studies as well.^[20]^ Furthermore, the incorporation yields were unaffected by using K_222_ as complexing agent and K_2_CO_3_ as the base under otherwise identical conditions. With 0.5–16.0 μmol of K_2_CO_3_ good conversions up to 49±5 % could be achieved (see Table S8). Finally, a comparison of solvents showed that DMF and DMSO are the preferred reaction solvents, which is in agreement with past reports (see Table S7).^[20]^

### ^18^F‐Fluorination of 4‐Cyanophenol (E)

In 2016, Neumann *et al*. reported the two‐step ^18^F‐deoxyfluorination of phenols with [^18^F]fluoride and we sought to either simplify or optimize this procedure to a one‐step radiofluorination protocol with triflyl [^18^F]fluoride.^[11]^ As such, [^18^F]fluoride was generated from trapped triflyl [^18^F]fluoride in a vial containing Ag_2_CO_3_ and a solution of K_2_CO_3_/K_222_ in MeCN. *p*‐Cyanophenol and 2‐chloro‐1,3‐bis(2,6‐diisopropylphenyl)‐1H‐ imidazolium chloride (imi‐Cl) were then added in DMSO and the reaction mixture was heated at 130 °C for 20 min. Complete conversion of [^18^F]fluoride into 4‐[^18^F]fluorobenzonitrile was observed without the necessity to prepare the uranium complex prior to labeling and avoiding the low elution efficiencies of [^18^F]fluoride from the ion‐exchange cartridge during the [^18^F]fluoride work‐up procedure.^[11]^ Regarding the ^18^F‐deoxyfluorination of *p*‐cyanophenol, an eminently robust reaction was observed (Figure 2E). The only requirement was the presence of equimolar amounts of the precursor or an excess of the precursor compared to imi‐Cl combined with DMSO/MeCN (1 : 1) as reaction solvent. The excess of *p*‐cyanophenol will prevent the *in situ* formation of the monofluoroimidazolium salt analogue of PhenoFluor, which cannot be attacked by the phenolic precursor to yield the active tetrahedral intermediate.^[11]^ In addition, the reaction seemed not dependent on the concentration of reactants as long as the *p*‐cyanophenol was in excess, facilitating the use of very small amounts of starting materials (see Table S9). Notably, the reported solvent mixture 2‐butanone/EtOH (9 : 1) only gave 24±5 % conversion, which is presumably due to the low boiling point of 2‐butanone (see Table S9). Other parameters seemed not to have a strong influence on the reaction outcome. Lowering the reaction temperature afforded slightly lower yields of product, but still >65 % RCC at 110 °C, offering the possibility to use milder conditions if necessary (see Table S9). In addition, no precautions to exclude moisture or air from the ^18^F‐deoxyfluorination reaction were necessary (see Table S10). Altogether, the ^18^F‐deoxyfluorination reaction with triflyl [^18^F]fluoride results in desirable yields under ambient atmosphere and using equal amounts or an excess of phenol precursor compared to imi‐Cl.

### Influence of Functional Groups on Radiofluorination

**Figure 3:**
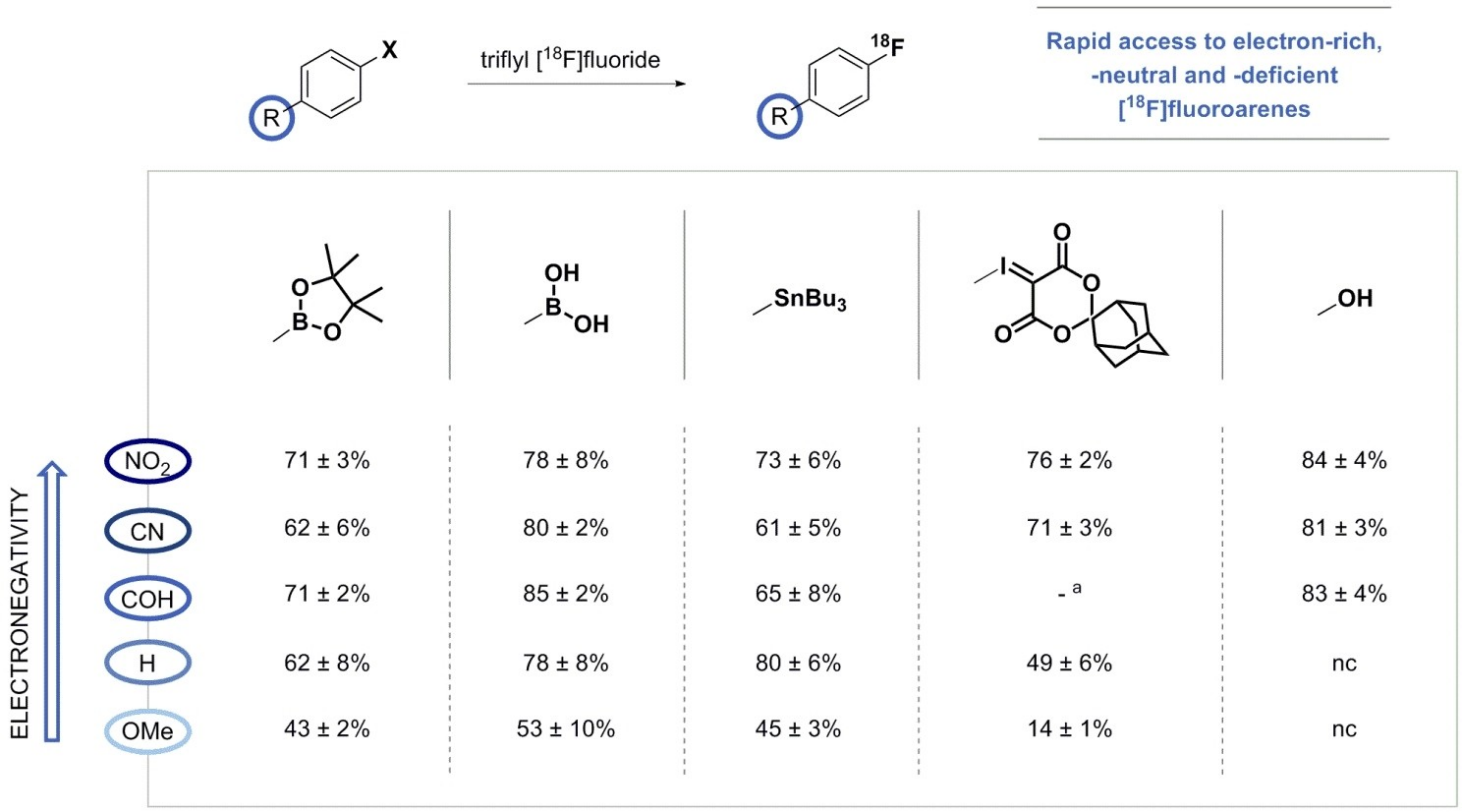
Chemical scope of [^18^F]fluoride derived from triflyl [^18^F]fluoride. Radiochemical conversions were determined by radio‐HPLC of the crude mixtures. Reactions were performed at least three times (*n*=3) and are given in the form mean ± standard deviation. [a] Precursor could not be synthesized. nc=no conversion. See Supporting Information for experimental details.

To demonstrate the performance of the [^18^F]fluoride derived from triflyl [^18^F]fluoride, a small series of electronically different arenes was tested under the optimized conditions for each precursor (Figure 3). As shown, electronic effects did not have a large impact on the Cu‐mediated radiofluorination methods. Electron‐deficient and ‐neutral ^18^F‐labeled arenes were prepared in good RCCs exceeding or averaging 60 %, where the presence of electron‐donating groups was tolerated, resulting in moderate‐to‐good RCCs (43–53 %). The electron‐deficient stannane substrates are noteworthy, as they are amongst the most challenging precursors for ^18^F‐labeling using this approach.^[9]^ Compared to previously disclosed low‐base Cu‐mediated ^18^F‐fluorination reactions, e. g. work‐up procedures with eluents containing alcohol (minimalist approach), weak bases or low amounts of strong bases, the obtained RCCs are comparable, but consistently more reliable.[^8^, ^18^, ^21^, ^22^, ^23^, ^24^] In addition, electron‐neutral aryliodonium ylides proved to be suitable substrates for the optimized ^18^F‐labeling strategy resulting in moderate yields (49±6 % RCC), whereas electron‐rich ^18^F‐labeled substrates were difficult to obtain (<20 % RCC), although in comparable RCCs as previously reported.^[20]^ Activated arenes displaying electron‐withdrawing substituents were successfully radiofluorinated in reproducible and high yields (>70 % RCC), which is in line with the lower barrier to reductive elimination of the corresponding fluoroarenes.^[10]^ Unfortunately, it was not possible to obtain the *para‐*substituted benzaldehyde derivative of the ylide. Finally, activated hydroxyarenes underwent radio‐deoxyfluorination in almost quantitative RCCs, whereas electron‐neutral and ‐rich substrates like anisole showed no radiochemical conversion as was expected.^[11]^ In general, the disadvantages of previously disclosed low‐base radiofluorination procedures, i. e. low elution efficiencies, the need for a drying step or difficult dissolution of the reactive [^18^F]F^−^ species, have been overcome with the use of triflyl [^18^F]fluoride.[^8^, ^18^, ^21^, ^22^]

### Application in the Synthesis of Clinically Relevant Radiopharmaceuticals

The use of triflyl [^18^F]fluoride for the synthesis of tracers to be used in (pre)clinical research studies requires upscaling of the procedure, where the whole batch of cyclotron produced [^18^F]fluoride is used to make triflyl [^18^F]fluoride. The scale‐up was found to be problematic due to the diminution of the amounts of K_2_CO_3_/K_222_ in the trapping of the reactive [^18^F]fluoride generated from triflyl [^18^F]fluoride. Concentrated amounts of K_2_CO_3_/K_222_ for the trapping followed by dilution of the K_2_CO_3_/K_222_ concentration upon addition of the precursor were identified as the most suitable conditions (see Table S12). Generally, quantitative trapping was obtained with 7.5/15 μmol of K_2_CO_3_/K_222_ in 300 μL solvent. Near quantitative conversions were obtained after the addition of Ph‐Bpin, Ph‐SnBu_3_ and *p*‐cyanophenol and their additives to reach a final volume of 1.2 mL with the same reaction molarities found during the condition screening for the respective precursor classes (see Table S13). In the case of Ph−B(OH)_2_, increasing the amount of Cu(OTf)_2_ from 6.0 μmol to 12–24 μmol was necessary to maintain moderate conversions (42±5 %) as a consequence of the degradation of Cu(OTf)_2_ in the presence of higher concentrations of base and cryptand. In addition, quantitative trapping in DMF required doubling the amount of base/cryptand and solvent to 15/30 μmol of K_2_CO_3_/K_222_ in 600 μL DMF. Interestingly, subsequent addition of Ph−I[SPIAd] in DMF without TEAB as the base still provided good conversions (83±6 %). It is noted that precursor amounts up to 60 μmol are not desirable for clinical productions of radiotracers. However, the high amounts of precursor may not be necessary to obtain conversions that are sufficient for (pre)clinical studies. If desirable, decreasing the amount of precursor should be studied in these specific cases.

**Scheme 1:**
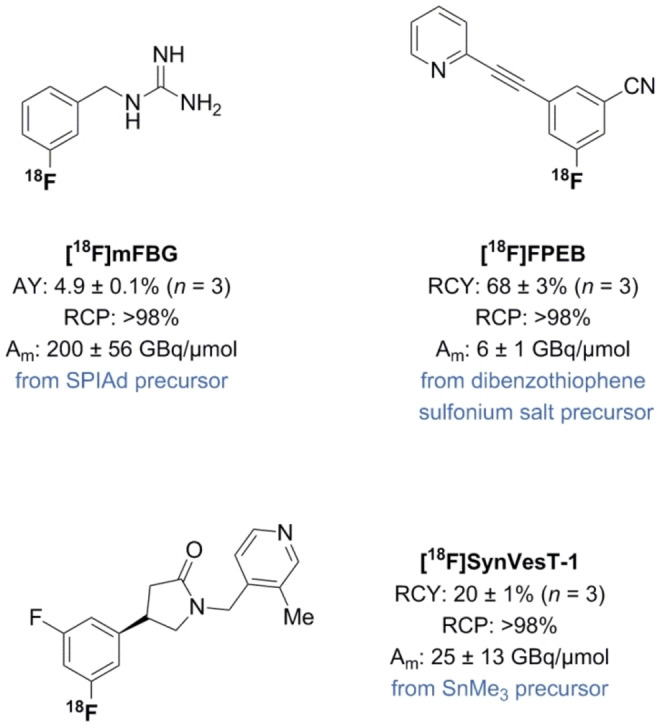
Application of triflyl [^18^F]fluoride to late‐stage ^18^F‐fluorination of clinically relevant PET tracers. See Supporting Information for experimental details.

With the aim to demonstrate practicability of the triflyl [^18^F]fluoride method, the clinically relevant PET tracers (*R*)‐4‐(3‐Fluoro‐5‐(fluoro‐^18^F)phenyl)‐1‐((3‐methylpyridin‐4‐yl)‐methyl)pyrrolidin‐2‐one ([^18^F]SynVesT‐1), 3‐[^18^F]fluoro‐5‐[(pyridin‐3‐yl)‐ethynyl]benzonitrile ([^18^F]FPEB) and *meta*‐[^18^F]fluorobenzylguanidine ([^18^F]*m*FBG) were synthesized (Scheme 1).

[^18^F]SynVesT‐1 is a potent and selective synaptic vesicle glycoprotein 2 (SV2 A) PET tracer for the quantification of synaptic density in in the brain.^[25]^ [^18^F]SynVesT‐1 is produced for clinical use via the Cu‐mediated radiofluorination of the trimethyltin precursor with Cu(OTf)_2_/py as catalyst. With small adjustments to the optimized triflyl [^18^F]fluoride conditions for Ph‐SnBu_3_, 6.7 μmol SnMe_3_‐SynVesT‐1 and 13 μmol Cu(OTf)_2_(py)_4_ in DMA/*n*BuOH (2 : 1) at 110 °C gave direct access to [^18^F]SynVesT‐1 in 20±1 % RCY (*n*=3, Scheme 1). From ~5 GBq (end‐of‐bombardment), the molar activity of the tracer was 25±13 GBq/μmol. In order to limit the use of precursor, the trapping of triflyl [^18^F]fluoride was performed with low amounts of K_2_CO_3_/K_222_ in MeCN, which was evaporated before adding the reagents. These unoptimized results are similar to the existing clinical production protocols indicating the generality of the conditions obtained after optimization for the different substrate classes.

In addition, [^18^F]*m*FBG is a structural and functional analog of norepinephrine with applications in oncology and cardiac imaging.^[10]^ The practical utility of triflyl [^18^F]fluoride in the ^18^F‐labeling of spirocyclic iodonium ylides has been established by the fully automated, good manufacturing practice (GMP) compliant preparation of [^18^F]*m*FBG using a solution of 7.5/15 μmol K_2_CO_3_/K_222_ in MeCN as base instead of an aqueous solution of TBAHCO_3_. This replacement resulted in a 30 minutes shorter synthesis time (120 min vs 90 min) with similar isolated yields (~5 % AY) by omitting the azeotropic drying step. The radiolabeling reaction is preferentially performed in a small volume of DMF or DMSO to prevent challenges during semi‐preparative HPLC purification and to ensure high concentrations of reagents without having to use fairly large amounts of expensive precursor. To this end, the triflyl [^18^F]fluoride was trapped in MeCN, which was then evaporated. The fully automated, GMP compliant production obtained [^18^ F]*m*FBG in a total synthesis time of ~90 min in 4.9±0.1 % AY and with high molar activity (200±56 GBq/μmol, *n* =3, Scheme 1). Compared to clinically validated synthesis procedures of [^18^F]*m*FBG, the RCY and *A*_m_ obtained with triflyl [^18^F]fluoride are equally as good as those reported by Wang *et al*. (RCY: 6.0±1.4 %, *A*_m_: 59.0±35.9 GBq/μmol, *n*=10) and Pauwels *et al*. (AY: 10.2±2.7 %, *A*_m_: 128±53 GBq/μmol, 80 min, *n*=6).[^26^, ^27^] Hence under non‐optimized conditions, the triflyl [^18^F]fluoride with K_2_CO_3_/K_222_ method could fastly and reliably obtain [^18^F]*m*FBG in activity yields suitable for clinical use.

Finally, [^18^F]FPEB is a PET radiotracer used for imaging metabotropic glutamate 5 receptors.^[15]^ This radioligand is particularly pertinent as previous attempts at preparing the tracer upon treatment of boron, stannane and spirocyclic iodonium ylide precursors provided the product in modest RCYs (4−20 %).[^8^, ^15^, ^28^] Attempted labeling of the dibenzothiophene sulfonium salt precursor under conditions from Gendron *et al*. gave [^18^F]FPEB in 68±3 % RCY with a molar activity of 5.7±1 GBq/μmol (*n*=3, Scheme 1) starting from ~5 GBq (end‐of‐bombardment).^[29]^ Compared to the described procedure (RCY: 55±3 %, *A*_m_: 13–20 GBq/μmol), a higher RCY in a shorter time was obtained using triflyl [^18^F]fluoride instead of azeotropic drying. As the reaction takes place under conditions that mirror radiofluorination reactions with azeotropically dried [^18^F]fluoride, the triflyl [^18^F]fluoride derived [^18^F]fluoride can thus readily be applied using established reaction conditions for the ^18^F‐radiolabeling and purification of existing and new clinical PET tracers.

## Conclusions

In conclusion, this study demonstrates that gaseous triflyl [^18^F]fluoride is a versatile [^18^F]fluoride source for late‐stage nucleophilic aromatic ^18^F‐fluorination reactions that offers several advantages over conventional methods. Most important are the reduced amounts of base and cryptand that led to improved yields and reliability in the radiosynthesis using arylboron and ‐stannane, hypervalent iodonium ylide and phenol substrates as well as in shorter reaction times for the preparation of the clinically relevant PET tracers [^18^F]FPEB, [^18^F]*m*FBG and [^18^F]SynVesT‐1. Generally, *n*BuOH as co‐solvent was advantageous in Cu‐mediated ^18^F‐fluorination reactions, but a deeper understanding of the crucial conditions for arylboron substrates is needed in order to obtain robust reaction conditions. In addition, the ^18^F‐deoxyfluorination reaction with triflyl [^18^F]fluoride was robust for activated substrates, operationally simple and could be executed in air and in the presence of moisture. Overall, these findings demonstrate the advantages of the use of triflyl [^18^F]fluoride in late‐stage aromatic ^18^F‐fluorination reactions, resulting in simplified, efficient and more reliable radiosynthetic methodologies.

## Experimental Section

All experimental procedures and data are given in the Supporting Information.

## Conflict of Interests

The authors declare no conflict of interest.

1

## Supporting information

As a service to our authors and readers, this journal provides supporting information supplied by the authors. Such materials are peer reviewed and may be re‐organized for online delivery, but are not copy‐edited or typeset. Technical support issues arising from supporting information (other than missing files) should be addressed to the authors.

Supporting Information

## Data Availability

The data that support the findings of this study are available in the supplementary material of this article.
