# Supplementary material for: Triflyl [18F]Fluoride as a Solution for Base‐Sensitive Late‐Stage Nucleophilic Aromatic 18F‐Fluorination Reactions
Source: Chemistry. 2024 Nov 26;31(2):e202403127. doi: 10.1002/chem.202403127 (PMC11724229; doi:10.1002/chem.202403127)
Supplement: Supplementary file 1 — Supporting Information [file CHEM-31-e202403127-s001.pdf]

Chemistry–A European Journal

Supporting Information

Triflyl [^{18}F]Fluoride as a Solution for Base-Sensitive Late-Stage Nucleophilic Aromatic ^{18}F-Fluorination Reactions

Lizeth Y. F. Haveman,* Anna M. T. de Kruijff, Sjoerd P. P. van Eeden, Albert D. Windhorst, and Danielle J. Vugts*

Supporting Information for:

Triflyl [^{18}F]Fluoride as a Solution for Base-sensitive Late-stage Nucleophilic Aromatic ^{18}F-Fluorination Reactions

Lizeth Y.F. Haveman,*[a] Anna M.T. de Kruijff,[a] Sjoerd P.P. van Eeden,[a] Albert D. Windhorst,[a] and Danielle J. Vugts.[a]

[a] Radiology & Nuclear Medicine
Amsterdam UMC location Vrije Universiteit Amsterdam,
De Boelelaan 1117, Amsterdam, The Netherlands
E-mail: l.havema1@amsterdamumc.nl; d.vugts@amsterdamumc.nl

Table of Contents

1. General information **2**

2. Radiochemistry **3**

2.1 [^{18}F]fluoride production 3

2.2 Optimization of reaction conditions for each substrate class 3

2.3 Investigation of the reaction scope 7

2.4 Optimization of one-pot ^{18}F-labeling 8

2.5 ^{18}F-labeling of clinical tracers 9

3. Organic chemistry **11**

3.1 Synthesis and characterization of arylstannane precursors 11

3.2 Synthesis and characterization of aryl iodonium ylide precursors 11

3.3 Synthesis and characterization of SynVesT-1 precursor and reference 13

3.4 Characterization of mFBG and FPEB precursor and reference 16

4. HPLC chromatograms **17**

5. ^{1}H and ^{13}C NMR Spectra **22**

6. References **42**

1. General information

Unless otherwise stated, chemicals were purchased from Sigma Aldrich (Zwijndrecht, The Netherlands) or Fluorochem (Derbyshire, United Kingdom) and used as received. The FPEB precursor and reference were kindly provided by Dr. Erik Arstad (University College London, London, United Kingdom). All solvents were purchased from Fisher Scientific (Landsmeer, the Netherlands) dried and degassed and used as received. Water was distilled and deionised by means of a Milli-Q water filtration system (Millipore, USA). Certified 2 mL clear screw top HPLC vials (12 x 32 mm) with write-on spot and screw caps (PTFE/red silicone septa, blue caps) were purchased from Agilent (Middelburg, The Netherlands). ^{1}H and ^{13}C NMR spectra were recorded on a Bruker Avance 500 spectrometer at 20°C. NMR spectra were processed using MestReNova 14.2 software. Chemical shifts are reported as dimensionless δ-values in parts per million (ppm) relative to the solvent: $CDCl_3$ (^{1}H = δ 7.26, ^{13}C = δ 77.2), DMSO-d_6 (^{1}H = δ 2.50, ^{13}C = δ 39.5), MeCN-d_3 (^{1}H = δ 1.94, ^{13}C = δ 1.3 and 118.3) and D_2O (^{1}H = δ 4.79). Coupling constants (J) are reported in units of hertz (Hz). The following abbreviations are used to describe multiplicities: s (singlet), d (doublet), t (triplet), m (multiplet). Purities were measured with the aid of analytical LC−MS using a Shimadzu LC-20AD liquid chromatography pump system (Shimadzu, 's Hertogenbosch, The Netherlands) with a Shimadzu SPDM20A diode array detector (at 254 nm) and a Shimadzu LC-MS-2010EV mass spectrometer operating in both positive and negative ionization mode. The column used for the LC-MS analysis was a Waters Xbridge (C_{18}) 5 μm column (50 mm × 4.6 mm) with the following solutions as eluents. Solvent A: water/formic acid 999:1 and solvent B: acetonitrile/formic acid 999:1. The eluent program used was as follows: flow rate: 1.0 mL/min, start 95% A in a linear gradient to 10% A over 4.5 min, hold 1.5 min at 10% A, in 0.5 min in a linear gradient to 95% A, hold 1.5 min at 95% A, total run time: 8.0 min. High resolution mass spectra (HRMS, *m/z*) analyses were conducted on a Bruker impact II LC-QTOF system (Capillary voltage: -4500 V) using positive (ESI+) or negative electrospray ionization (ESI−) and on a Jeol JMST100GCv (AccuTOF GCv 4G) instrument using FI and EI. Thin-layer chromatography (TLC) was performed using TLC plates from Merck (SiO_2, neutral kieselgel 60 F254). TLC visualization was carried out with ultraviolet light at 254 nm and if necessarily followed by staining with a 1% aqueous KMnO4 solution. Flash column chromatography was performed on a Büchi Sepacore® X10 flash system (Büchi, Hendrik-Ido-Ambacht, The Netherlands) using silica packed cartridges or carried out with Merck silica gel (pore size 60 Å, 230-400 mesh particle size). All radioactive reactions and products were analyzed with high-performance liquid chromatography (HPLC) using a Shimadzu LC-20AT liquid chromatography module with a SIL-20A HT auto sampler, Shimadzu SPD-20A UV/VIS detector, CTO-20A column oven, DGU-20A5R degassing unit, CBM-20A communication bus module, a Elysia Raytest GABI Nova radio detector and LabSolutions 5.85 software (Shimadzu Corporation, Japan). All compounds were analyzed on a Phenomenex C_{18}, 250 x 4.6 mm, 5 μm (VWR, The Netherlands) using eluent A) 65:35 MeCN/H_2O or eluent B) 50:50 MeCN/H_2O at a flow of 1 mL/min. UV active compounds were detected at 254 nm. Radioactive products were identified by comparison with the unlabeled reference compounds. Radioactivity was quantified with a Veenstra VDC-304 dose calibrator (Veenstra, Joure, Friesland). Radiopharmaceutical terminology, i.e. radiochemical terms, symbols and expressions follow the rules for nomenclature good practice.[1,2]

2. Radiochemistry

2.1 [^{18}F]fluoride production

[^{18}F]fluoride was produced by the $^{18}O(p,n)^{18}F$ nuclear reaction on an IBA Cyclone® 18/9 cyclotron using a [^{18}O]H_2O liquid target. After irradiation, the target water was passed through a Chromafix® 30-PS-HCO_3 cartridge to trap the [^{18}F]fluoride. [^{18}F]fluoride was eluted from the cartridge with a 0.1 M potassium sulfate solution (500 µL) into a reaction vessel containing DMF (850 µL). *N,N*-bis(trifluoromethylsulfonyl)aniline (150 µL, 0.1 M) was added to the vessel and the mixture was heated at 40 °C. The formed gaseous triflyl [^{18}F]fluoride was continuously distilled over phosphorus pentoxide into a second reaction vessel with a gentle stream of helium (10 mL/min). The triflyl [^{18}F]fluoride was trapped in a solvent of choice in the presence or absence of cryptand and base at -40 or -60 °C.

2.2 Optimization of reaction conditions for each substrate class

In a typical reaction, a 50 µL aliquot of [^{18}F]KF/K_{222} (~50-400 MBq) was added to a HPLC-vial. A solution of precursor and catalyst was added via a syringe. The sealed vial was heated. The reaction was quenched by addition of water (200 µL). An aliquot of the reaction mixtures was subjected to radioHPLC analysis to determine the radiochemical conversion and product identity. HPLC condition A) was used for R = Ph-Bpin, Ph-B$(OH)_2$, Ph-SnBu3, Ph-I[SPIAd] and B) for R = PhOH. The accuracy of the RCC determined by HPLC was confirmed by radio-TLC analysis.

Table S1 Variation of reaction time, temperature, solvent and precursor and catalyst amount for [^{18}F]fluorobenzene formation from Ph-Bpin.

Entry	Temperature °C	Time min	Solvent	Precursor µmol	$Cu(OTf)_2(py)_4$ µmol	RCC %
**REF**	110	20	DMF	60	5.0	**39 ± 24***
**1**	70	20	DMF	60	5.0	**16 ± 1**
**2**	80	20	DMF	60	5.0	**29 ± 3**
**3**	90	20	DMF	60	5.0	**34 ± 3**
**4**	100	20	DMF	60	5.0	**37 ± 2**
**5**	120	20	DMF	60	5.0	**22 ± 13**
**6**	130	20	DMF	60	5.0	**19 ± 6**
**7**	110	5	DMF	60	5.0	**18 ± 5**
**8**	110	10	DMF	60	5.0	**21 ± 11**
**9**	110	15	DMF	60	5.0	**22 ± 11**
**10**	110	20	DMA	60	5.0	**31 ± 8**
**11**	110	20	NMP	60	5.0	**32 ± 2**
**12**	110	20	DMF	30	5.0	**27 ± 5**
**13**	110	20	DMF	20	5.0	**35 ± 13**
**14**	110	20	DMF	10	5.0	**25 ± 12**
**15**	110	20	DMF	5.0	5.0	**9 ± 4**
**16**	110	20	DMF	20	10	**38 ± 2**
**17**	110	20	DMF	20	20	**43 ± 2**
**18**	110	20	DMF	20	30	**34 ± 4**
**19**	110	20	DMA/nBuOH (2:1)	20	20	**76 ± 2**

Reaction conditions: Ph-Bpin, $Cu(OTf)_2(py)_4$, 0.50/1.0 µmol K_2CO_3/K_{222}; total reaction volume: 400 µL. RCC is given in average ± SD, dc, *n* = 3 (* *n* = 18), RCC = yield of the product determined by analysis of the crude mixture by HPLC.

Table S2 Variation of Cu catalyst, water and K_2CO_3/K_{222} amount, addition order and reaction atmosphere for [^{18}F]fluorobenzene formation from Ph-Bpin.

Entry	Cu	Water µmol	K_2CO_3/K_{222} µmol	Order [b]	Atmosphere [c]	RCC %
**1**	$Cu(OTf)_2(py)_4$	0	0.50/1.0	Standard	Ambient	**37 ± 2**
**2** [a]	$Cu(OTf)_2$/py	0	0.50/1.0	Standard	Ambient	**22 ± 2**
**3**	$Cu(OTf)_2(py)_4$	1.0	0.50/1.0	Standard	Ambient	**38 ± 3**
**4**	$Cu(OTf)_2(py)_4$	5.0	0.50/1.0	Standard	Ambient	**23 ± 5**
**5**	$Cu(OTf)_2(py)_4$	0	-	Standard	Ambient	**24 ± 4**
**6**	$Cu(OTf)_2(py)_4$	0	1.0/2.0	Standard	Ambient	**32 ± 2**
**7**	$Cu(OTf)_2(py)_4$	0	4.0/8.0	Standard	Ambient	**19 ± 3**
**8**	$Cu(OTf)_2(py)_4$	0	0.50/1.0	Direct	Ambient	**31 ± 3**
**9**	$Cu(OTf)_2(py)_4$	0	0.50/1.0	Standard	O_2	**39 ± 5**
**10**	$Cu(OTf)_2(py)_4$	0	0.50/1.0	Standard	N_2	**18 ± 1**

Reaction conditions: 20 µmol Ph-Bpin, 20 µmol Cu, DMF, 20 min, 110 °C; total reaction volume: 400 µL. RCC is given in average ± SD, dc, *n* = 3, RCC = yield of the product determined by analysis of the crude mixture by HPLC. [a] 500 µmol pyridine. [b] Standard = HPLC vial with solvent to which a [^{18}F]KF/K_{222} aliquot was added, then a solution of the additive and finally a solution of the precursor; direct = HPLC vial a solution of additive and precursor to which a [^{18}F]KF/K_{222} aliquot was added. [c] Prior to the reaction, the vial was purged with 5 mL of air, O_2 or dried N_2.

Table S3 Variation of reaction time, temperature, solvent and precursor and catalyst amount for [^{18}F]fluorobenzene formation from $Ph\text{-}B(OH)_2$.

Entry	Temperature °C	Time min	Solvent	Precursor µmol	$Cu(OTf)_2$/py µmol	RCC %
**REF**	110	20	DMF	4.0	12/500	**46 ± 24***
**1**	70	20	DMF	4.0	12/500	**1 ± 1**
**2**	80	20	DMF	4.0	12/500	**5 ± 2**
**3**	90	20	DMF	4.0	12/500	**15 ± 4**
**4**	100	20	DMF	4.0	12/500	**38 ± 3**
**5**	120	20	DMF	4.0	12/500	**37 ± 7**
**6**	130	20	DMF	4.0	12/500	**37 ± 4**
**7**	110	5	DMF	4.0	12/500	**30 ± 3**
**8**	110	10	DMF	4.0	12/500	**29 ± 7**
**9**	110	15	DMF	4.0	12/500	**30 ± 5**
**10**	110	20	DMA	4.0	12/500	**19 ± 17**
**11**	110	20	NMP	4.0	12/500	**6 ± 2**
**12**	110	20	DMF	2.0	12/500	**24 ± 3**
**13**	110	20	DMF	6.0	12/500	**38 ± 5**
**14**	110	20	DMF	8.0	12/500	**34 ± 11**
**15**	110	20	DMF	12	12/500	**22 ± 5**
**16**	110	20	DMF	6.0	6.0/250	**41 ± 1**
**17**	110	20	DMF	6.0	3.0/125	**32 ± 6**
**18**	110	20	DMA/nBuOH (2:1)	6.0	6.0/250	**84 ± 3**

Reaction conditions: $Ph\text{-}B(OH)_2$, $Cu(OTf)_2$, pyridine, 0.50/1.0 µmol K_2CO_3/K_{222}; total reaction volume: 1.0 mL. RCC is given in average ± SD, dc, *n* = 3 (* *n* = 19), RCC = yield of the product determined by analysis of the crude mixture by HPLC.

Table S4 Variation of Cu catalyst, water and K_2CO_3/K_{222} amount, addition order and reaction atmosphere for [^{18}F]fluorobenzene formation from $Ph\text{-}B(OH)_2$.

Entry	Cu	Water µmol	K_2CO_3/K_{222} µmol	Order [a]	Atmosphere [b]	RCC [c] %
**1**	$Cu(OTf)_2$/py	0	0.50/1.0	Standard	Ambient	**48 ± 2**
**2**	$Cu(OTf)_2(py)_4$	0	0.50/1.0	Standard	Ambient	**39 ± 8**
**3**	$Cu(OTf)_2$/py	1.0	0.50/1.0	Standard	Ambient	**35 ± 3**
**4**	$Cu(OTf)_2$/py	5.0	0.50/1.0	Standard	Ambient	**22 ± 2**
**5**	$Cu(OTf)_2$/py	0	-	Standard	Ambient	**nd**
**6**	$Cu(OTf)_2$/py	0	1.0/2.0	Standard	Ambient	**34 ± 8**
**7**	$Cu(OTf)_2$/py	0	4.0/8.0	Standard	Ambient	**1 ± 1**
**8**	$Cu(OTf)_2$/py	0	0.50/1.0	Direct	Ambient	**44 ± 14**
**9**	$Cu(OTf)_2$/py	0	0.50/1.0	Standard	N_2	**13 ± 2**

Reaction conditions: 6.0 µmol Ph-$B(OH)_2$, 6.0 µmol Cu, DMF, 20 min, 110 °C; total reaction volume: 1.0 mL. RCC is given in average ± SD, dc, *n* = 3, RCC = yield of the product determined by analysis of the crude mixture by HPLC. [a] Standard = HPLC vial with solvent to which a [^{18}F]KF/K_{222} aliquot was added, then a solution of the additive and finally a solution of the precursor; direct = HPLC vial with a solution of additive and precursor to which a [^{18}F]KF/K_{222} aliquot was added. [b] Prior to the reaction, the vial was purged with 5 mL of dried N_2. [c] desired product was not detected.

Table S5 Variation of reaction time, temperature, solvent and precursor and catalyst amount for [^{18}F]fluorobenzene formation from Ph-$SnBu_3$.

Entry	Temperature °C	Time min	Solvent	Precursor µmol	$Cu(OTf)_2(py)_4$ µmol	RCC [a] %
**REF**	110	10	DMA	30	30	**55 ± 9***
**1**	70	10	DMA	30	30	**nd**
**2**	80	10	DMA	30	30	**13 ± 3**
**3**	90	10	DMA	30	30	**25 ± 7**
**4**	100	10	DMA	30	30	**41 ± 6**
**5**	120	10	DMA	30	30	**62 ± 7**
**6**	130	10	DMA	30	30	**49 ± 4**
**7**	120	5	DMA	30	30	**52 ± 6**
**8**	120	15	DMA	30	30	**61 ± 6**
**9**	120	15	DMF	30	30	**17 ± 2**
**10**	120	15	NMP	30	30	**61 ± 3**
**11**	120	15	DMA	5.0	30	**37 ± 4**
**12**	120	15	DMA	10	30	**65 ± 7**
**13**	120	15	DMA	20	30	**69 ± 9**
**14**	120	15	DMA	20	10	**32 ± 4**
**15**	120	15	DMA	20	20	**52 ± 9**
**16**	120	15	DMA	20	30	**69 ± 9**
**17**	120	15	DMA	20	40	**68 ± 2**
**18**	120	15	DMA	20	60	**70 ± 10**
**19**	120	15	DMA	20	80	**65 ± 10**
**20**	120	15	DMA/nBuOH (2:1)	20	40	**80 ± 4**

Reaction conditions: Ph-$SnBu_3$, $Cu(OTf)_2(py)_4$, 0.50/1.0 µmol K_2CO_3/K_{222}; total reaction volume: 1.0 mL. RCC is given in average ± SD, dc, *n* = 3 (* *n* = 11), RCC = yield of the product determined by analysis of the crude mixture by HPLC. [a] desired product was not detected.

Table S6 Variation of Cu catalyst, water and K_2CO_3/K_{222} amount, addition order and reaction atmosphere for [^{18}F]fluorobenzene formation from Ph-$SnBu_3$.

Entry	Cu	Water µmol	K_2CO_3/K_{222} µmol	Order [b]	Atmosphere [c]	RCC %
**1**	$Cu(OTf)_2(py)_4$	0	0.50/1.0	Standard	Ambient	**68 ± 2**
**2** [a]	$Cu(OTf)_2$/py	0	0.50/1.0	Standard	Ambient	**36 ± 6**

**3**	$Cu(OTf)_2(py)_4$	1.0	0.50/1.0	Standard	Ambient	**62 ± 5**
**4**	$Cu(OTf)_2(py)_4$	5.0	0.50/1.0	Standard	Ambient	**51 ± 6**
**5**	$Cu(OTf)_2(py)_4$	0	-	Standard	Ambient	**29 ± 5**
**6**	$Cu(OTf)_2(py)_4$	0	1.0/2.0	Standard	Ambient	**59 ± 3**
**7**	$Cu(OTf)_2(py)_4$	0	4.0/8.0	Standard	Ambient	**16 ± 3**
**8**	$Cu(OTf)_2(py)_4$	0	0.50/1.0	Direct	Ambient	**60 ± 2**
**9**	$Cu(OTf)_2(py)_4$	0	0.50/1.0	Standard	N_2	**57 ± 1**

Reaction conditions: 20 µmol Ph-$SnBu_3$, 40 µmol $Cu(OTf)_2(py)_4$ or $Cu(OTf)_2$, DMA, 10 min, 120 °C; total reaction volume: 1.0 mL. RCC is given in average ± SD, dc, *n* = 3, RCC = yield of the product determined by analysis of the crude mixture by HPLC. [a] 300 µmol pyridine. [b] Standard = HPLC vial with solvent to which a [^{18}F]KF/K_{222} aliquot was added, then a solution of the additive and finally a solution of the precursor; direct = HPLC vial with a solution of additive and precursor to which a [^{18}F]KF/K_{222} aliquot was added. [c] Prior to the reaction, the vial was purged with 5 mL of dried N_2.

Table S7 Variation of reaction time, temperature, solvent and precursor and catalyst amount for [^{18}F]fluorobenzene formation from Ph-I[SPIAd].

Entry	Temperature °C	Time min	Solvent	Precursor µmol	TEAB µmol	RCC %
**REF**	120	10	DMF	4.0	40	**34 ± 28***
**1**	80	10	DMF	4.0	40	**3 ± 1**
**2**	90	10	DMF	4.0	40	**12 ± 2**
**3**	100	10	DMF	4.0	40	**18 ± 2**
**4**	110	10	DMF	4.0	40	**21 ± 3**
**5**	130	10	DMF	4.0	40	**31 ± 4**
**6**	140	10	DMF	4.0	40	**34 ± 8**
**7**	150	10	DMF	4.0	40	**26 ± 5**
**8**	160	10	DMF	4.0	40	**16 ± 4**
**9**	140	5	DMF	4.0	40	**22 ± 3**
**10**	140	15	DMF	4.0	40	**29 ± 3**
**11**	140	10	MeCN	4.0	40	**3 ± 5**
**12**	140	10	DMSO	4.0	40	**38 ± 6**
**13**	140	10	DMF	2.0	40	**11 ± 3**
**14**	140	10	DMF	8.0	40	**41 ± 4**
**15**	140	10	DMF	16	40	**48 ± 4**
**16**	140	10	DMF	24	40	**55 ± 7**
**17**	140	10	DMF	32	40	**61 ± 14**
**18**	140	10	DMF	8.0	20	**41 ± 1**
**19**	140	10	DMF	8.0	10	**36 ± 3**
**20**	140	10	DMF	8.0	-	**31 ± 2**

Reaction conditions: Ph-I[SPIAd], tetrabutylammonium bicarbonate, 0.50/1.0 µmol K_2CO_3/K_{222}; total reaction volume: 400 µL. RCC is given in average ± SD, dc, *n* = 3 (* *n* = 6), RCC = yield of the product determined by analysis of the crude mixture by HPLC.

Table S8 Variation of TEAB, water and K_2CO_3/K_{222} amount, addition order and reaction atmosphere for [^{18}F]fluorobenzene formation from Ph-I[SPIAd].

Entry	TEAB µmol	Water µmol	K_2CO_3/K_{222} µmol	Order [a]	Atmosphere [b]	RCC %
**1**	40	0	0.50/1.0	Direct	Ambient	**41 ± 4**
**2**	40	1.0	0.50/1.0	Direct	Ambient	**40 ± 5**
**3**	40	5.0	0.50/1.0	Direct	Ambient	**43 ± 3**
**4**	-	0	-	Direct	Ambient	**5 ± 2**
**5**	-	0	0.50/1.0	Direct	Ambient	**31 ± 2**
**6**	-	0	1.0/2.0	Direct	Ambient	**34 ± 1**
**7**	-	0	4.0/8.0	Direct	Ambient	**49 ± 5**

**8**	-	0	16/32	Direct	Ambient	**41 ± 3**
**9**	40	0	0.50/1.0	Standard	Ambient	**29 ± 5**
**10**	40	0	0.50/1.0	Direct	N_2	**25 ± 1**

Reaction conditions: 8.0 µmol Ph-I[SPIAd], DMF, 10 min, 140 °C; total reaction volume: 400 µL. RCC is given in average ± SD, dc, *n* = 3, RCC = yield of the product determined by analysis of the crude mixture by HPLC. [a] Standard = HPLC vial with TEAB and solvent to which a [^{18}F]KF/K_{222} aliquot was added, then a solution of the precursor; direct = HPLC vial with a solution of TEAB and precursor to which a [^{18}F]KF/K_{222} aliquot was added.

Table S9 Variation of reaction time, temperature, solvent and precursor and catalyst amount for 4-[^{18}F]fluorobenzonitrile formation from 4-cyanophenol.

Entry	Temperature °C	Time min	Solvent	Precursor µmol	$ImCl^+/Ag_2CO_3$ µmol	RCC %
**REF**	130	20	DMSO/MeCN (1:1)	10	10/5.0	**85 ± 3**
**1**	90	20	DMSO/MeCN (1:1)	10	10/5.0	**34 ± 5**
**2**	100	20	DMSO/MeCN (1:1)	10	10/5.0	**50 ± 2**
**3**	110	20	DMSO/MeCN (1:1)	10	10/5.0	**68 ± 1**
**4**	120	20	DMSO/MeCN (1:1)	10	10/5.0	**85 ± 2**
**5**	140	20	DMSO/MeCN (1:1)	10	10/5.0	**80 ± 4**
**6**	150	20	DMSO/MeCN (1:1)	10	10/5.0	**82 ± 2**
**7**	130	15	DMSO/MeCN (1:1)	10	10/5.0	**77 ± 3**
**8**	130	10	DMSO/MeCN (1:1)	10	10/5.0	**68 ± 2**
**9**	130	20	DMF	10	10/5.0	**59 ± 10**
**10**	130	20	Butanone/EtOH (9:1)	10	10/5.0	**24 ± 5**
**11**	130	20	1,4-dioxane	10	10/5.0	**57 ± 19**
**12**	130	20	DMSO/MeCN (1:1)	5.0	10/5.0	**46 ± 9**
**13**	130	20	DMSO/MeCN (1:1)	20	10/5.0	**86 ± 1**
**14**	130	20	DMSO/MeCN (1:1)	10	5.0/2.5	**82 ± 5**
**15**	130	20	DMSO/MeCN (1:1)	10	20/10	**44 ± 5**
**16**	130	20	DMSO/MeCN (1:1)	5.0	5.0/2.5	**82 ± 8**

Reaction conditions: 4-cyanophenol, imidazolium chloride, silver carbonate, 0.50/1.0 µmol K_2CO_3/K_{222}; total reaction volume: 600 µL. RCC is given in average ± SD, dc, *n* = 3, RCC = yield of the product determined by analysis of the crude mixture by HPLC.

Table S10 Variation of water and K_2CO_3/K_{222} amount, addition order and reaction atmosphere for 4-[^{18}F]fluorobenzonitrile formation from 4-cyanophenol.

Entry	Water µmol	K_2CO_3/K_{222} µmol	Order [a]	Atmosphere [b]	RCC %
**1**	0	0.50/1.0	Standard	Ambient	**84 ± 2**
**2**	1.0	0.50/1.0	Standard	Ambient	**84 ± 3**
**3**	5.0	0.50/1.0	Standard	Ambient	**78 ± 2**
**4**	0	-	Standard	Ambient	**48 ± 21**
**5**	0	1.0/2.0	Standard	Ambient	**75 ± 8**
**6**	0	4.0/8.0	Standard	Ambient	**63 ± 16**
**7**	0	0.50/1.0	Direct	Ambient	**80 ± 9**
**8**	0	0.50/1.0	Standard	N_2	**56 ± 3**

Reaction conditions: 10 µmol 4-cyanophenol, 10 µmol imidazolium chloride, 5.0 µmol silver carbonate, DMSO/MeCN 1:1, 20 min, 130 °C; total reaction volume: 600 µL. RCC is given in average ± SD, dc, *n* = 3, RCC = yield of the product determined by analysis of the crude mixture by HPLC. [a] Standard = HPLC vial with solvent and Ag_2CO_3 to which a [^{18}F]KF/K_{222} aliquot was added, then a solution of the additive and finally a solution of the precursor; direct = HPLC vial with a suspension of Ag_2CO_3, additive and precursor to which a [^{18}F]KF/K_{222} aliquot was added. [b] Prior to the reaction, the vial was purged with 5 mL of dried N_2.

2.3 Investigation of the reaction scope

In a typical reaction, a 50 µL aliquot of [^{18}F]KF/K_{222} (~50-400 MBq) was added to a HPLC-vial containing a solvent and, if necessary, an additive. A solution of precursor and catalyst was added via syringe. The sealed vial was heated. The reaction was quenched by addition of water (200 µL). An aliquot of the reaction mixtures was subjected to radioHPLC analysis to determine the radiochemical conversion and product identity. HPLC condition A) was used for R = NO_2, CN and B) for R = COH, H, OMe.

Table S11 Reaction conditions used in the substrate scope for the different substrate classes.

Substrate class	HPLC vial	Syringe	Temperature	Time
**Aryl-Bpin**	130 µL nBuOH	20 µmol precursor<br>20 µmol $Cu(OTf)_2(py)_4$<br>220 µL DMA	110	20
**Aryl-B(OH)$_2$**	330 µL nBuOH	6.0 µmol precursor<br>6.0 µmol $Cu(OTf)_2$<br>250 µmol pyridine<br>600 µL DMA	110	20
**Aryl-SnMe$_3$**	330 µL nBuOH	20 µmol precursor<br>40 µmol $Cu(OTf)_2(py)_4$<br>620 µL DMA	120	15
**Aryl-I[aux]**	150 µL DMF<br>10 µmol TEAB	8.0 µmol precursor<br>200 µL DMF	140	10
**Aryl-OH**	300 µL MeCN<br>5.0 µmol Ag_2CO_3	10 µmol precursor<br>10 µmol Imidazolium chloride<br>250 µL DMSO	130	20

2.4 Optimization of one-pot ^{18}F-labeling

In a typical reaction, the triflyl [^{18}F]fluoride was trapped in a V-vial (2 mL) containing a solvent of choice in the presence or absence of a solution of cryptand and base at -40 °C. A solution of Ph-Bpin and $Cu(OTf)_2(py)_4$ was added via syringe. The sealed vial was heated at 110 °C for 20 minutes. The reaction was quenched by addition of water (500 µL). An aliquot of the reaction mixtures was subjected to radioHPLC analysis to determine the radiochemical conversion and product identity.

Table S12 Optimization of the reaction conditions of one-pot [^{18}F]fluorobenzene formation from Ph-Bpin.

Entry	K_2CO_3/K_{222} mM	Solvent	Volume µL	RCY_{trap} %	K_2CO_3/K_{222} mM	Volume µL	RCC_{rxn} %
**1**	1.7/3.3	DMA/nBuOH (2:1)	300	25 ± 1 (2)	1.3/2.5	400	**85 ± 1 (2)**
**2** [a]	1.7/3.3	DMA/nBuOH (2:1)	300	18	1.3/2.5	400	**42**
**3** [b]	1.7/3.3	DMA/nBuOH (2:1)	300	22	1.3/2.5	400	**39**
**4** [c]	1.7/3.3	MeCN	300	67	1.3/2.5	400	**81**
**5** [d]	1.7/3.3	DMA/nBuOH (2:1)	300	29	1.3/2.5	400	**93**
**6**	1.7/3.3	DMA/nBuOH (2:1)	900	39	1.3/2.5	1200	**58**
**7**	6.7/13	DMA/nBuOH (2:1)	300	34	5.0/10	400	**51**
**8**	13/27	DMA/nBuOH (2:1)	300	30	3.3/6.7	1200	**72**
**9**	27/53	DMA/nBuOH (2:1)	300	97	6.7/13	1200	**90**
**10**	25/50	DMA/nBuOH (2:1)	300	91 ± 1 (3)	6.3/13	1200	**93 ± 3 (3)**

Trapping conditions: K_2CO_3/K_{222} stock solution in 1.0 mL MeCN/H_2O (9:1), 2 mL reaction vessel; reaction conditions: 50 mM phenylboronic acid pinacol ester, 50 mM $Cu(OTf)_2(py)_4$, DMA/nBuOH (2:1), 20 min, 110 °C. RCY_{trap} = yield of the isolated [^{18}F]triflyl fluoride after distillation (dc), RCC_{rxn} = yield of the product determined by analysis of the crude mixture by HPLC. Analysis was performed using HPLC condition A. [a] 1 mL reaction vessel was used. [b]

Precursor and catalyst were in trapping solution. [c] MeCN was evaporated. [d] K_2CO_3/K_{222} stock solution was prepared in DMA.

Table S13 Reaction conditions used in the one-pot labeling of the model compounds for the different substrate classes.

Substrate	V-vial	Syringe	Temperature	Time	RCY %
**Ph-Bpin**	7.5/15 µmol K_2CO_3/K_{222}<br>275 µL DMA/nBuOH (2:1)	60 µmol precursor<br>60 µmol $Cu(OTf)_2(py)_4$<br>900 µL DMA/nBuOH (2:1)	110	20	**84 ± 3**
**Ph-B(OH)$_2$**	7.5/15 µmol K_2CO_3/K_{222}<br>275 µL DMA/nBuOH (2:1)	6.0 µmol precursor<br>24 µmol $Cu(OTf)_2$<br>600 µmol pyridine<br>850 µL DMA/nBuOH (2:1)	110	20	**42 ± 5**
**Ph-SnMe$_3$**	7.5/15 µmol K_2CO_3/K_{222}<br>275 µL DMA/nBuOH (2:1)	24 µmol precursor<br>48 µmol $Cu(OTf)_2(py)_4$<br>900 µL DMA	120	15	**85 ± 4**
**Ph-I[aux]**	15/30 µmol K_2CO_3/K_{222}<br>575 µL DMF	24 µmol precursor<br>600 µL DMF	140	10	**83 ± 6**
**4-cyanophenol**	7.5/15 µmol K_2CO_3/K_{222}<br>10 µmol Ag_2CO_3<br>275 µL MeCN	20 µmol precursor<br>20 µmol Imidazolium chloride<br>900 µL DMSO/MeCN (2:1)	130	20	**93 ± 7**

RCY is given in average ± SD, dc, n = 3, RCY = yield of the [^{18}F]triflyl fluoride after trapping x purity of the product determined by analysis of the crude mixture by radioHPLC. Analysis was performed using HPLC condition A except for 4-cyanophenol, where condition B was used.

2.5 ^{18}F-labeling of clinical tracers

4-(3,5-di[^{18}F]fluorophenyl)-1-((3-methylpyridin-4-yl)methyl)-pyrrolidin-2-one ([^{18}F]SynVesT-1)

Triflyl [^{18}F]fluoride, obtained from 5 GBq of cyclotron produced [^{18}F]fluoride, was trapped in a solution of MeCN (400 µL) with K_2CO_3 (0.50 µmol) and $K_{2.2.2}$ (1.0 µmol) at -40 °C. The solvent was evaporated with a stream of helium (25 mL/min) at 85 °C under vacuum. A solution of 4-(3-fluoro-5-(trimethylstannyl)phenyl)-1-((3-methylpyridin-4-yl)methyl)pyrrolidin-2-one (3.0 mg, 6.7 µmol) in DMA/*n*BuOH (2:1, 300 µL) and $Cu(OTf)_2(py)_4$ (9.1 mg, 13 µmol) in DMA/*n*BuOH (2:1, 300 µL) were subsequently added to the second vessel and the mixture was heated at 110 °C for 20 min. After addition of HPLC eluent (2 mL), the crude product was purified by semi-preparative HPLC (Phenomenex Luna C_{18}(2) 10 µm 10 x 250 mm; 0.1 M ammonium formate buffer + 0.5% acetic acid (pH 4.2)/MeCN 75:25; 5.0 mL/minute). The product in the isolated fraction was obtained with a radiochemical purity greater than 98% (Alltima C_{18} 5 µm 4.6 x 250 mm; 0.1 M ammonium formate buffer + 0.5% acetic acid (pH 4.2)/MeCN 66:34; 2.0 mL/minute). The radiochemical yield (corrected to the start of synthesis) was 20 ± 1% and the molar activity at the end of synthesis was 25 ± 13 GBq/µmol (n = 3).

[^{18}F]*meta*-fluorobenzylguanidine ([^{18}F]*m*FBG)

Triflyl [^{18}F]fluoride, obtained from 25 GBq of cyclotron produced [^{18}F]fluoride, was trapped in a solution of MeCN (1.0 mL) with $KHCO_3$ (7.5 µmol) and $K_{2.2.2}$ (15 µmol) at -40 °C. The solvent was evaporated. A solution of (1r,3r,5r,7r)-spiro[adamantane-2,2’-[1,3]dioxane]-4’,6’-dion-[3-((1,2,3,3-tetrakis(tert-butoxylcarbonyl)guanidino-3-iodonium)methyl)]ylide (8.0 mg, 8.8 µmol) in DMF (200 µL) was added to the second vessel and the mixture was heated at 120 °C for 5 min. The mixture was cooled to 75 °C and 6 M HCl (200 µL) was added, after which the reaction mixture was heated at 120 °C for 5 min. After cooling down the reaction mixture, it was diluted

with sterile water (1.5 mL) and subjected to semi-preparative HPLC (Alltima C_{18} 10 µm 22 x 250 mm; H_2O/MeOH/HCl 89:10:1; 10.0 mL/minute, λ = 210 nm). The collected radioactive fraction was diluted with sterile water (80 mL) and the total volume passed through two in series placed Oasis HLB Plus cartridges (preconditioned using 10 mL of EtOH and 10 mL of sterile water). After rinsing the cartridges with sterile water (10 mL), the product was eluted with ethanol (1.5 mL) into buffered saline (13.5 mL) and passed through a sterile filter into a crimped vial fitted with a vent needle. [^{18}F]mFBG was obtained in a total synthesis time of 1.5 h with a radiochemical purity greater than 98%. The activity yield was 4.9 ± 0.1% (*n* = 3) and the molar activity 200 ± 56 GBq/µmol at end of synthesis.

3-[^{18}F]fluoro-5-(pyridin-2-ylethynyl)benzonitrile ([^{18}F]FPEB)

Triflyl [^{18}F]fluoride, obtained from 5 GBq of cyclotron produced [^{18}F]fluoride, was trapped in a solution of MeCN (300 µL) with K_2CO_3 (7.5 µmol) and $K_{2.2.2}$ (15 µmol) at -40 °C. A solution of 5-(3-Cyano-5-(pyridin-2-ylethynyl)phenyl)-2,4-dimethoxy-8-methyldibenzo[b,d]thiophen-5-ium Trifluoromethanesulfonate (2.4 mg, 4.0 µmol) in MeCN (900 µL) was added to the second vessel and the mixture was heated at 80 °C for 5 min. After addition of water (2 mL), the crude product was purified by semi-preparative HPLC (Phenomenex Luna C_{18}(2) 5 µm 10 x 250 mm); H_2O/MeOH + 0.5% TFA 50:50; 4.0 mL/minute). The product in the isolated fraction was obtained with a radiochemical purity greater than 98% (Chromolith Performance C_{18} RP endcapped 5 µm 4.6 x 100 mm; H_2O + 0.5% TFA (A) and MeOH + 0.5% TFA (B); 0-3 min at 5% B, 3-8 min to 51% B, 8-10 min to 90% B, 10-11 min at 90% B, 11-15 min to 5% B, 15-17 min at 5% B; 3.0 mL/minute). The radiochemical yield (corrected to the start of synthesis) was 68 ± 3% and the molar activity at the end of synthesis was 5.7 ± 1 GBq/µmol (*n* = 3).

3. Organic chemistry

3.1 Synthesis and characterization of arylstannane precursors

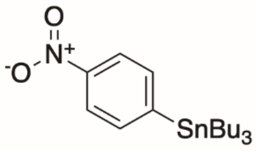

Tributyl(4-nitrophenyl)stannane (1): 1-iodo-4-nitrobenzene (200 mg, 0.803 mmol) and $Pd_2(dba)_3$ (23 mg, 0.025 mmol) were dissolved in 2-propanol (10 mL) under argon. *N,N*-Diisopropylethylamine (0.44 mL, 2.5 mmol) and hexa-*n*-butylditin (0.56 mL, 1.1 mmol) were subsequently added. The reaction mixture was stirred at room temperature overnight. After TLC indicated complete conversion, the reaction mixture was filtered through a plug of Celite and the filtrate concentrated *in vacuo*. The crude product was purified by flash chromatography on silica gel using hexane/DCM/Et_3N 94:5:1 as eluent. The final product was obtained as a pale yellow oil (173 mg, 0.420 mmol, 52%). R_f = 0.30 (hexane); ^{1}H NMR (500 MHz, $CDCl_3$): δ = 8.13 (d, *J* = 8.5 Hz, 2H), 7.64 (d, *J* = 8.5 Hz, 2H), 1.62 – 1.44 (m, 6H), 1.33 (h, *J* = 7.3 Hz, 6H), 1.20 – 1.04 (m, 6H), 0.89 (t, *J* = 7.3 Hz, 9H); ^{13}C NMR (126 MHz, $CDCl_3$): δ = 137.2, 132.2, 128.7, 122.1, 29.1, 27.4, 13.8, 10.0.

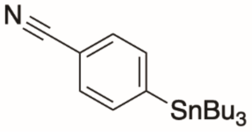

4-(tributylstannyl)benzonitrile (2): 4-iodobenzonitrile (200 mg, 0.873 mmol) and $Pd_2(dba)_3$ (23 mg, 0.025 mmol) were dissolved in 2-propanol (10 mL) under argon. *N,N*-Diisopropylethylamine (0.44 mL, 2.5 mmol) and hexa-*n*-butylditin (0.56 mL, 1.1 mmol) were subsequently added. The reaction mixture was stirred at room temperature overnight. After TLC indicated complete conversion, the reaction mixture was filtered through a plug of Celite and the filtrate concentrated *in vacuo*. The crude product was purified by flash chromatography on silica gel using PE/DCM 9:1 as eluent. The final product was obtained as a colorless oil (255 mg, 0.650 mmol, 74). R_f = 0.25 (hexane); ^{1}H NMR (500 MHz, $CDCl_3$): δ = 7.62 – 7.52 (m, 4H), 1.58 – 1.46 (m, 6H), 1.32 (h, *J* = 7.3 Hz, 6H), 1.16 – 1.01 (m, 6H), 0.88 (t, *J* = 7.3 Hz, 9H); ^{13}C NMR (126 MHz, $CDCl_3$): δ = 150.5, 137.0, 130.9, 119.4, 111.7, 29.1, 27.4, 13.8, 9.9; HRMS (ESI): *m/z* calcd for $C_{19}H_{31}NSn$ $[M+H]^+$: 394.1563, found: 394.1551.

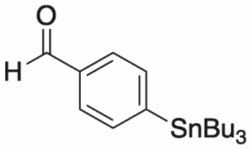

4-(tributylstannyl)benzaldehyde (3): To a solution of 4-bromobenzaldehyde (200 mg, 1.08 mmol) in toluene (10 mL) was added $Pd(PPh_3)_4$ (62 mg, 0.054 mmol). Hexa-*n*-butylditin (1.1 mL, 2.2 mmol) was then added under argon. The reaction mixture was stirred at 100 °C overnight. After TLC indicated complete conversion, the reaction mixture was cooled to room temperature, filtered through a plug of Celite and the filtrate concentrated *in vacuo*. The crude product was purified by flash chromatography on silica gel using a gradient of hexane/DCM 1:0 to 7:3 as eluent. The final product was obtained as a colorless oil (230 mg, 0.582 mmol, 54%). R_f = 0.50 (hexane/EtOAc 9:1); ^{1}H NMR (500 MHz, $CDCl_3$): δ = 9.99 (s, 1H), 7.79 (d, *J* = 8.0 Hz, 2H), 7.65 (d, *J* = 7.9 Hz, 2H), 1.57 – 1.50 (m, 6H), 1.33 (h, *J* = 7.3 Hz, 6H), 1.11 – 1.08 (m, 6H), 0.88 (t, *J* = 7.3 Hz, 9H); ^{13}C NMR (126 MHz, $CDCl_3$): δ = 192.92, 152.67, 137.02, 135.99, 128.52, 29.08, 27.36, 13.68, 9.79; HRMS (ESI): *m/z* calcd for $C_{18}H_{32}OSn$ $[M+H]^+$ 397.1570, found 397.1548.

3.2 Synthesis and characterization of aryl iodonium ylide precursors

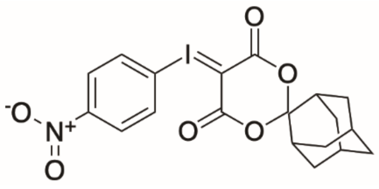

5'-((4-nitrophenyl)-l3-iodanylidene)spiro[adamantane-2,2'-[1,3]dioxane]-4',6'-dione (4): To a solution of 1-iodo-4-nitrobenzene (249 mg, 1.00 mmol) in AcOH (6.6 mL) was added *m*CPBA (190 mg, 1.10 mmol). The reaction mixture was stirred at 55 °C until TLC indicated complete conversion. The reaction mixture was then poured into water (10 mL) and extracted with chloroform (3 × 10 mL). The combined organic layers were dried over anhydrous Na_2SO_4, filtered and dried *in vacuo*. To the residue (ca. 1.0 mL) were added Et_2O (7.0 mL) and hexane (14 mL) and the mixture was cooled to induce precipitation. The product was filtered, washed with a mixture of Et_2O and hexane and left to dry in a vacuum oven for 2 h. The crude aryliodonium(III) diacetate (0.42 mmol) was diluted with chloroform (4.6 mL). A solution of SPIAD (100 mg, 0.423 mmol) in 10% Na_2CO_3(aq) (*w/v*, 6.6 mL, 0.33 M) was added. The pH was adjusted to ~9 using 10% Na_2CO_3(aq) after which the reaction mixture was stirred at room temperature till TLC indicated complete conversion (generally 2 h). The reaction mixture was then several times extracted with water (8 x 10 mL). The organic layer was dried over anhydrous Na_2SO_4, filtered, washed with chloroform and dried *in vacuo*. The final product was obtained as a pale orange solid (193 mg, 0.399 mmol, 40%). ^{1}H NMR (500 MHz, DMSO-d_6): δ = 8.30 – 8.25 (m, 2H), 8.05 – 8.00 (m, 2H), 2.36 (s, 2H), 1.95 (d, J = 12.4 Hz, 4H), 1.80 (2, 2H), 1.75 – 1.64 (m, 6H); ^{13}C NMR (126 MHz, DMSO-d_6): δ = 162.6, 149.0, 133.7, 126.1, 123.2, 105.4, 36.5, 34.7, 33.6, 25.9; LC-MS: t_R = 4.09 min, purity = > 99%, m/z [M+H]$^+$ = 484.

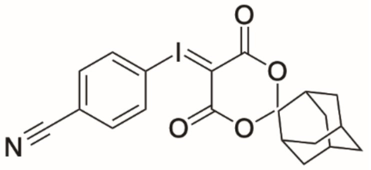

4-((4',6'-dioxospiro[adamantane-2,2'-[1,3]dioxan]-5'-ylidene)-l3-iodanyl)benzonitrile (5): To a solution of 4-iodobenzonitrile (229 mg, 1.00 mmol) in AcOH (6.6 mL) was added *m*CPBA (190 mg, 1.10 mmol). The reaction mixture was stirred at 55 °C until TLC indicated complete conversion. The reaction mixture was then poured into water (10 mL) and extracted with chloroform (3 × 10 mL). The combined organic layers were dried over anhydrous Na_2SO_4, filtered and dried *in vacuo*. To the residue (ca. 1.0 mL) were added Et_2O (7.0 mL) and hexane (7.0-14 mL) and the mixture was cooled to induce precipitation. The product was filtered, washed with a mixture of Et_2O/hexane 1:2 and left to dry in a vacuum oven for 2 h. The crude aryliodonium(III) diacetate (0.55 mmol) was diluted with ethanol (5.8 mL). A solution of SPIAD (129 mg, 0.546 mmol) in 10% Na_2CO_3(aq) (*w/v*, 8.3 mL, 0.33 M) was added. The pH was adjusted to ~9 using 10% Na_2CO_3(aq) after which the reaction mixture was stirred at rt till TLC indicated complete conversion (generally 2 h). The reaction mixture was then filtered and the filtrate left to dry in a vacuum oven overnight. The final product was obtained as a white solid (204 mg, 0.440 mmol, 44%). ^{1}H NMR (500 MHz, DMSO-d_6): δ = 7.91-7.93 (m, 4H), 2.36 (s, 2H), 1.94 (d, J = 12.4 Hz, 4H), 1.80 (s, 2H), 1.68 – 1.66 (m, 6H); ^{13}C NMR (126 MHz, DMSO-d_6): δ = 162.6, 134.4, 132.7, 120.2, 117.8, 114.0, 106.3, 36.1, 34.3, 33.2, 25.9; LC-MS: t_R = 3.92 min, purity = 98%, m/z [M+H]$^+$ = 464.

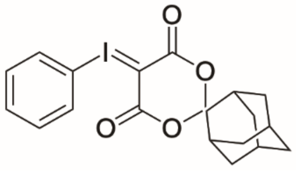

(1r,3r,5r,7r)-5'-(phenyl-l3-iodanylidene)spiro[adamantane-2,2'-[1,3]dioxane]-4',6'-dione (6): To a vigorously stirred suspension of $NaClO\cdot 5H_2O$ (331 mg, 2.01 mmol) in MeCN (1.50 mL) were added iodobenzene (223 µL, 2.00 mmol) and AcOH (343 µL, 6.00 mmol) at room temperature under air. The mixture was stirred for 10 min after which DCM (20 mL) was added. The reaction mixture was filtered and the solvents were removed under reduced pressure. The crude residue was added to NaOAc (328 mg, 4.00

mmol) and stirred in EtOAc (5.0 mL) at room temperature for 30 min. The reaction mixture was filtered, concentrated *in vacuo* and left to dry in a vacuum oven for 2 h. The crude (diacetoxyiodo)benzene (1.36 mmol) was diluted with ethanol (14.3 mL) and a solution of SPIAD (321 mg, 1.36 mmol) in 10% Na_2CO_3(aq) (*w/v*, 20.5 mL, 0.33 M) was added. The pH was adjusted to ~9 using 10% Na_2CO_3(aq) after which the reaction mixture was stirred at rt till TLC indicated complete conversion. The reaction mixture was then filtered and the filtrate left to dry in a vacuum oven overnight. The final product was obtained as a white fluffy solid (421 mg, 0.961 mmol, 48%). ^{1}H NMR (500 MHz, DMSO-d_6): δ = 7.81 – 7.73 (m, 2H), 7.58 – 7.51 (m, 1H), 7.49 – 7.41 (m, 2H), 2.34 (s, 2H), 1.94 (d, *J* = 12.4 Hz, 4H), 1.79 (s, 2H), 1.69 – 1.63 (m, 6H); ^{13}C NMR (126 MHz, DMSO-d_6): δ = 163.2, 132.3, 131.0, 130.6, 116.2, 105.1, 36.5, 35.4, 33.2, 26.0; LC-MS: t_R = 3.93 min, purity = > 99%, *m/z* [*M*+H]$^+$ = 439.

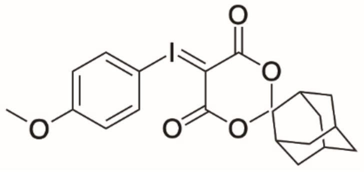

(1r,3r,5r,7r)-5'-((4-methoxyphenyl)-l3-iodanylidene)spiro[adamantane-2,2'-[1,3]dioxane]-4',6'-dione (7): To a solution of 1-iodo-4-methoxybenzene (234 mg, 1.00 mmol) in AcOH (6.6 mL) was added *m*CPBA (190 mg, 1.10 mmol). The reaction mixture was stirred at 55 °C until TLC indicated complete conversion. The reaction mixture was then poured into water (10 mL) and extracted with chloroform (3 × 10 mL). The combined organic layers were dried over anhydrous Na_2SO_4, filtered and dried *in vacuo*. To the residue (ca. 1.0 mL) were added Et_2O (7.0 mL) and hexane (7.0-14 mL) and the mixture was cooled to induce precipitation. The product was filtered, washed with a mixture of Et_2O and hexane and left to dry in a vacuum oven for 2 h. The crude aryliodonium(III) diacetate (0.58 mmol) was diluted with ethanol (6.1 mL). A solution of SPIAD (136 mg, 0.576 mmol) in 10% Na_2CO_3(aq) (*w/v*, 8.7 mL, 0.33 M) was added. The pH was adjusted to ~9 using 10% Na_2CO_3(aq) after which the reaction mixture was stirred at rt till TLC indicated complete conversion (generally 2 h). The reaction mixture was then filtered and the filtrate left to dry in a vacuum oven overnight. The final product was obtained as a white solid (150 mg, 0.320 mmol, 32%). ^{1}H NMR (500 MHz, DMSO-d_6): δ = 7.71 – 7.68 (m, 2H), 7.03 – 7.00 (m, 2H), 3.78 (s, 3H), 2.31 (s, 2H), 1.92 (d, *J* = 12.4 Hz, 4H), 1.79 (s, 2H), 1.66 – 1.63 (m, 6H); ^{13}C NMR (126 MHz, DMSO-d_6): δ = 162.5, 161.5, 134.5, 116.6, 105.6, 104.9, 55.5, 36.5, 34.8, 33.2, 26.0; LC-MS: t_R = 4.02 min, purity = > 99%, *m/z* [*M*+H]$^+$ = 469.

3.3 Synthesis and characterization of SynVesT-1 precursor and reference

The non-radioactive reference ([^{19}F]SynVesT-1) as well as the precursor to [^{18}F]SynVesT-1 were prepared according to literature procedures.[3]

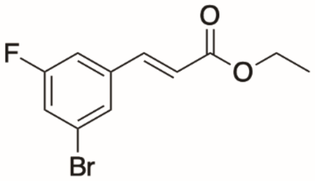

Ethyl-(E)-3-(3-bromo-5-fluorophenyl)acrylate (8): White solid (2.40 g, 8.79 mmol, 90%). R_f = 0.53 (hexane/EtOAc 9:1); ^{1}H NMR (500 MHz, $CDCl_3$): δ = 7.55 (d, *J* = 16 Hz, 1H), 7.45 (t, *J* = 1.8 Hz, 1H), 7.29 – 7.23 (m, 1H), 7.15 (dt, *J* = 9.3, 2.0 Hz, 1H), 6.42 (d, *J* = 16 Hz, 1H), 4.27 (q, *J* = 7.1 Hz, 2H), 1.34 (t, *J* = 7.1 Hz, 3H); ^{13}C NMR (126 MHz, $CDCl_3$): δ = 166.3, 162.9 (d, *J* = 251 Hz, 1C, C–F coupling), 141.7 (d, *J* = 2.6 Hz, 1C, C–F coupling), 138.1 (d, *J* = 8.3 Hz, 1C, C–F coupling), 127.1 (d, *J* = 3.1 Hz, 1C, C–F coupling), 123.3 (d, *J* = 9.9 Hz, 1C, C–F coupling), 121.3, 120.6 (d, *J* = 25 Hz, 1C, C–F coupling), 113.5 (d, *J* = 22 Hz, 1C, C–F coupling), 61.0, 14.4. Analysis is in line with the reported data.[3]

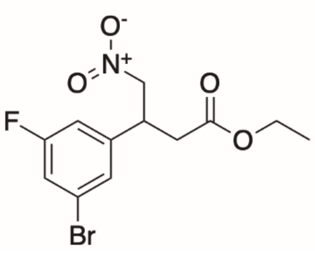

Ethyl 3-(3-bromo-5-fluorophenyl)-4-nitrobutanoate (9): Yellow oil (2.10 g, 6.29 mmol, 93%). R_f = 0.66 (hexane/EtOAc 9:1); ^{1}H NMR (500 MHz, $CDCl_3$): δ = 7.22 – 7.15 (m, 2H), 6.92 (dt, *J* = 9.0, 2.0 Hz, 1H), 4.72 (dd, *J* = 13, 6.6 Hz, 1H), 4.62 (dd, *J* = 13, 8.2 Hz, 1H), 4.11 (q, *J* = 7.1, 2H), 3.96 (p, *J* = 7.3 Hz, 1H), 2.79 – 2.66 (m, 2H), 1.21 (t, *J* = 7.2 Hz, 3H); ^{13}C NMR (126 MHz, $CDCl_3$): δ = 170.1, 162.9 (d, *J* = 252 Hz, 1C, C–F coupling), 142.5 (d, *J* = 7.7 Hz, 1C, C–F coupling), 126.7 (d, *J* = 3.2 Hz, 1C, C–F coupling), 123.4 (d, *J* = 10 Hz, 1C, C–F coupling), 119.1 (d, *J* = 24 Hz, 1C, C–F coupling), 113.8 (d, *J* = 22 Hz, 1C, C–F coupling), 78.8, 61.3, 39.7 (d, *J* = 1.9 Hz, 1C, C–F coupling), 36.6, 14.2. Analysis is in line with the reported data.[3]

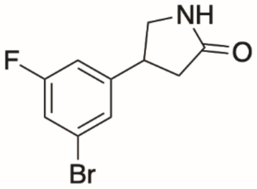

4-(3-bromo-5-fluorophenyl)pyrrolidin-2-one (10): Brownish oil (790 mg, 3.06 mmol, 52%). R_f = baseline (EtOAc); ^{1}H NMR (500 MHz, $CDCl_3$): δ = 7.19 (t, *J* = 1.6 Hz, 1H), 7.16 (dt, *J* = 8.0, 2.1 Hz, 1H), 6.91 (dt, *J* = 9.3, 2.0 Hz, 1H), 6.45 (s, 1H), 3.79 (t, *J* = 8.8 Hz, 1H), 3.66 (p, *J* = 8.2 Hz, 1H), 3.39 (dd, *J* = 9.7, 6.9 Hz, 1H), 2.74 (dd, *J* = 17, 9.0 Hz, 1H), 2.44 (dd, *J* = 17, 8.4 Hz, 1H); ^{13}C NMR (126 MHz, $CDCl_3$): δ = 177.0, 163.0 (d, *J* = 251 Hz, 1C, C–F coupling), 146.3 (d, *J* = 7.5 Hz, 1C, C–F coupling), 126.0 (d, *J* = 3.1 Hz, 1C, C–F coupling), 123.2 (d, *J* = 10 Hz, 1C, C–F coupling), 118.1 (d, *J* = 24 Hz, 1C, C–F coupling), 113.0 (d, *J* = 22 Hz, 1C, C–F coupling), 49.1, 39.8 (d, *J* = 1.9 Hz, 1C, C–F coupling), 37.6; LC-MS: t_R = 3.52 min, purity = 82%, *m/z* [*M*+H]$^+$ = 258 and 260. Analysis is in line with the reported data.[3]

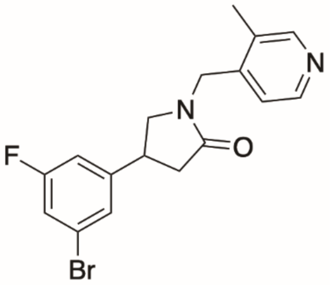

4-(3-bromo-5-fluorophenyl)-1-((3-methylpyridin-4-yl)methyl)-pyrrolidin-2-one (11): Brownish oil (690 mg, 1.90 mmol, 98%). R_f = 0.11 (EtOAc); ^{1}H NMR (500 MHz, $CDCl_3$): δ = 8.50 – 8.36 (m, 2H), 7.14 (dt, *J* = 7.9, 2.0 Hz, 1H), 7.11 (t, *J* = 1.7 Hz, 1H), 7.05 (d, *J* = 5.0 Hz, 1H), 6.83 (dt, *J* = 9.2, 2.0 Hz, 1H), 4.59 (d, *J* = 15 Hz, 1H), 4.43 (d, *J* = 16 Hz, 1H), 3.77 (dd, *J* = 9.2, 8.4 Hz, 1H), 3.71 (p, *J* = 8.4 Hz, 1H), 3.23 (dd, *J* = 9.3, 6.4 Hz, 1H), 2.91 (dd, *J* = 17, 8.8 Hz, 1H), 2.59 (dd, *J* = 17, 7.9 Hz, 1H), 2.31 (s, 3H); ^{13}C NMR (126 MHz, $CDCl_3$): δ = 173.2, 162.2 (d, *J* = 252 Hz, 1C, C–F coupling), 151.2, 147.8, 146.0 (d, *J* = 7.7 Hz, 1C, C–F coupling), 143.2, 131.9, 125.9 (d, *J* = 3.1 Hz, 1C, C–F coupling), 123.3 (d, *J* = 10 Hz, 1C, C–F coupling), 122.7, 118.2 (d, *J* = 24 Hz, 1C, C–F coupling), 113.0 (d, *J* = 22 Hz, 1C, C–F coupling), 53.6, 43.8, 38.2, 36.9 (d, *J* = 1.8 Hz, 1C, C–F coupling), 16.1; LC-MS: t_R = 2.95 min, purity = 87%, *m/z* [*M*+H]$^+$ = 363 and 365. Analysis is in line with the reported data.[3]

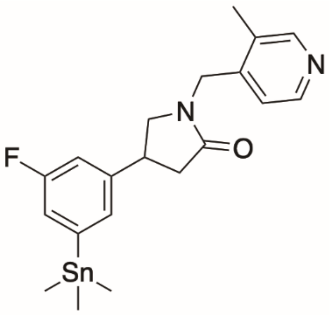

4-(3-fluoro-5-(trimethylstannyl)phenyl)-1-((3-methylpyridin-4-yl)methyl)pyrrolidin-2-one (12): White solid (170 mg, 0.380 mmol, 69%). R_f = 0.17 (EtOAc); ^{1}H NMR (500 MHz, $CDCl_3$): δ = 8.46 – 8.37 (m, 2H), 7.13 – 7.02 (m, 3H), 6.82 (dt, *J* = 9.9, 2.2 Hz, 1H), 4.64 (d, *J* = 16 Hz, 1H), 4.42 (d, *J* = 16 Hz, 1H), 3.67 – 3.56 (m, 2H), 3.28 (p, *J* = 7.1 Hz, 1H), 2.92 (dd, *J* = 17, 8.9 Hz, 1H), 2.66 (dd, *J* = 17, 8.4 Hz, 1H), 2.33 (s, 3H), 0.29 (s, 9H); ^{13}C NMR (126 MHz, $CDCl_3$): δ = 174.2, 163.8 (d, *J* = 252 Hz, 1C, C–F coupling), 151.2, 149.4, 146.8 (d, *J* = 5.6 Hz, 1C, C–F coupling), 143.8 (d, *J* = 5.6 Hz, 1C, C–F coupling), 143.4, 130.5, 130.0 (d, *J* = 2.8 Hz, 1C, C–F coupling), 121.3 (d,

J = 17 Hz, 1C, C–F coupling), 120.5, 113.6 (d, J = 22 Hz, 1C, C–F coupling), 54.5, 44.1, 39.0, 37.4 (d, J = 1.7 Hz, 1C, C–F coupling), 16.4, -9.0; LC-MS: t_R = 7.06 min, purity = 99%, m/z $[M+H]^+$ = 449. Analysis is in line with the reported data.[3]

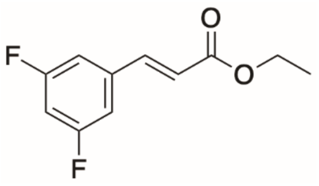

Ethyl (E)-3-(3,5-difluorophenyl)acrylate (13): White crystals (1.30 g, 6.27 mmol, 89%). R_f = 0.54 (hexane/EtOAc 9:1); ^{1}H NMR (500 MHz, $CDCl_3$): δ = 7.56 (d, J = 16 Hz, 1H), 7.02 (dt, J = 6.4, 2.2 Hz, 2H), 6.82 (tt, J = 8.7, 2.3 Hz, 1H), 6.42 (d, J = 16 Hz, 1H), 4.27 (q, J = 7.1 Hz, 2H), 1.34 (t, J = 7.2 Hz, 3H); ^{13}C NMR (126 MHz, $CDCl_3$): δ = 166.4, 163.3 (dd, J = 249, 12.5 Hz, 2C, C–F coupling), 142.1 (t, J = 2.9 Hz, 1C, C–F coupling), 137.8 (t, J = 9.5 Hz, 1C, C–F coupling), 121.2, 110.8 (dd, J = 26, 6.3 Hz, 2C, C–F coupling), 105.5 (t, J = 25 Hz, 1C, C–F coupling), 61.0, 14.4. Analysis is in line with the reported data.[3]

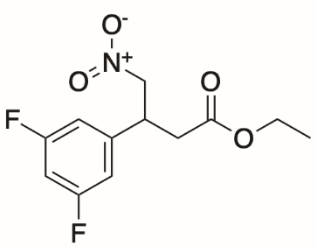

Ethyl 3-(3,5-difluorophenyl)-4-nitrobutanoate (14): Orange oil (1.65 g, 6.04 mmol, 99%). R_f = 0.65 (hexane/EtOAc 9:1); ^{1}H NMR (500 MHz, $CDCl_3$): δ = 6.80 – 6.72 (m, 3H), 4.72 (dd, J = 13, 6.5 Hz, 1H), 4.62 (dd, J = 13, 8.3 Hz, 1H), 4.11 (q, J = 7.2, 2H), 3.98 (p, J = 7.6 Hz, 1H), 2.73 (dd, J = 7.4, 4.3 Hz, 2H), 1.20 (t, J = 7.1 Hz, 3H); ^{13}C NMR (126 MHz, $CDCl_3$): δ = 170.3, 163.4 (dd, J = 250, 12.5 Hz, 2C, C–F coupling), 142.3 (t, J = 2.9 Hz, 1C, C–F coupling), 110.7 (dd, J = 20, 6.3 Hz, 2C, C–F coupling), 103.9 (t, J = 25 Hz, 1C, C–F coupling), 78.8, 61.4, 39.9 (t, J = 2.0 Hz, 1C, C–F coupling), 37.5, 14.2; HRMS (ESI): m/z calcd for $C_{12}H_{13}F_2NO_4$ $[M+Na]^+$: 296.0711, found: 296.0705. Analysis is in line with the reported data.[3]

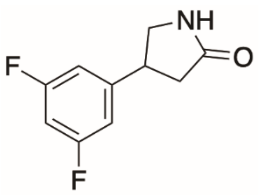

4-(3,5-difluorophenyl)pyrrolidin-2-one (15): Brownish oil (700 mg, 3.55 mmol, 59%). R_f = baseline (EtOAc); ^{1}H NMR (500 MHz, $CDCl_3$): δ = 6.78 – 6.73 (m, 2H), 6.71 (tt, J = 8.9, 2.4 Hz, 1H), 5.59 (s, 1H), 3.79 (t, J = 8.9 Hz, 1H), 3.67 (p, J = 8.2 Hz, 1H), 3.39 (dd, J = 9.7, 6.9 Hz, 1H), 2.75 (dd, J = 17, 9.0 Hz, 1H), 2.45 (dd, J = 17, 8.4 Hz, 1H); ^{13}C NMR (126 MHz, $CDCl_3$): δ = 177.5, 163.4 (dd, J = 249, 13.8 Hz, 2C, C–F coupling), 146.1 (t, J = 10 Hz, 1C, C–F coupling), 109.9 (dd, J = 25, 6.2 Hz, 2C, C–F coupling), 102.8 (t, J = 25 Hz, 1C, C–F coupling), 49.2, 39.9 (t, J = 2.0 Hz, 1C, C–F coupling), 37.7; LC-MS: t_R = 3.15 min, purity = 81%, m/z $[M+H]^+$ = 198. Analysis is in line with the reported data.[3]

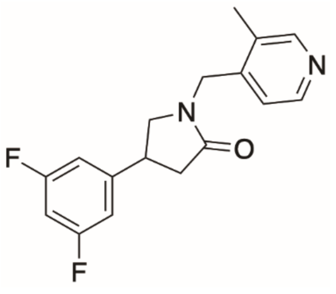

4-(3,5-difluorophenyl)-1-((3-methylpyridin-4-yl)methyl)-pyrrolidin-2-one (SynVesT-1): Yellow oil (1.10 g, 3.61 mmol, 96%). R_f = 0.11 (EtOAc); ^{1}H NMR (500 MHz, $CDCl_3$): δ = 8.46 – 8.44 (m, 2H), 7.06 (d, J = 5.0 Hz, 1H), 6.73 – 6.68 (m, 3H), 4.62 (d, J = 16 Hz, 1H), 4.41 (d, J = 16 Hz, 1H), 3.64 – 3.55 (m, 2H), 3.24 (dd, J = 6.1, 2.6 Hz, 1H), 2.91 (dd, J = 17, 8.8 Hz, 1H), 2.59 (dd, J = 17, 9.0 Hz, 1H), 2.31 (s, 3H); ^{13}C NMR (126 MHz, $CDCl_3$): δ = 173.3, 163.2 (dd, J = 250, 13.1 Hz, 2C, C–F coupling), 151.2, 147.8, 145.7 (t, J = 8.7 Hz, 1C, C–F coupling), 143.2, 132.0, 122.7, 109.8 (dd, J = 25, 14 Hz, 2C, C–F coupling), 102.9 (t, J = 25 Hz, 1C, C–F coupling), 53.5, 43.7, 38.1, 37.1 (t, J = 2.1 Hz, 1C, C–F coupling), 16.1; LC-MS: t_R = 3.03 min, purity = 96%, m/z $[M+H]^+$ = 303. Analysis is in line with the reported data.[3]

3.4 Characterization of *m*FBG and FPEB precursor and reference

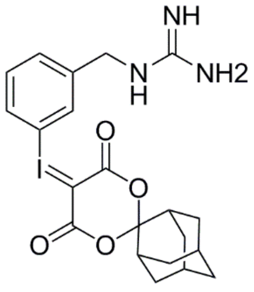

(1r,3r,5r,7r)-spiro[adamantane-2,2’-[1,3]dioxane]-4’,6’-dion-[3-((1,2,3,3-tetrakis(tert-butoxylcarbonyl)guanidino-3-iodonium)methyl)]ylide (16): ^{1}H NMR (500 MHz, MeCN-d_3): δ = 7.79 (s, 1H), 7.75 (d, *J* = 7.9 Hz, 1H), 7.58 (d, *J* = 7.9 Hz, 1H), 7.40 (t, *J* = 8.0 Hz, 1H), 4.98 (s, 2H), 2.37 (br., 2H), 2.15 (br., 2H), 2.03 (br., 2H), 2.00 (br., 2H), 1.82 (br., 2H), 1.74 (br., 4H), 1.71 (br., 2H), 1.44 (s, 9H), 1.42 (s, 18H), 1.40 (s, 9H); ^{13}C NMR (126 MHz, MeCN-d_3): δ = 163.8, 148.2, 146.1, 142.5, 132.9, 132.7, 132.3, 131.3, 115.7, 107.2, 85.5, 84.8, 82.9, 56.4, 50.7, 37.7, 36.5, 34.5, 28.2, 28.2, 28.1, 27.6; HRMS (ESI): *m/z* calcd for $C_{41}H_{56}IN_3O_{12}$ [*M*+Na]$^+$: 932.2807, found: 932.2887.

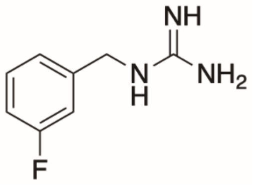

Meta-fluorobenzylguanidine (mFBG): ^{1}H NMR (500 MHz, D_2O): δ = 7.37 – 7.30 (m, 1H), 7.10 – 7.06 (m, 1H), 7.05 – 6.99 (m, 2H), 4.37 (s, 2H); ^{13}C NMR (126 MHz, D_2O): δ = 163.8, 162.7 (d, *J* = 244 Hz, 1H, C–F coupling), 138.7 (d, *J* = 6.3 Hz, 1H, C–F coupling), 130.6 (d, *J* = 8.8 Hz, 1H, C–F coupling), 122.4 (d, *J* = 2.5 Hz, 1H, C–F coupling), 114.5 (d, *J* = 23 Hz, 1H, C–F coupling), 113.5 (d, *J* = 21 Hz, 1H, C–F coupling), 43.9; HRMS (ESI): *m/z* calcd for $C_8H_{10}FN_3$ [*M*+H]$^+$: 168.0939, found: 168.0934.

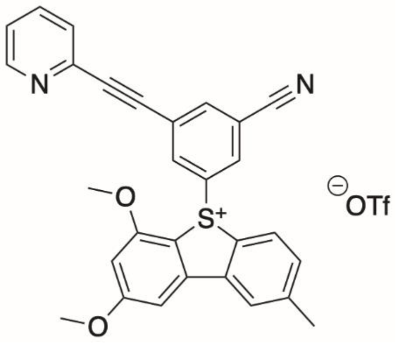

5-(3-Cyano-5-(pyridin-2-ylethynyl)phenyl)-2,4-dimethoxy-8-methyldibenzo[b,d]thiophen-5-ium trifluoromethanesul fonate (17): ^{1}H NMR (600 MHz, DMSO-d_6): δ = 8.64 (ddd, *J* = 4.8 Hz, 1.8 Hz, 1.0 Hz, 1H), 8.48 (t, *J* = 1.4 Hz, 1H), 8.41 (s, 1H), 8.28 (dd, *J* = 1.8 Hz, 1.4 Hz, 1H), 8.26 (d, *J* = 8.3 Hz, 1H), 8.14 (t, *J* = 1.8 Hz, 1H), 7.90 (td, *J* = 7.7 Hz, 1.8 Hz, 1H), 7.74 (d, *J* = 2.2 Hz, 1H), 7.69 (dt, *J* = 7.7 Hz, 1.0 Hz, 1H), 7.60 (d, *J* = 8.3 Hz, 1H), 7.49 (ddd, *J* = 7.7 Hz, 4.9 Hz, 1.0 Hz, 1H), 6.94 (d, *J* = 2.1 Hz, 1H), 4.01 (s, 3H), 3.95 (s, 3H), 2.55 (s, 3H); ^{13}C NMR (150 MHz, DMSO-d_6): δ = 167.2, 158.1, 150.5, 145.1, 142.5, 141.0, 140.5, 139.7, 137.1, 136.3, 133.6, 132.5, 130.6, 129.4, 128.2, 128.0, 125.8, 125.1, 124.6, 119.7 (q, *J* = 322 Hz), 116.1, 114.8, 107.3, 102.5, 100.8, 92.8, 84.2, 57.5, 56.8, 21.3; LC-MS: t_R = 3.58 min, purity = > 99%, *m/z* [*M*+H]$^+$ = 303.

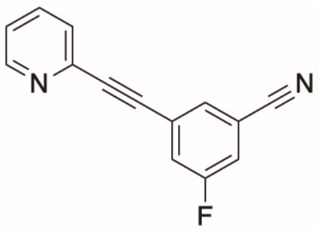

3-fluoro-5-(pyridin-2-ylethynyl)benzonitrile (FBEP): ^{1}H NMR (600 MHz, $CDCl_3$): δ = 8.66 (d, *J* = 4.9 Hz, 1H), 7.73 (td, *J* = 7.7, 1.8 Hz, 1H), 7.67 (br. s, 1H), 7.55 (dt, *J* = 7.8, 1.1 Hz, 1H), 7.52 (ddd, *J* = 8.7, 2.5, 1.4 Hz, 1H), 7.37 (ddd, *J* = 7.8, 2.5, 1.4 Hz, 1H), 7.32 (ddd, *J* = 7.7, 4.9, 1.2 Hz, 1H); ^{13}C NMR (150 MHz, $CDCl_3$): δ = 162.0 (d, *J* = 250 Hz), 150.5, 142.2, 136.6, 131.6 (d, *J* = 3.3 Hz), 127.7, 126.2 (d, *J* = 10 Hz), 123.9, 123.6 (d, *J* = 23 Hz), 119.6 (d, *J* = 25 Hz), 116.9 (d, *J* = 3.3 Hz), 114.5 (d, *J* = 10 Hz), 91.8, 85.2 (d, *J* = 3.5 Hz); LC-MS: t_R = 4.27 min, purity = >99%, *m/z* [*M*+H]$^+$ = 223.

4. HPLC chromatograms

Table S14 Overview of HPLC retention times; HPLC conditions: Phenomenex Luna C_{18} 5 µm 4.6 x 250 mm, MeCN/water 65:35, 1 mL/min.

References	t_R UV [min]	t_R RAD [min]
F^-	-	~2.3
1-fluoro-4-nitrobenzene	6.073	6.188
4-fluorobenzonitrile	5.412	5.508
4-fluorobenzaldehyde	4.681	4.784
fluorobenzene	7.129	7.226
1-fluoro-4-methoxybenzene	6.819	6.938

Example chromatograms: 1-[^{18}F]fluoro-4-methoxybenzene

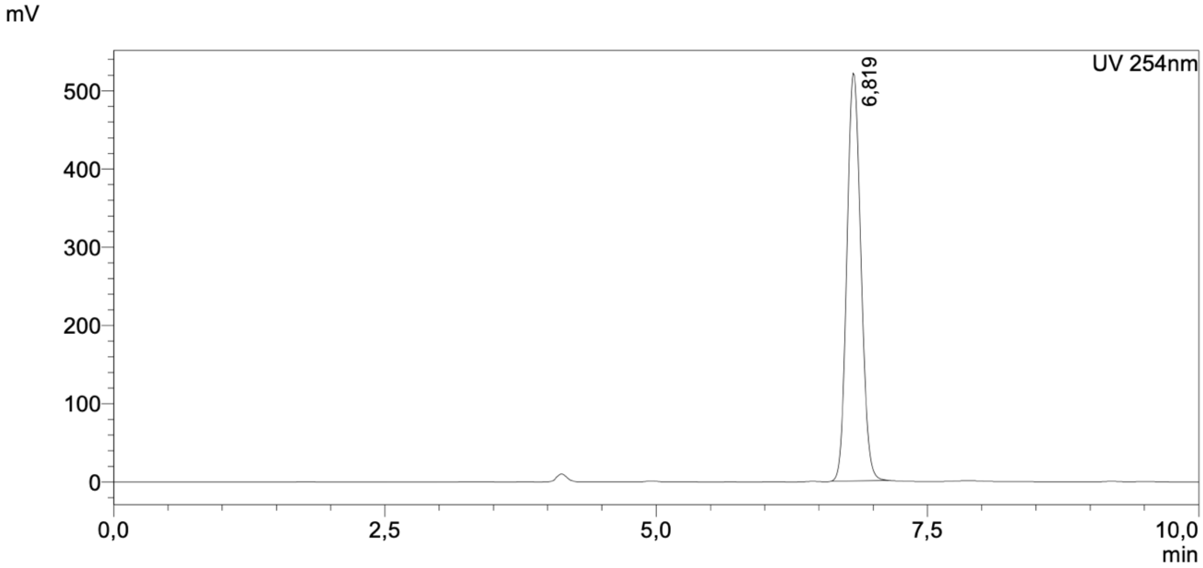

Figure S1 HPLC chromatogram of 1-fluoro-4-methoxybenzene (t_R = 6.819 min); Column: Phenomenex Luna C18 5 µm 4.6 x 250 mm, Eluent: 65:35 MeCN/H_2O, Flow rate: 1.0 mL/min.

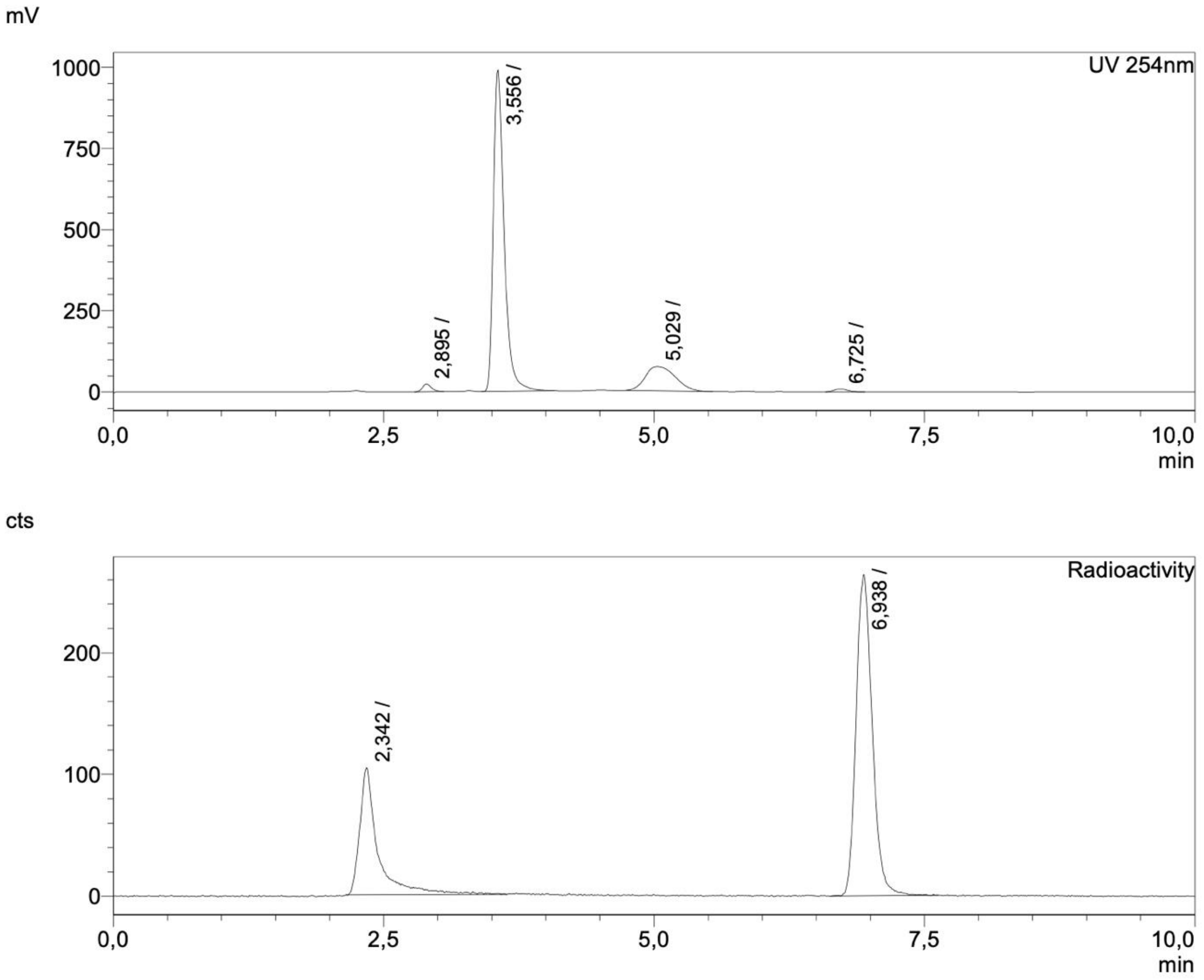

Figure S2 HPLC chromatogram of the **unoptimized** [^{18}F]fluorination reaction of 2-(4-methoxyphenyl)-4,4,5,5-tetramethyl-1,3,2-dioxaborolane (crude); top panel: UV at 254 nm; bottom panel: radioactivity signal (t_R [^{18}F]fluoride = 2.342 min, t_R 1-[^{18}F]fluoro-4-methoxybenzene = 6.938 min); Column: Phenomenex Luna C_{18} 5 µm 4.6 x 250 mm, Eluent: 65:35 MeCN/H_2O, Flow rate: 1.0 mL/min.

Chromatograms of [^{18}F]SynVesT-1, [^{18}F]mFBG and [^{18}F]FPEB

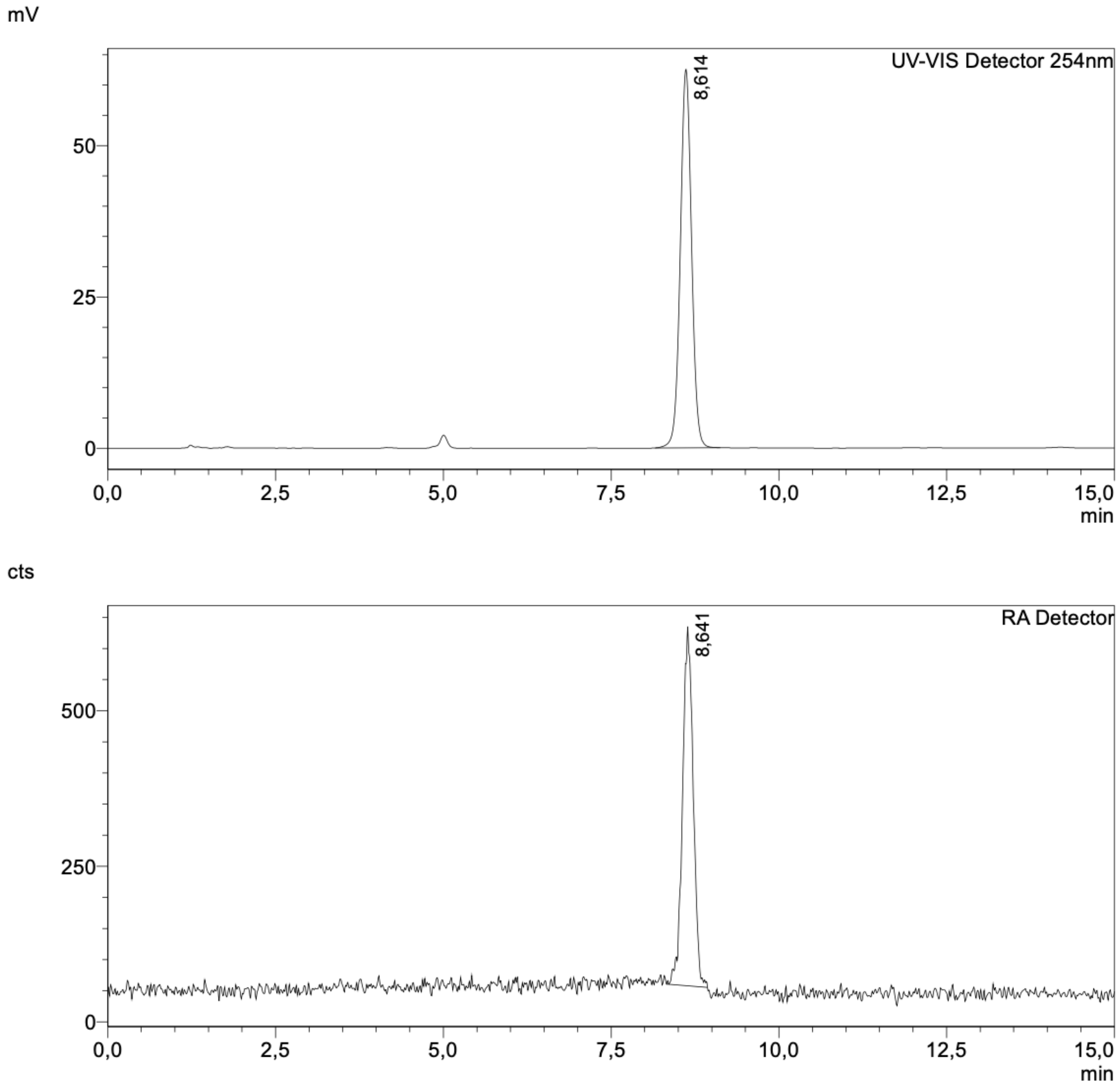

Figure S3 HPLC chromatogram of **pure** [^{18}F]SynVesT-1 synthesis + **spike with reference**; top panel: UV at 254 nm (t_R [^{19}F]SynVesT-1 = 8.614 min); bottom panel: radioactivity signal (t_R [^{18}F]SynVesT-1 = 8.641 min); Column: Phenomenex Luna C_{18} 5 µm 4.6 x 250 mm, Eluent: 30:70 MeCN/0.1 M ammonium formate buffer + 0.5% acetic acid (pH 4.2), Flow rate: 2.0 mL/min.

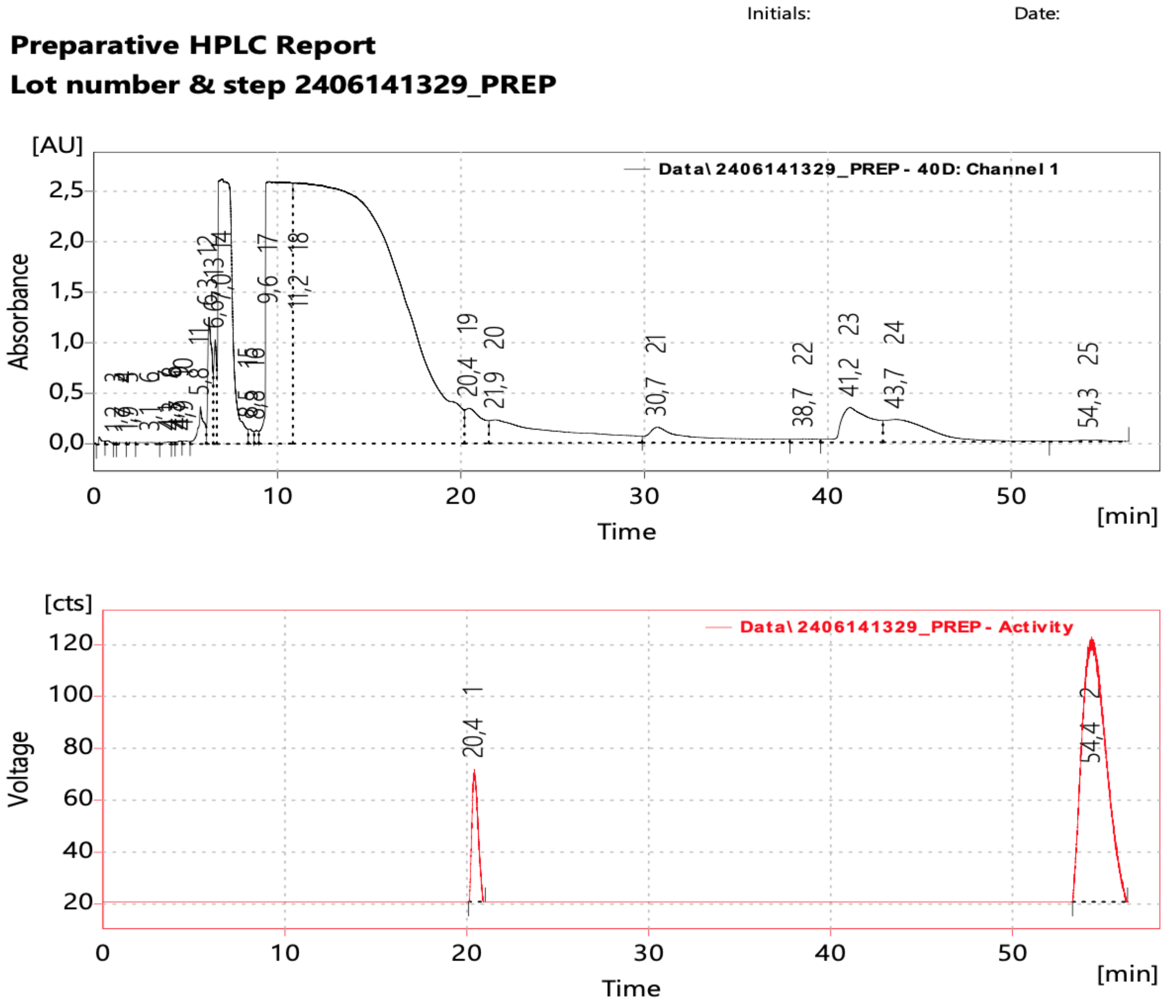

Figure S4 HPLC chromatogram of **optimized** [^{18}F]mFBG synthesis (**prep HPLC**); top panel: UV at 254 nm; bottom panel: radioactivity signal (t_R [^{18}F]fluoride = 20.41 min, t_R [^{18}F]mFBG = 54.42 min); Column: Alltima C_{18} 10 µm 22 x 250 mm, Eluent: H_2O/MeOH/HCl 89:10:1, Flow rate: 10.0 mL/minute.

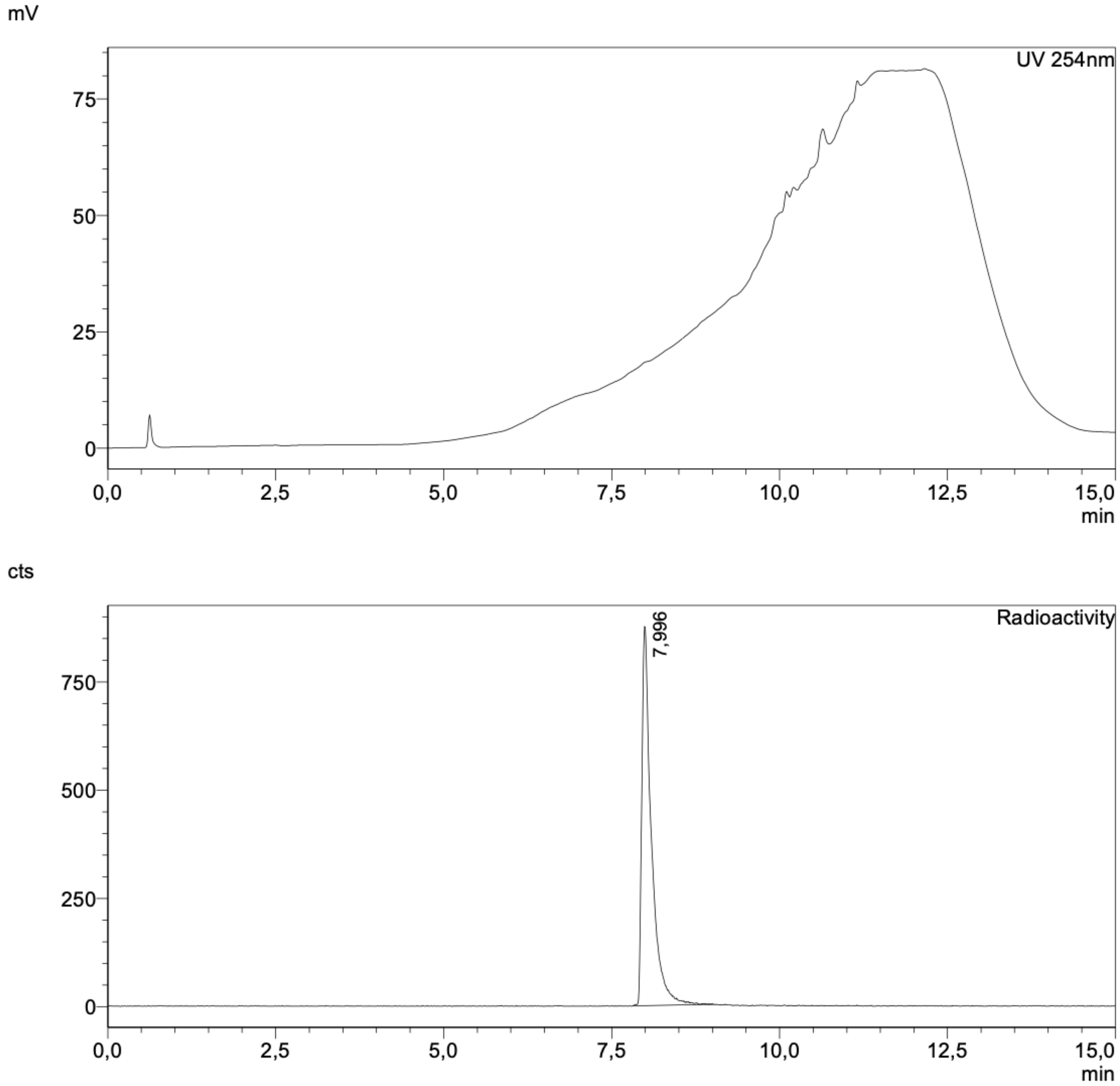

Figure S5 HPLC chromatogram of **pure** [^{18}F]FPEB synthesis; top panel: UV at 254 nm; bottom panel: radioactivity signal (t_R [^{18}F]FPEB = 7.996 min); Column: Chromolith Performance C_{18} RP endcapped 5 µm 4.6 x 100 mm, Eluent: H_2O + 0.5% TFA (A) and MeOH + 0.5% TFA (B); 0-3 min at 5% B, 3-8 min to 51% B, 8-10 min to 90% B, 10-11 min at 90% B, 11-15 min to 5% B, 15-17 min at 5% B, Flow rate: 3.0 mL/minute.

5. ^{1}H and ^{13}C NMR Spectra

Tributyl(4-nitrophenyl)stannane

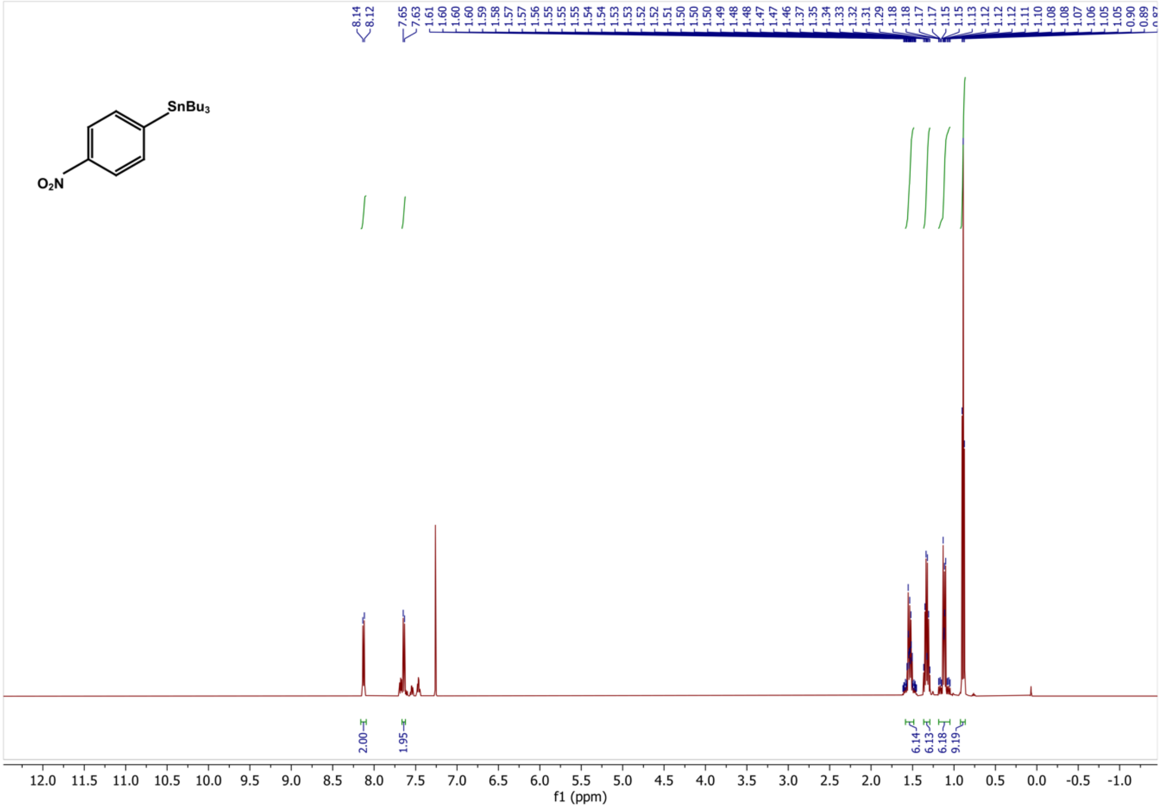

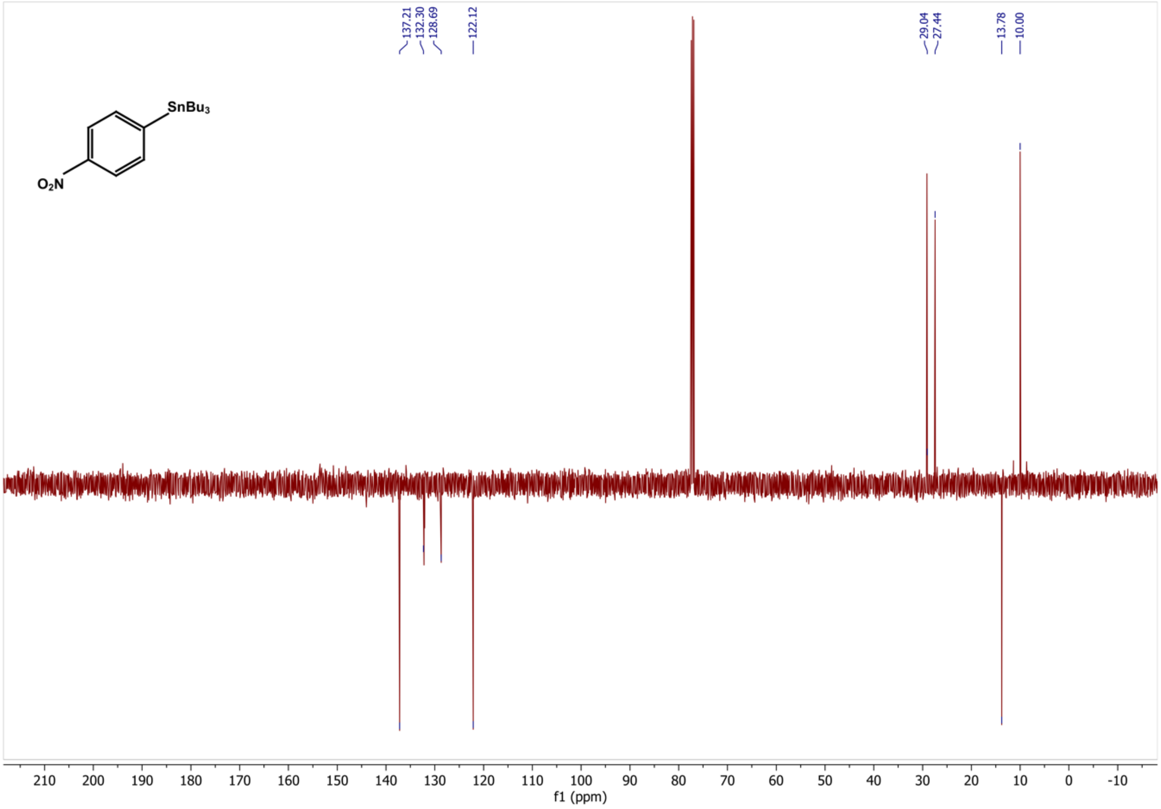

4-(tributylstannyl)benzonitrile

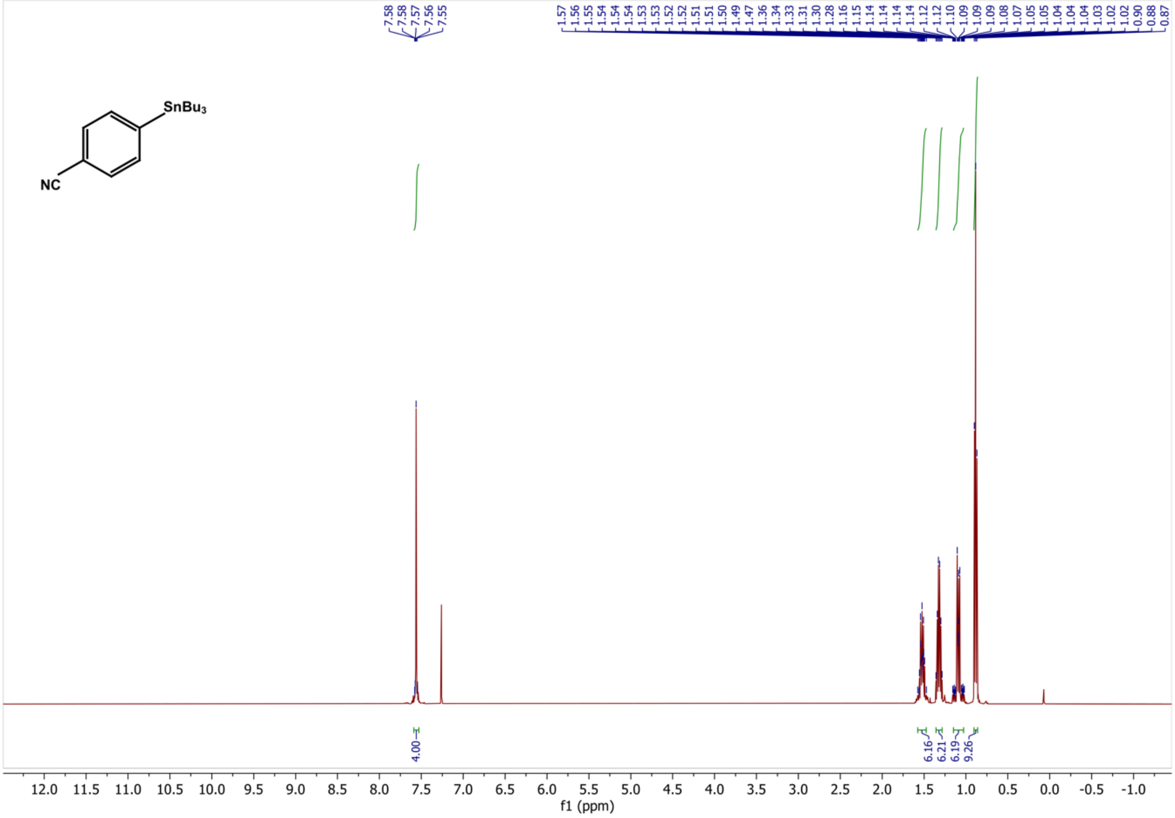

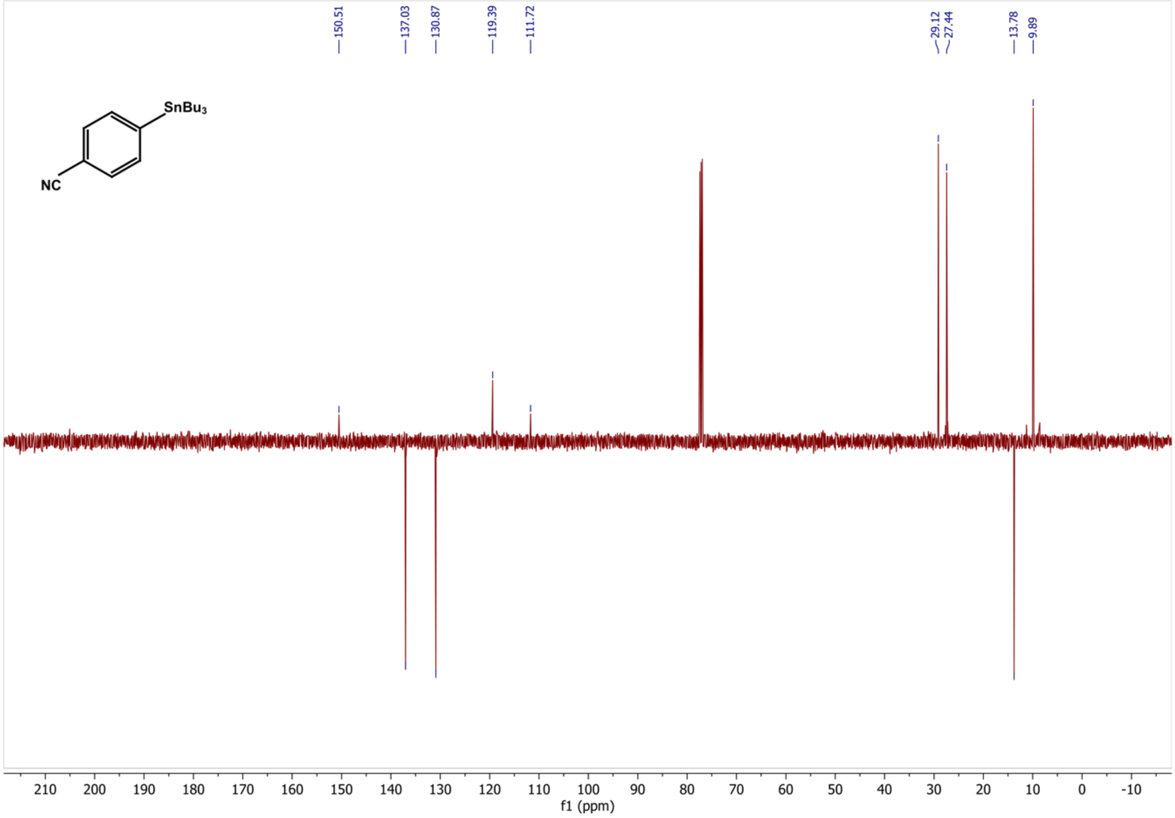

4-(tributylstannyl)benzaldehyde

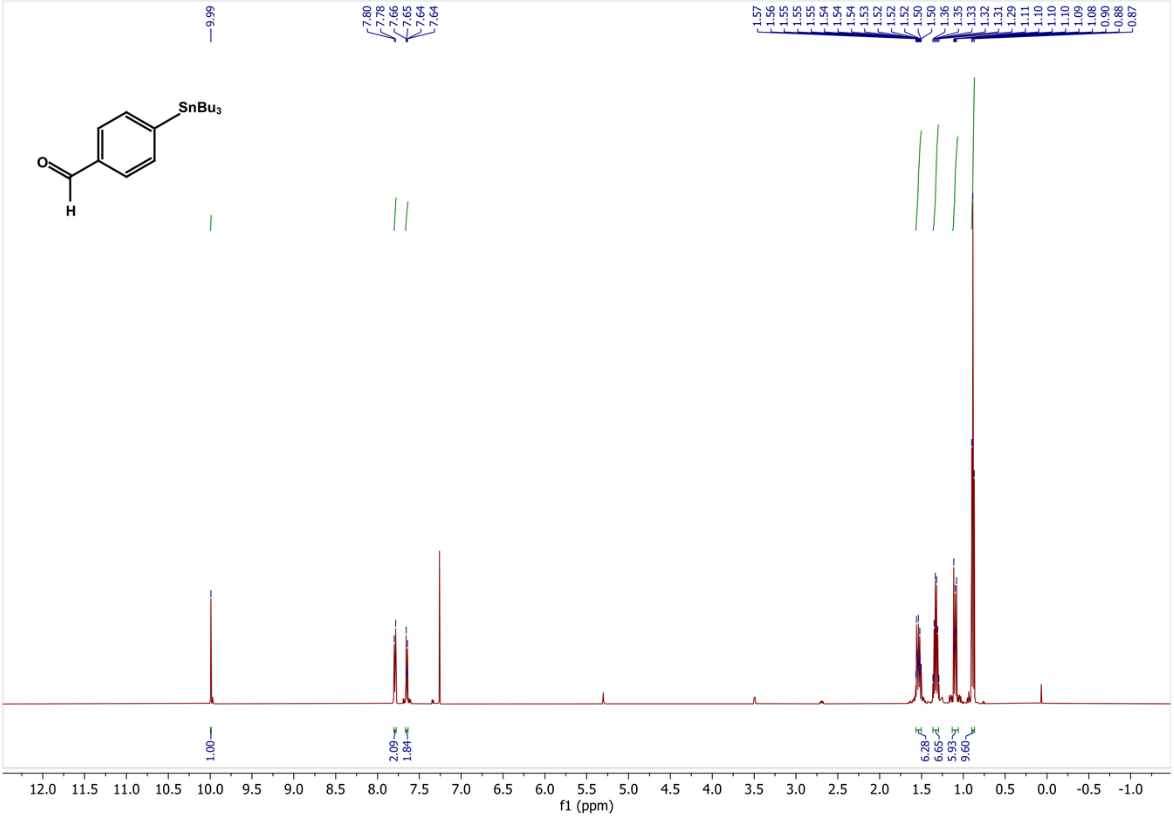

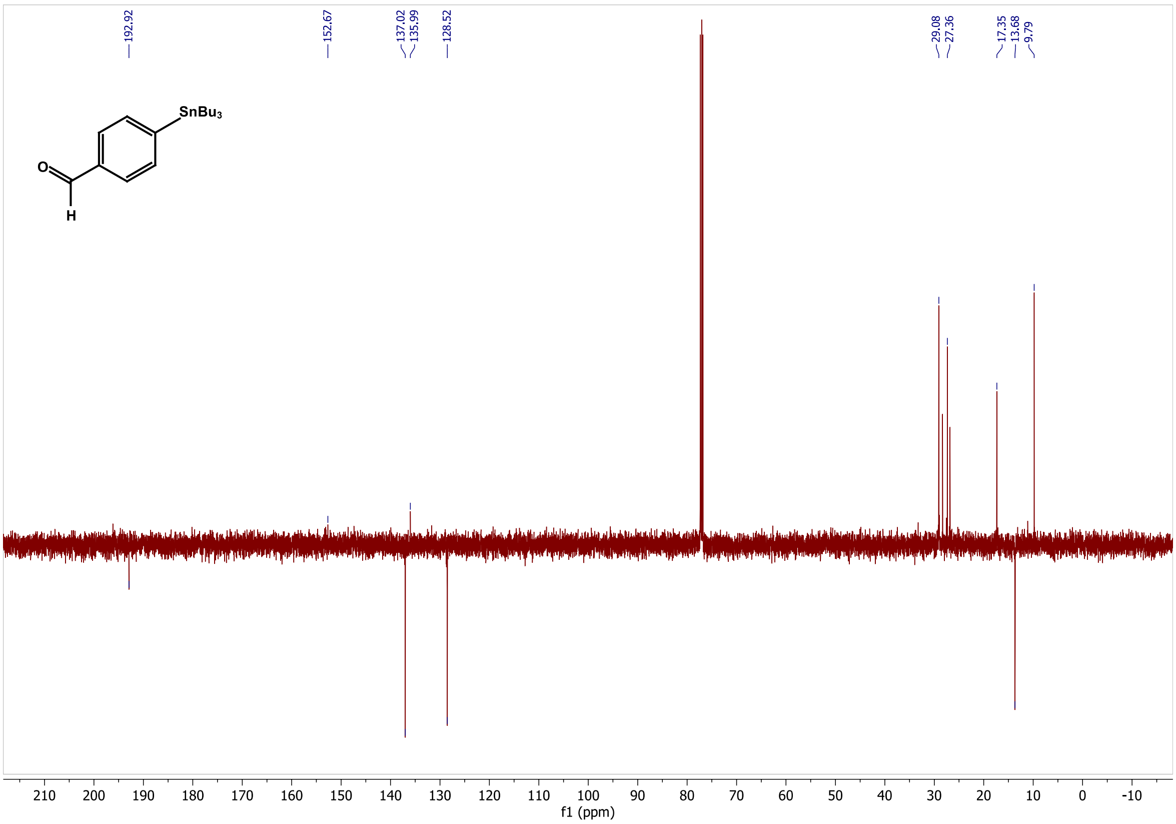

5'-((4-nitrophenyl)-l3-iodanylidene)spiro[adamantane-2,2'-[1,3]dioxane]-4',6'-dione

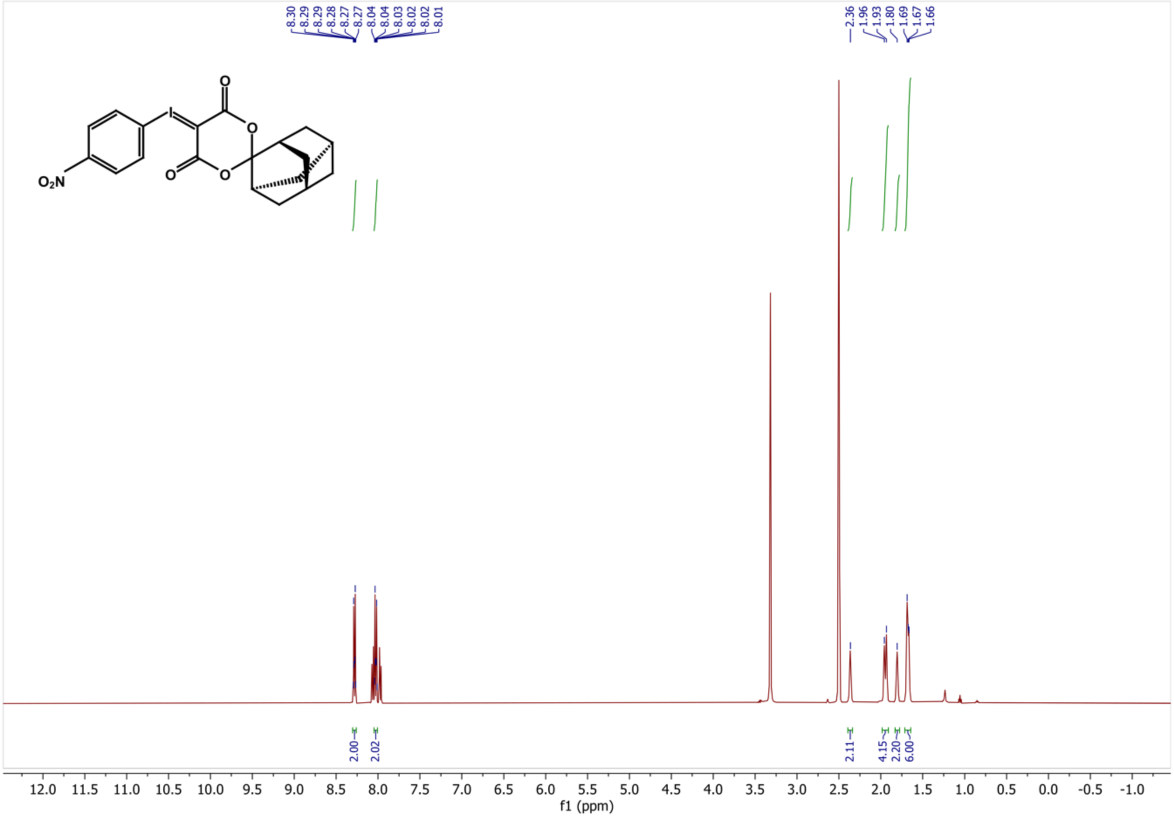

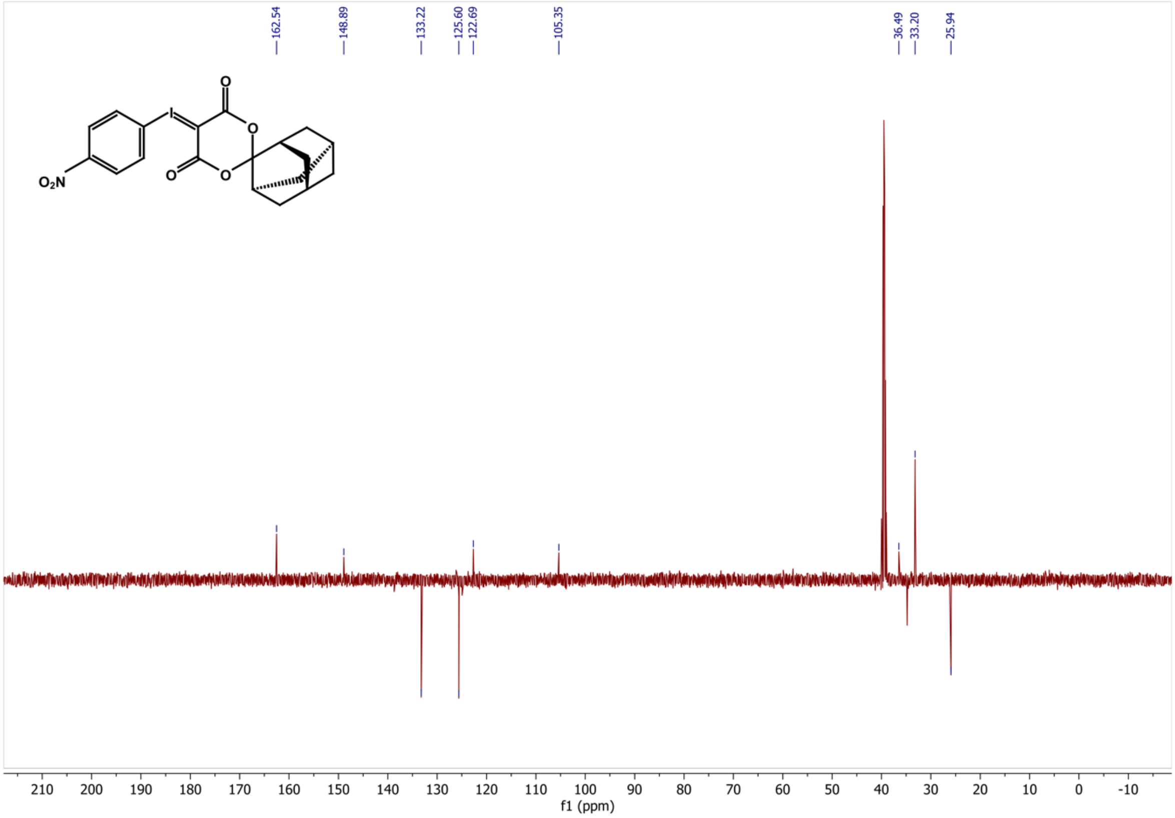

4-((4',6'-dioxospiro[adamantane-2,2'-[1,3]dioxan]-5'-ylidene)-I3-iodanyl)benzonitrile

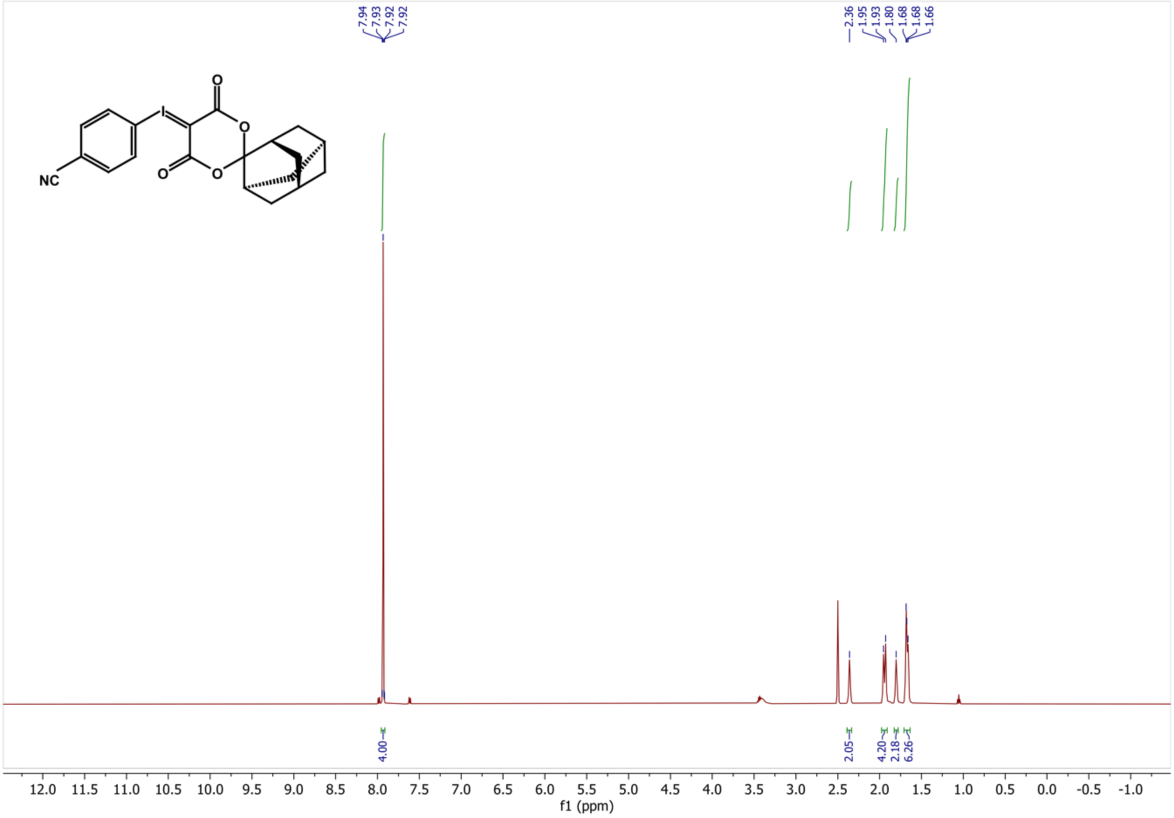

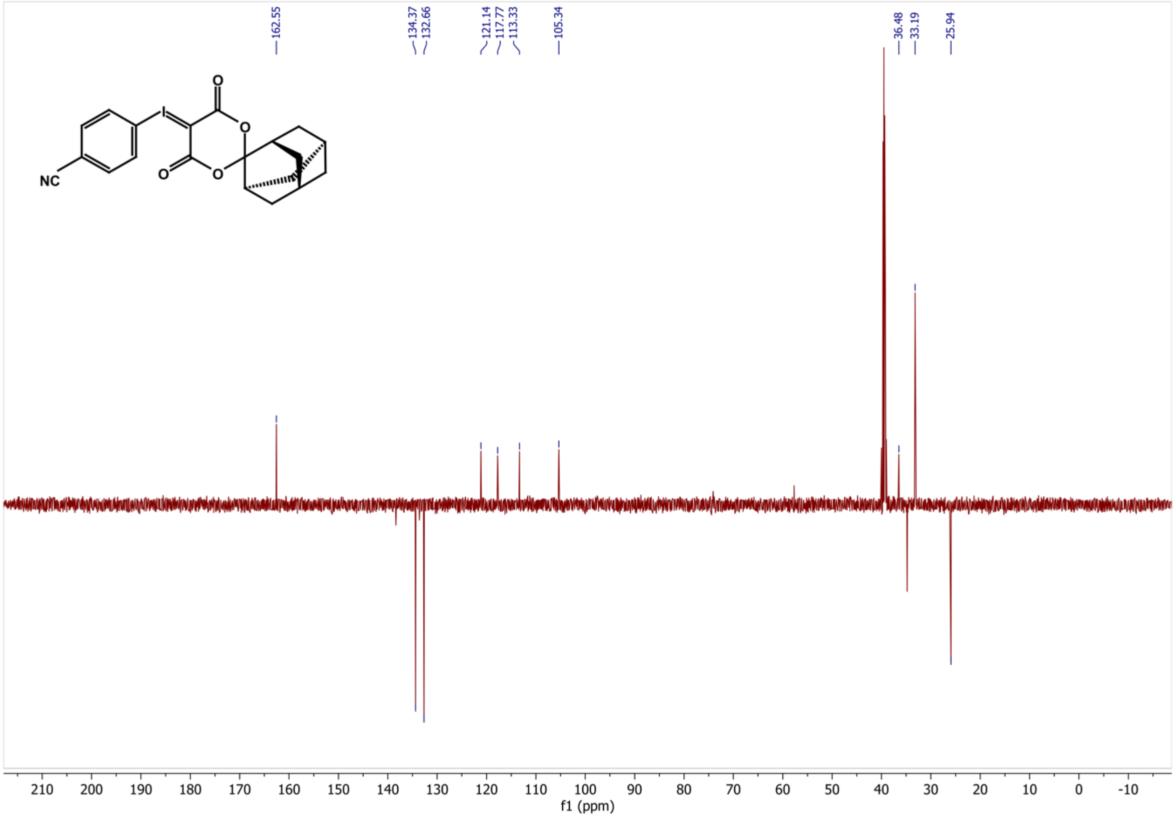

(1r,3r,5r,7r)-5'-(phenyl-l3-iodanylidene)spiro[adamantane-2,2'-[1,3]dioxane]-4',6'-dione

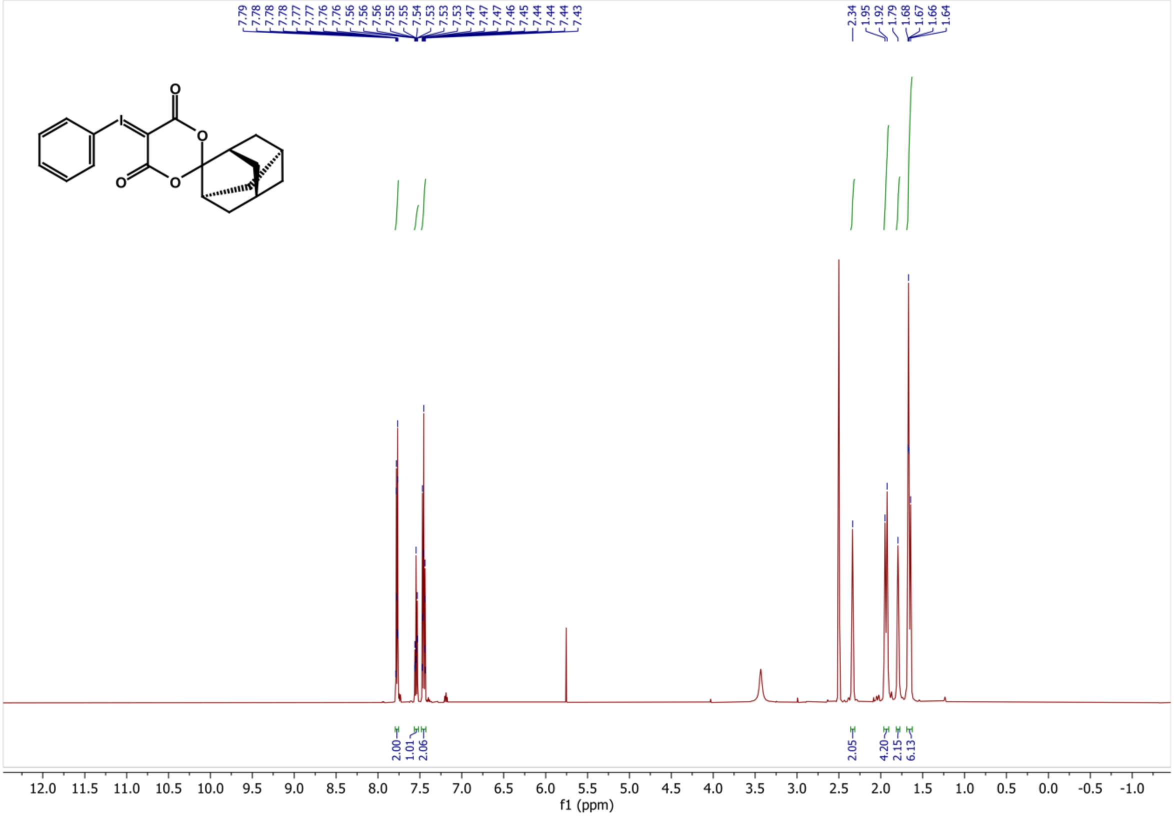

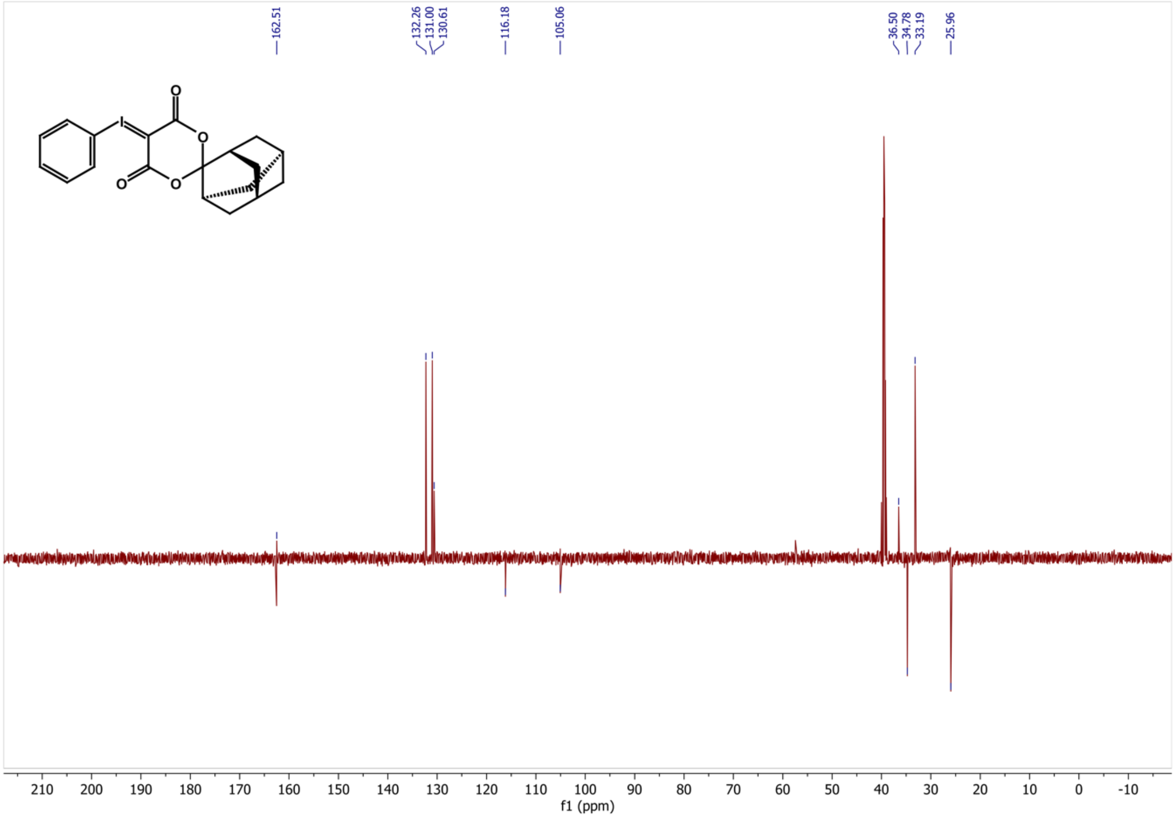

(1r,3r,5r,7r)-5'-((4-methoxyphenyl)-l3-iodanylidene)spiro[adamantane-2,2'-[1,3]dioxane]-4',6'-dione

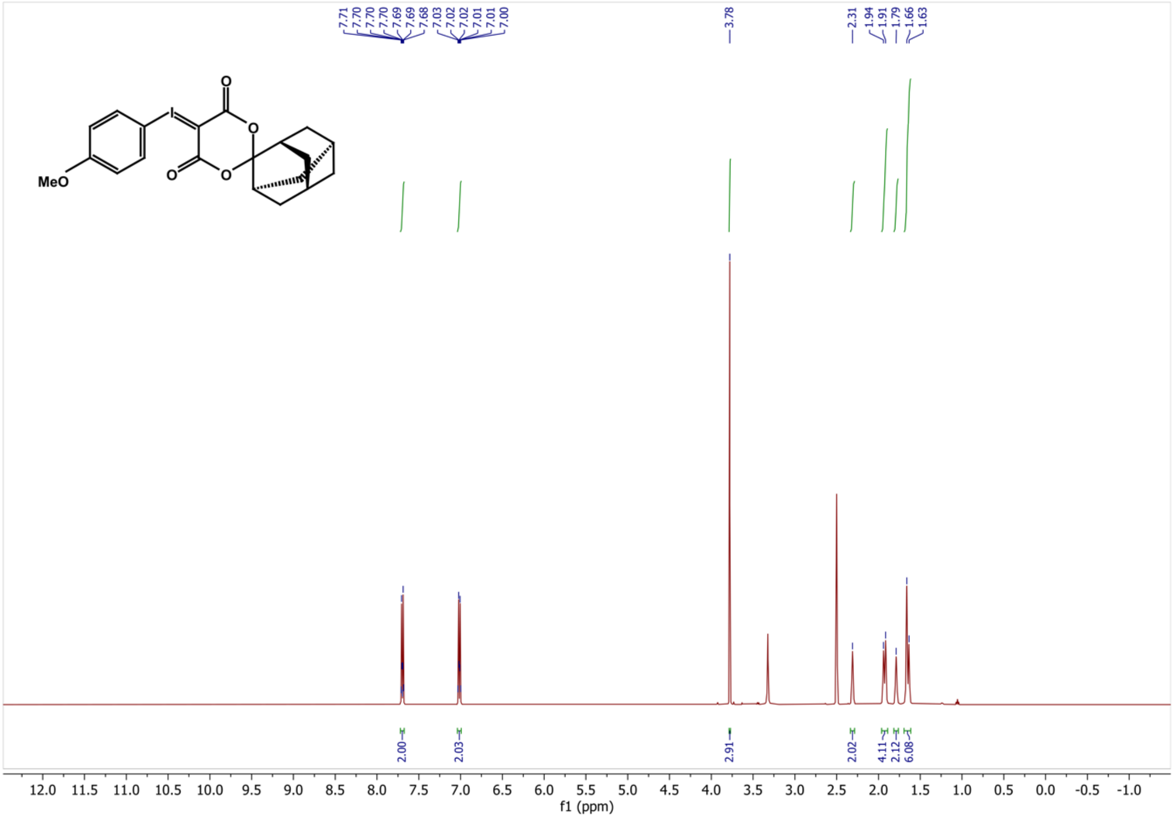

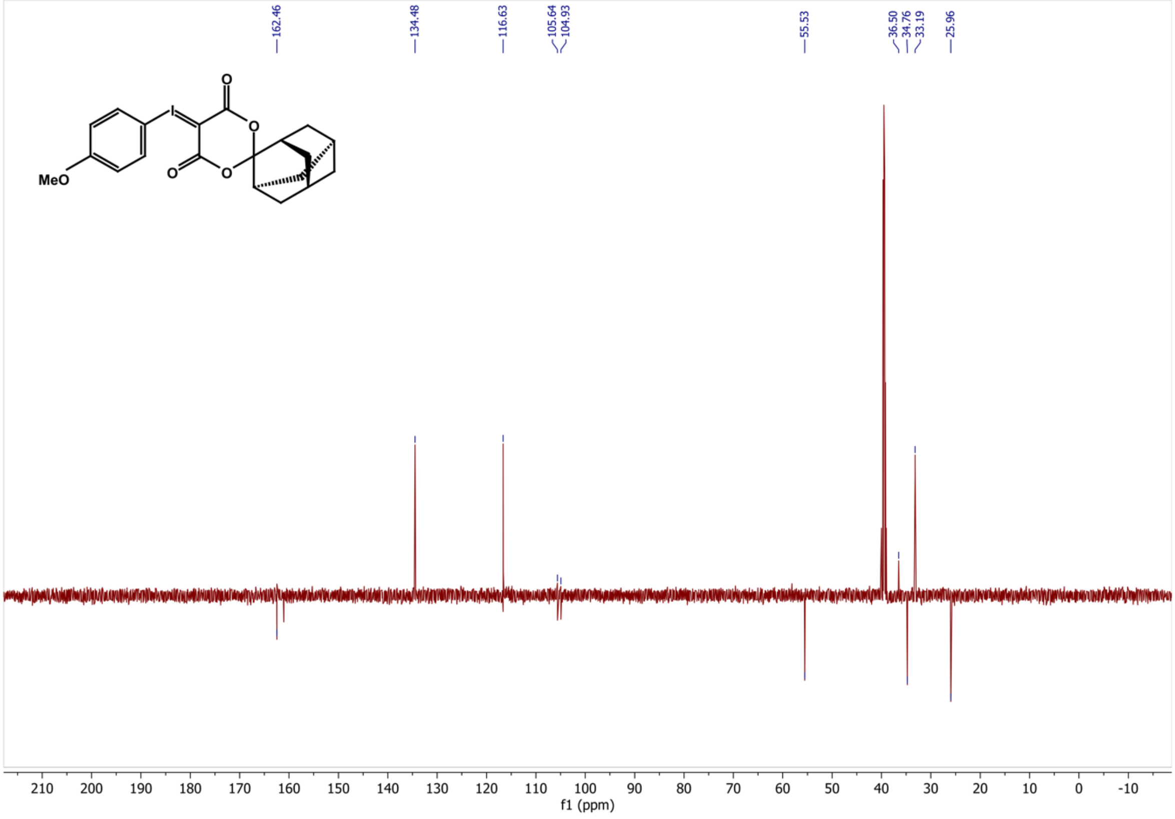

Ethyl-(E)-3-(3-bromo-5-fluorophenyl)acrylate

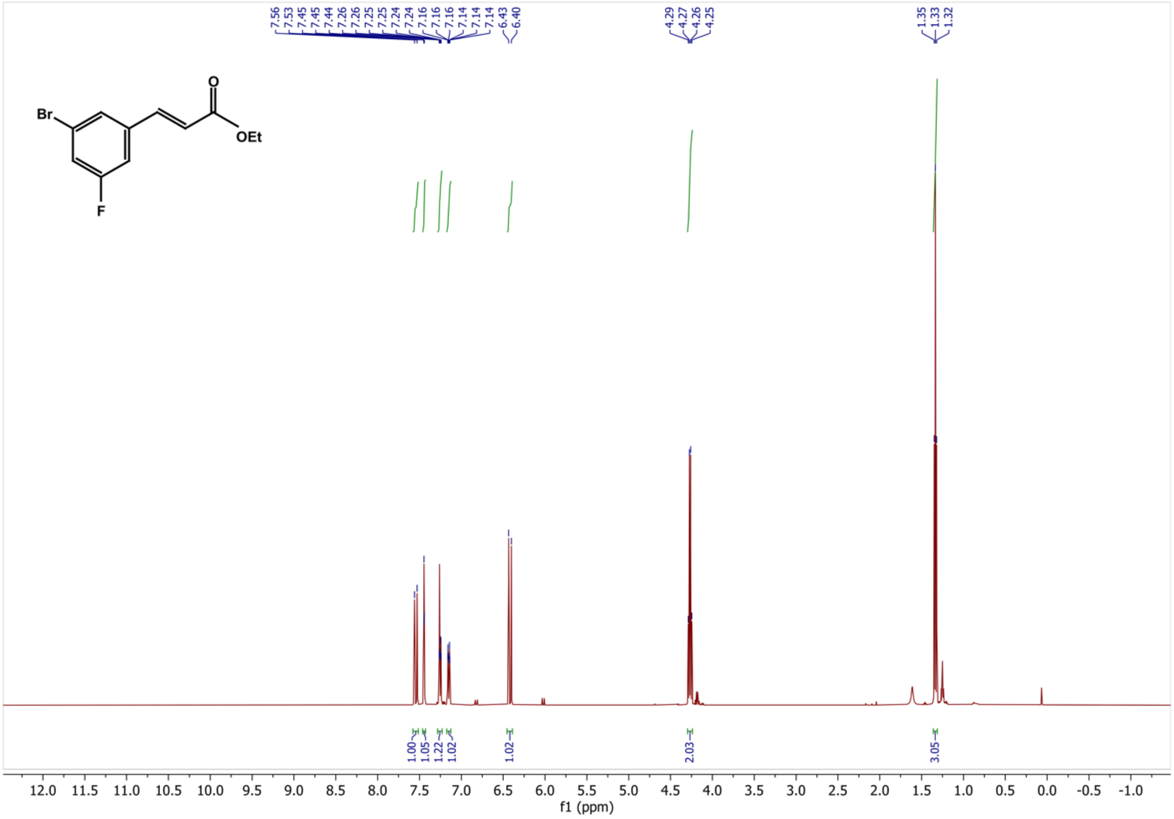

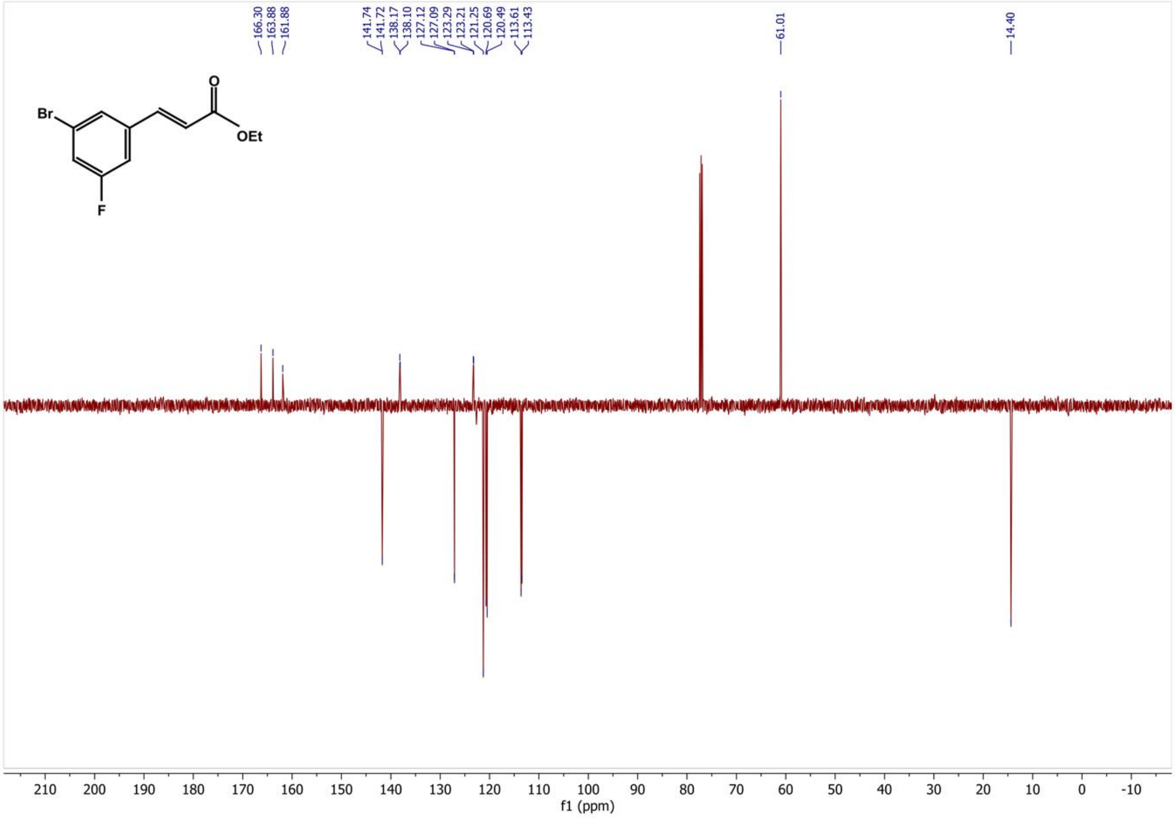

Ethyl 3-(3-bromo-5-fluorophenyl)-4-nitrobutanoate

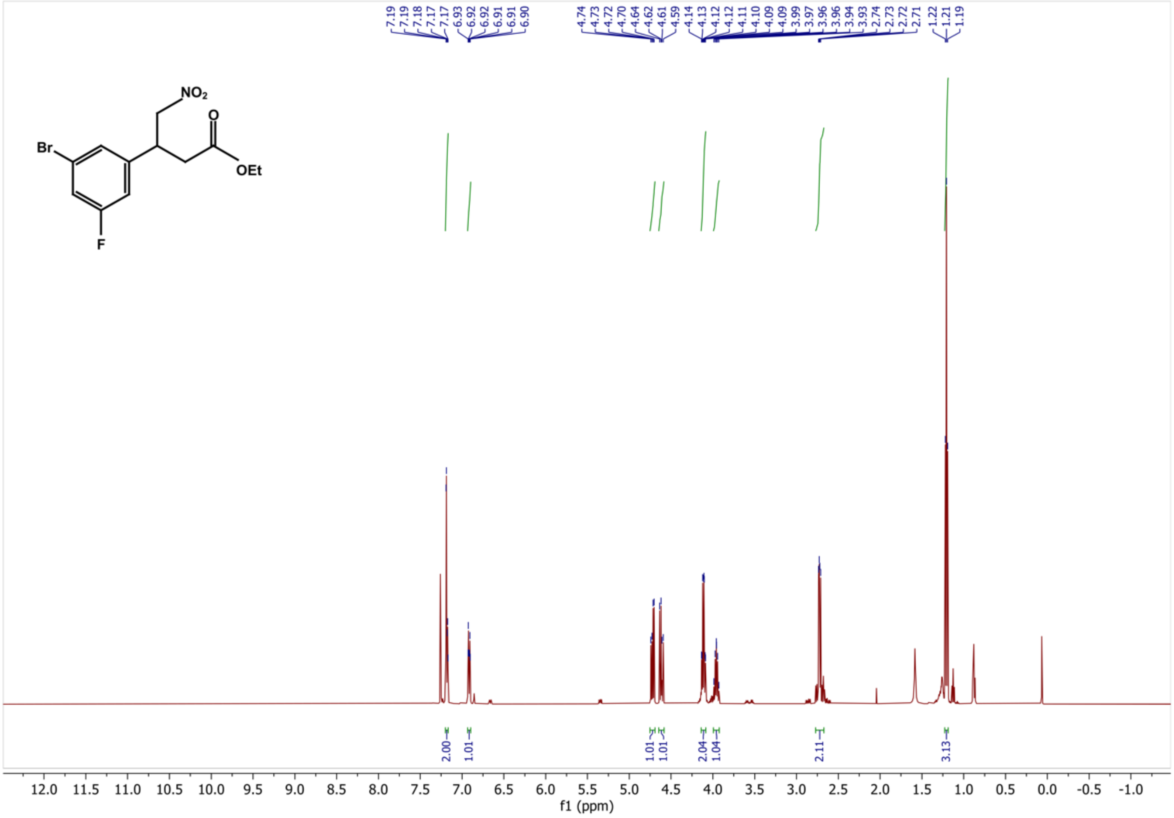

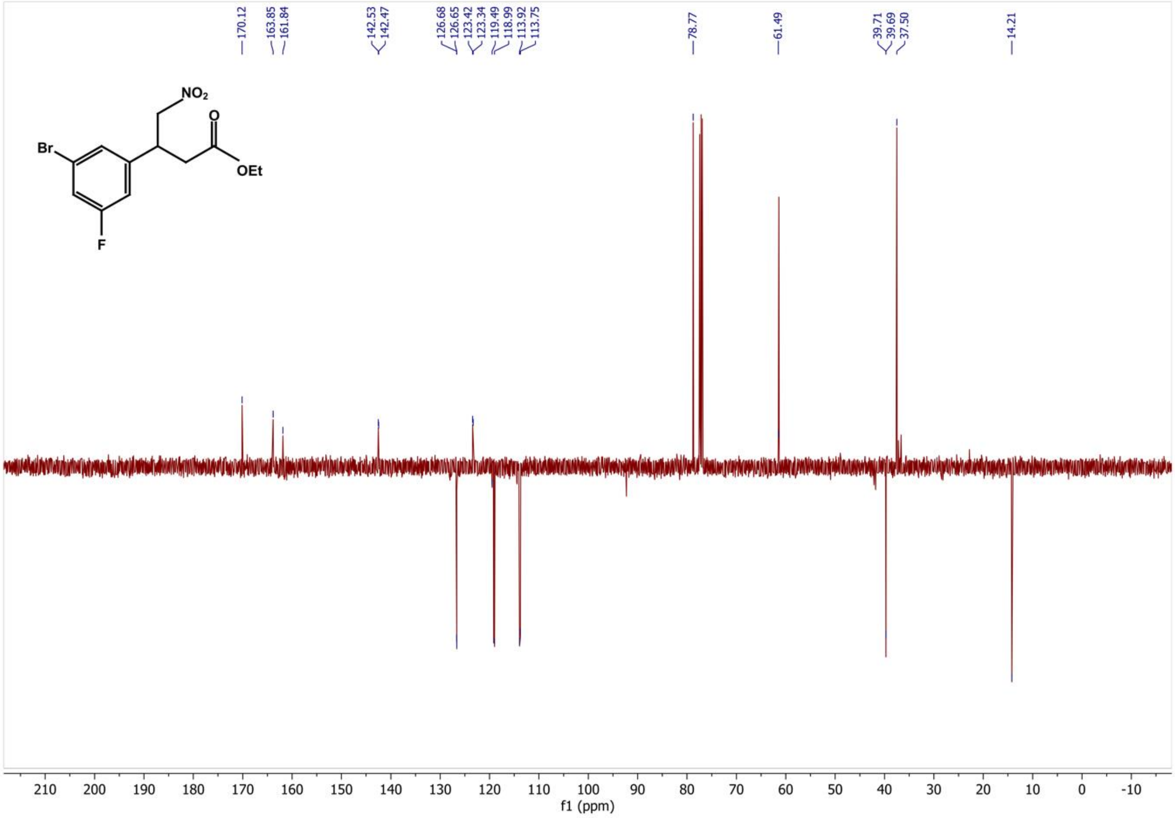

4-(3-bromo-5-fluorophenyl)pyrrolidin-2-one

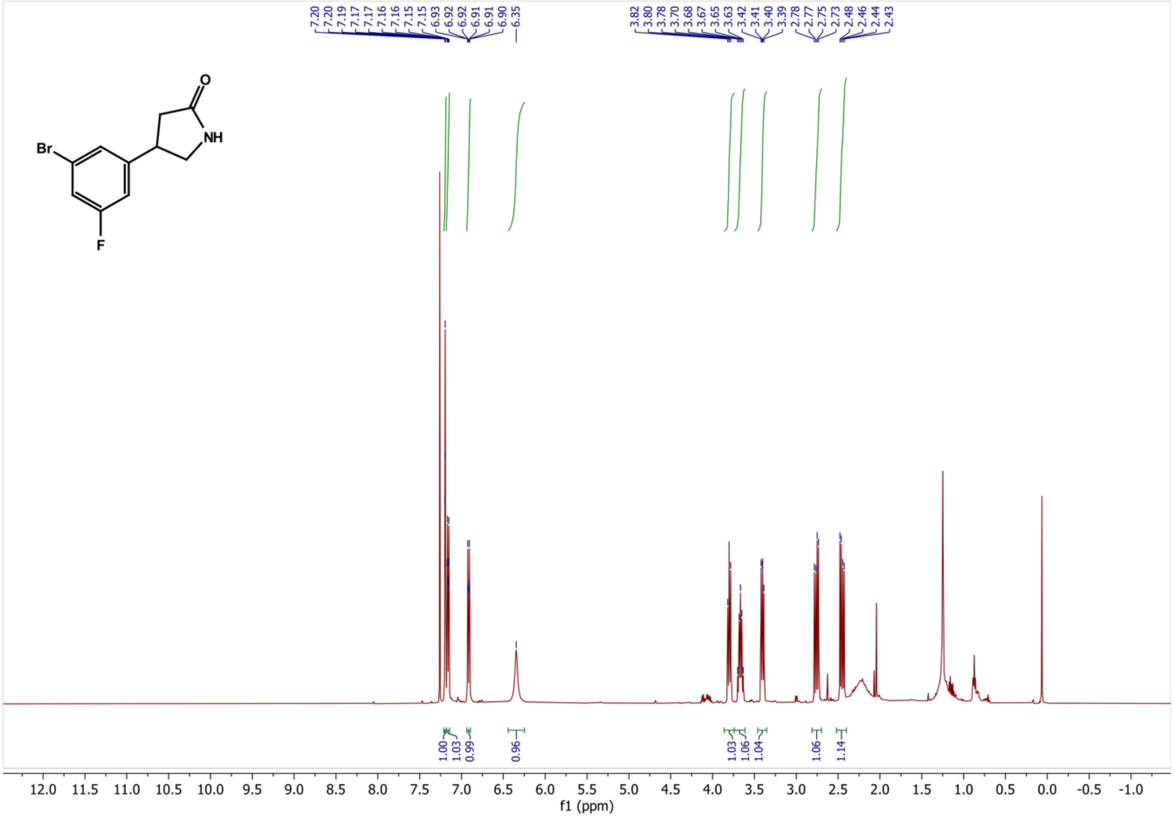

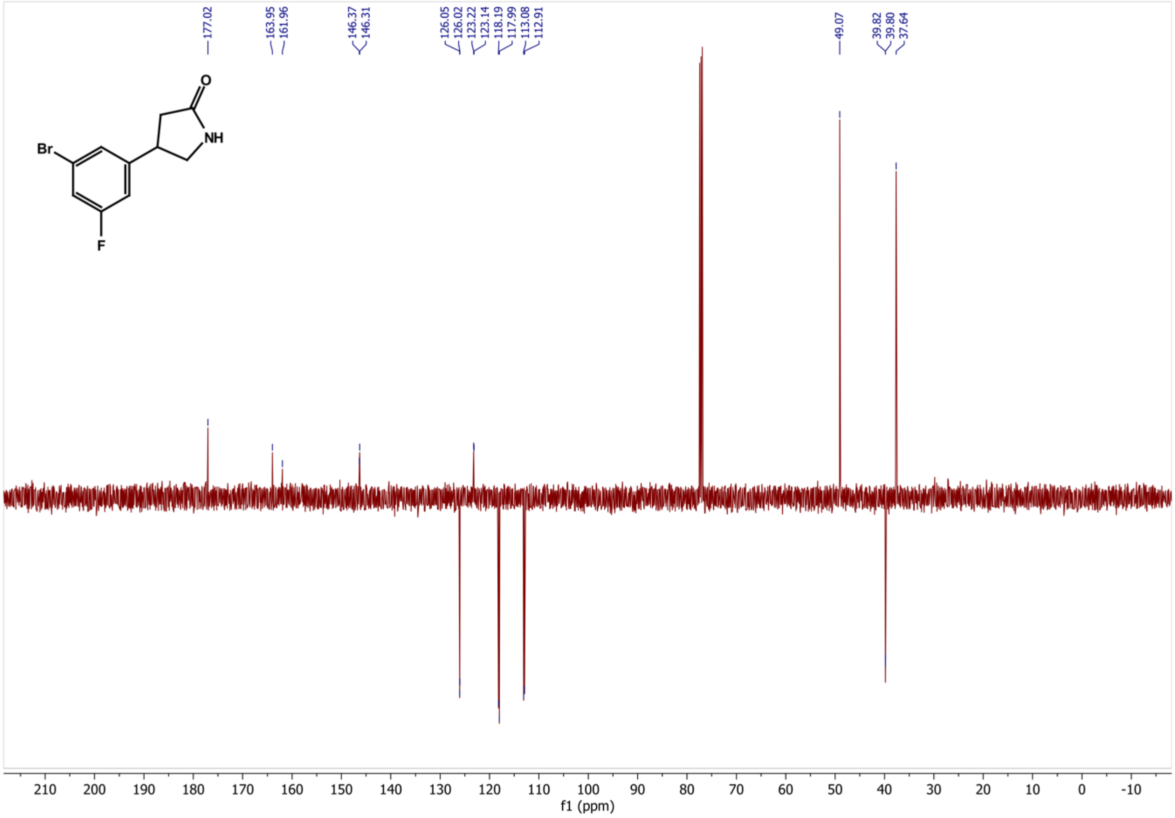

4-(3-bromo-5-fluorophenyl)-1-((3-methylpyridin-4-yl)methyl)-pyrrolidin-2-one

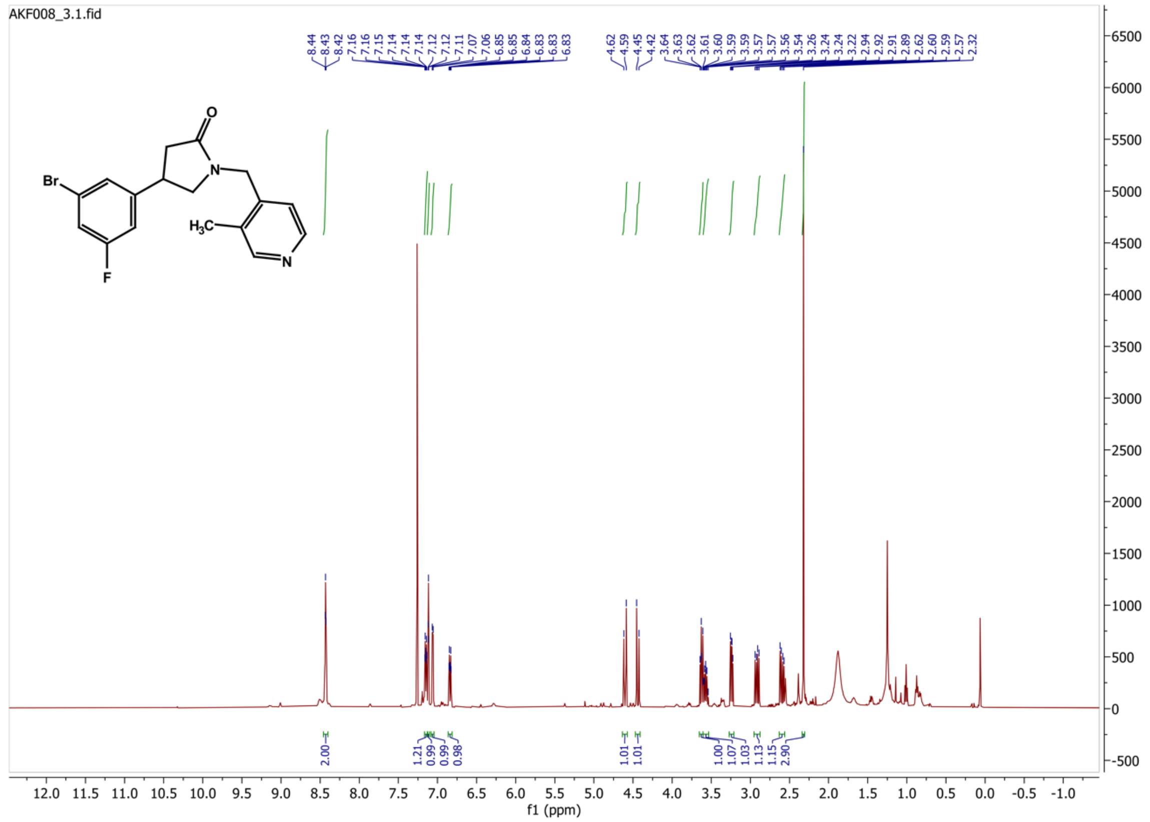

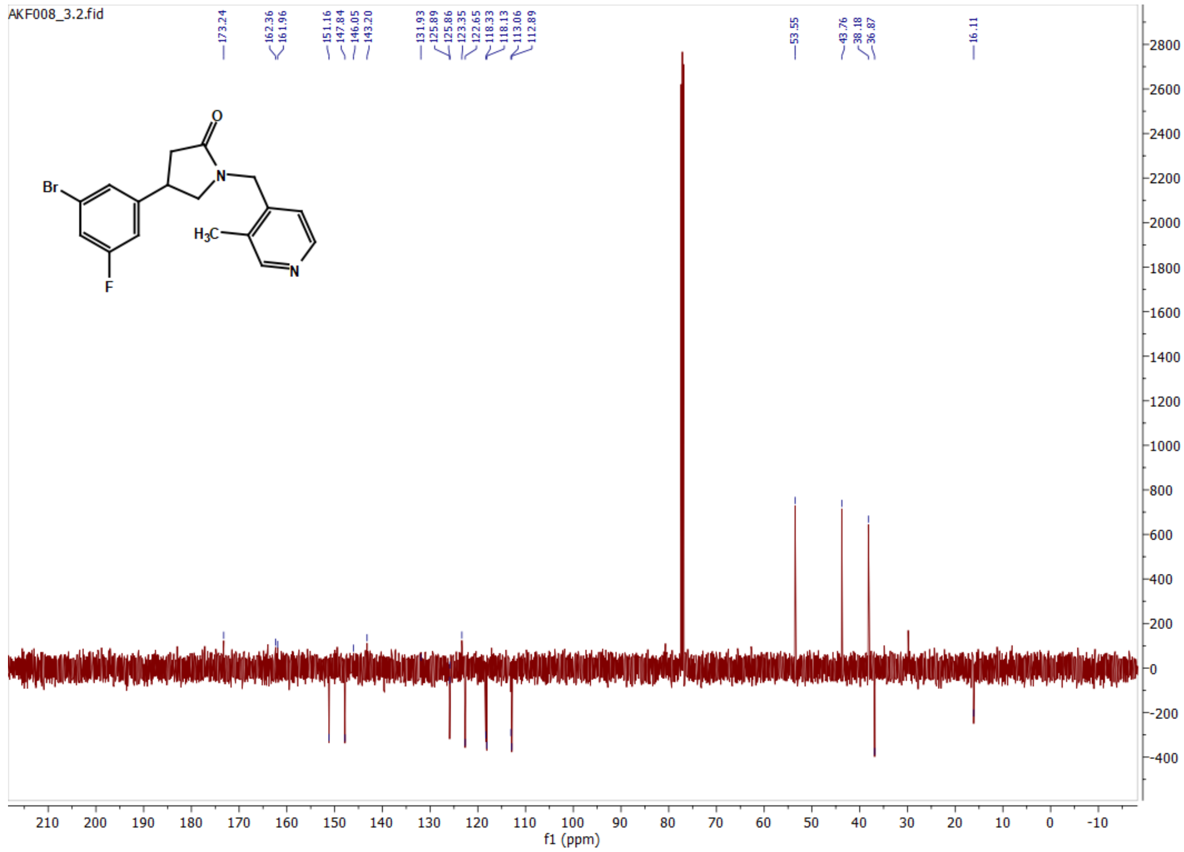

4-(3-fluoro-5-(trimethylstannyl)phenyl)-1-((3-methylpyridin-4-yl)methyl)pyrrolidin-2-one

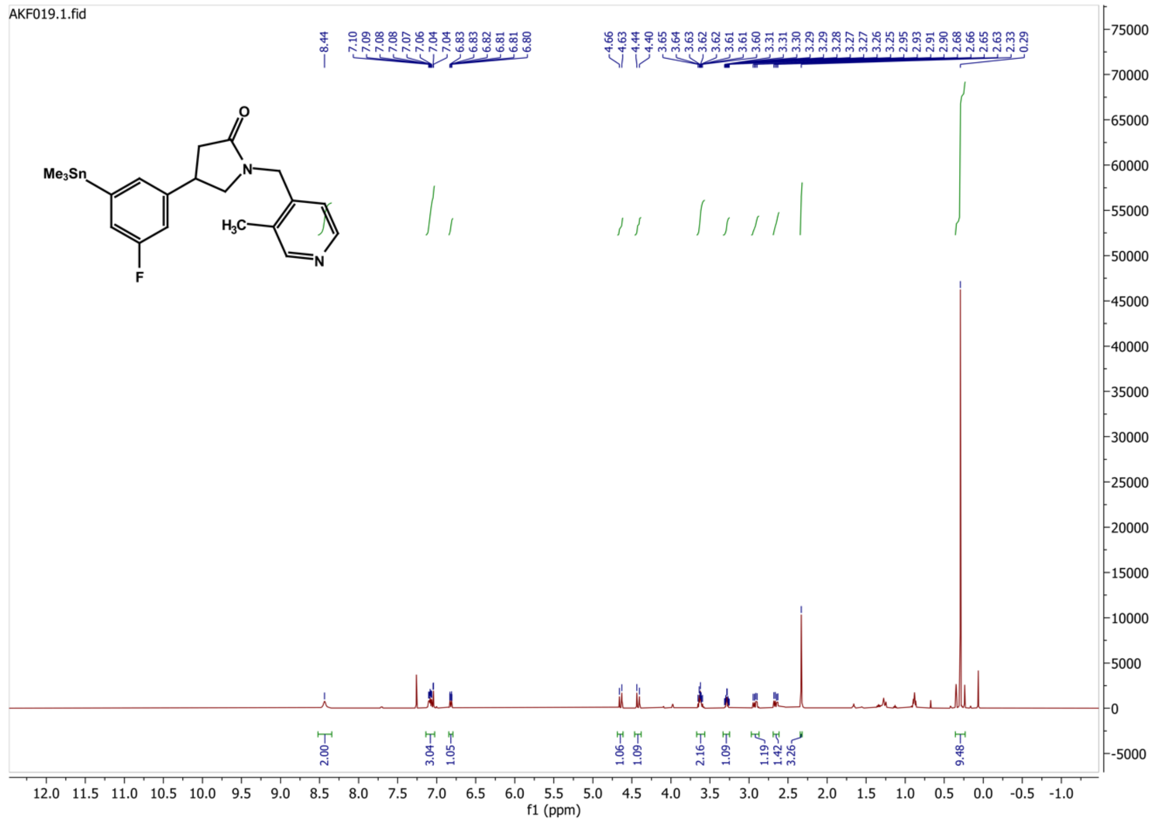

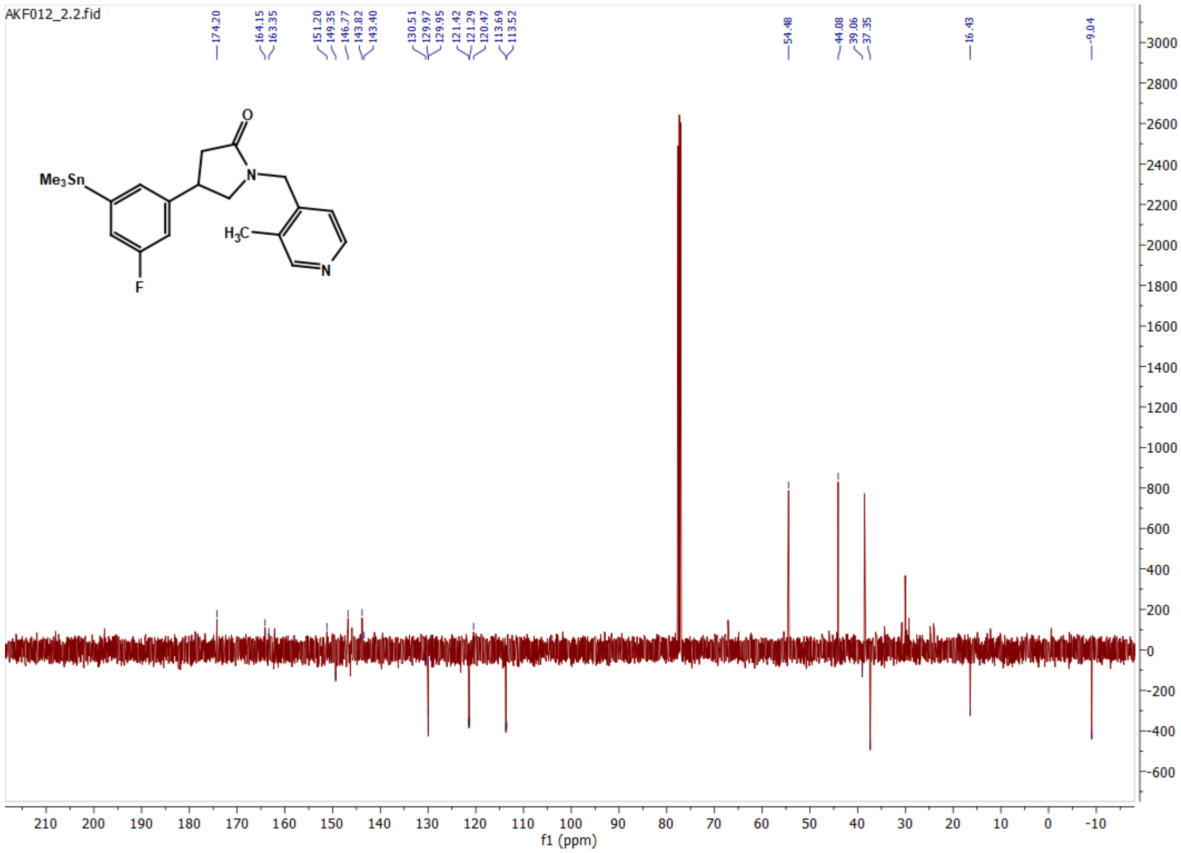

Ethyl (E)-3-(3,5-difluorophenyl)acrylate

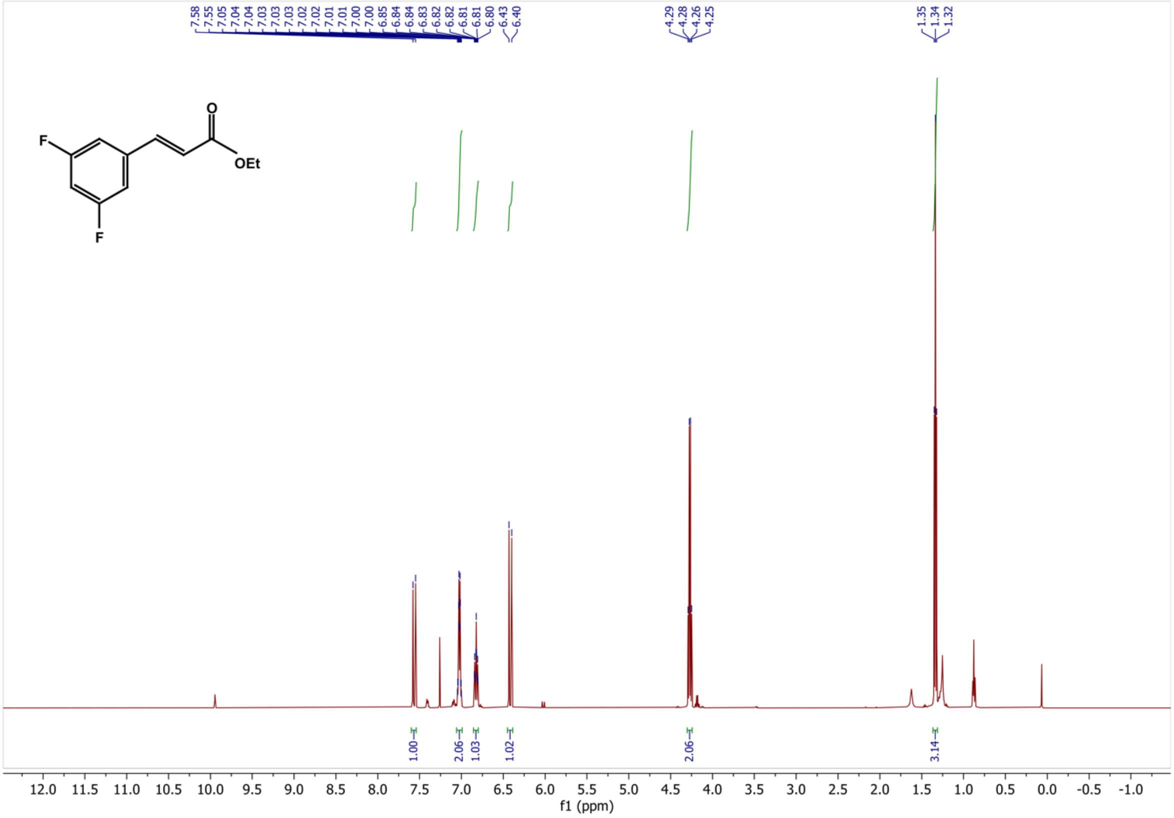

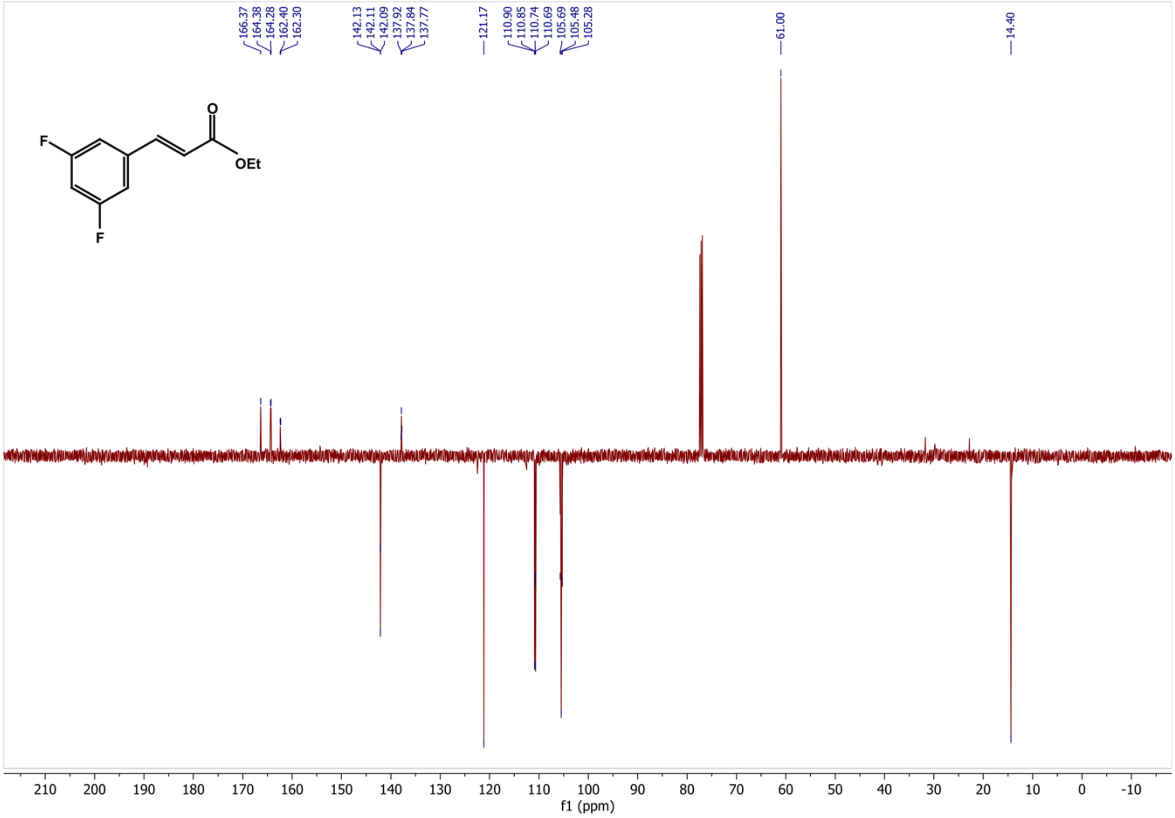

Ethyl 3-(3,5-difluorophenyl)-4-nitrobutanoate

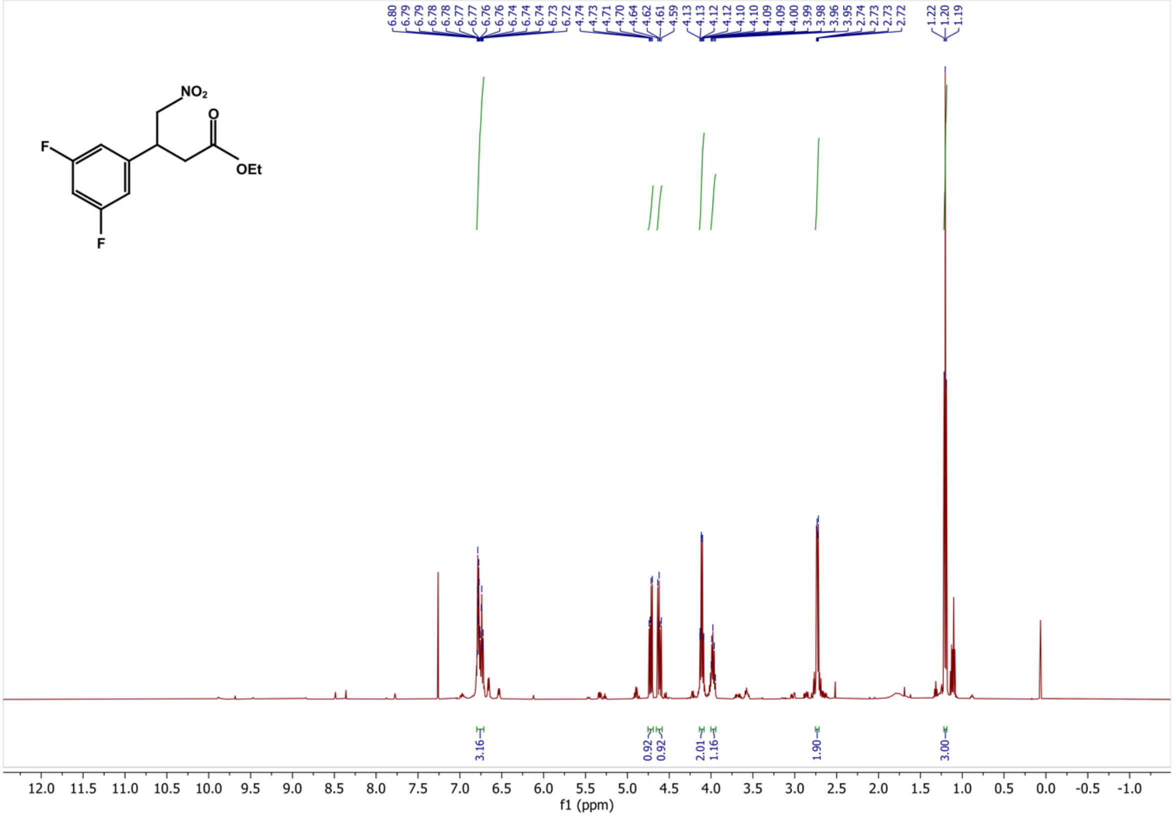

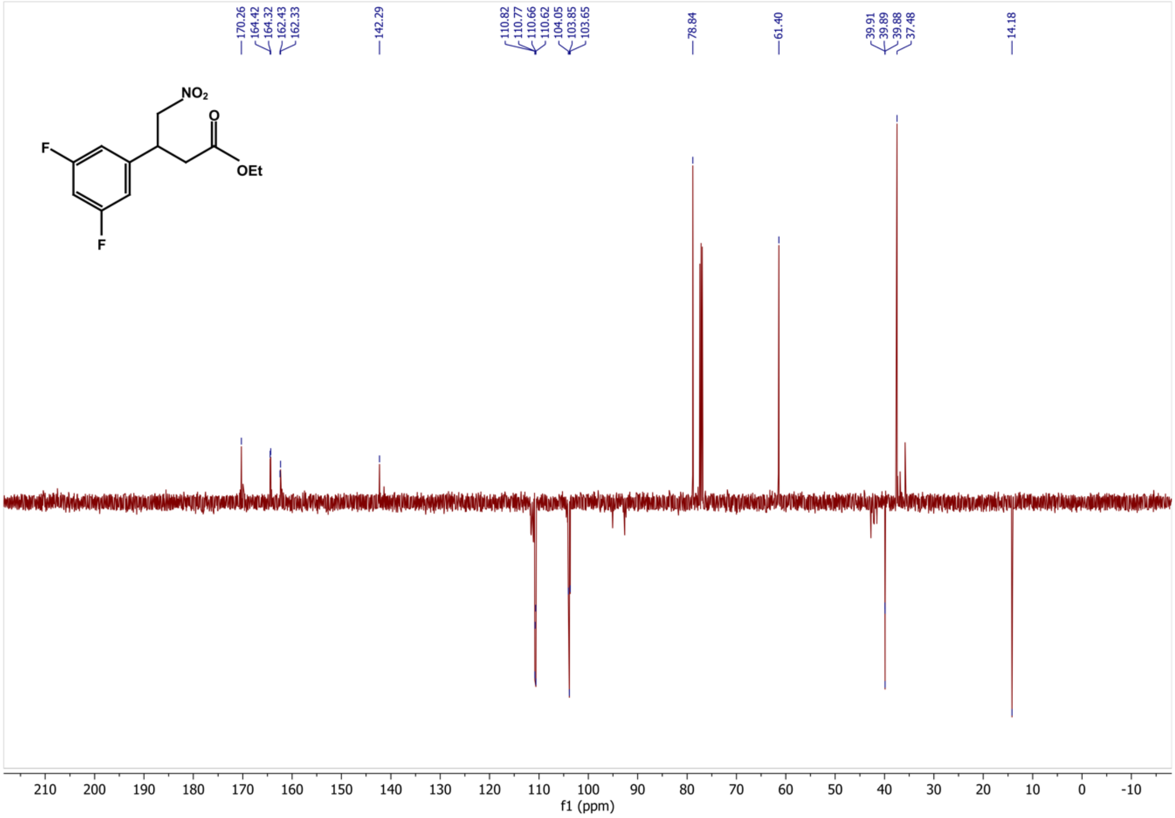

4-(3,5-difluorophenyl)pyrrolidin-2-one

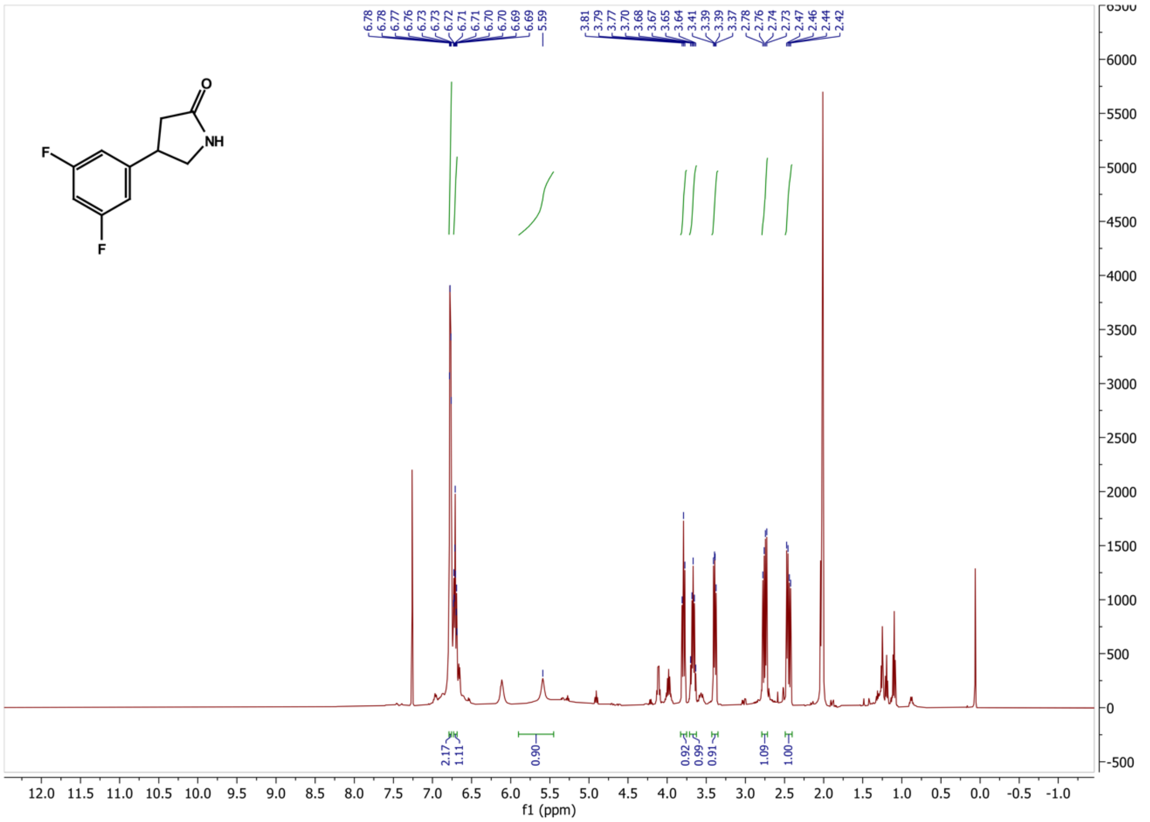

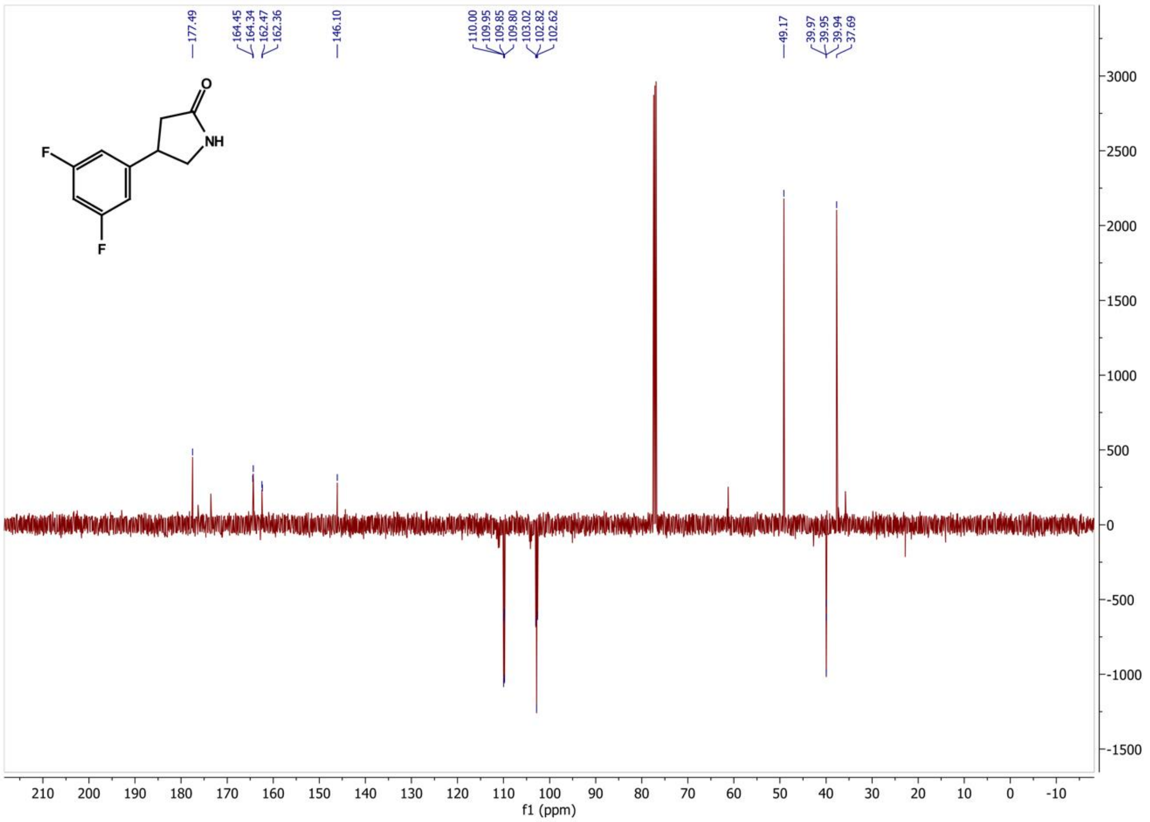

4-(3,5-difluorophenyl)-1-((3-methylpyridin-4-yl)methyl)-pyrrolidin-2-one

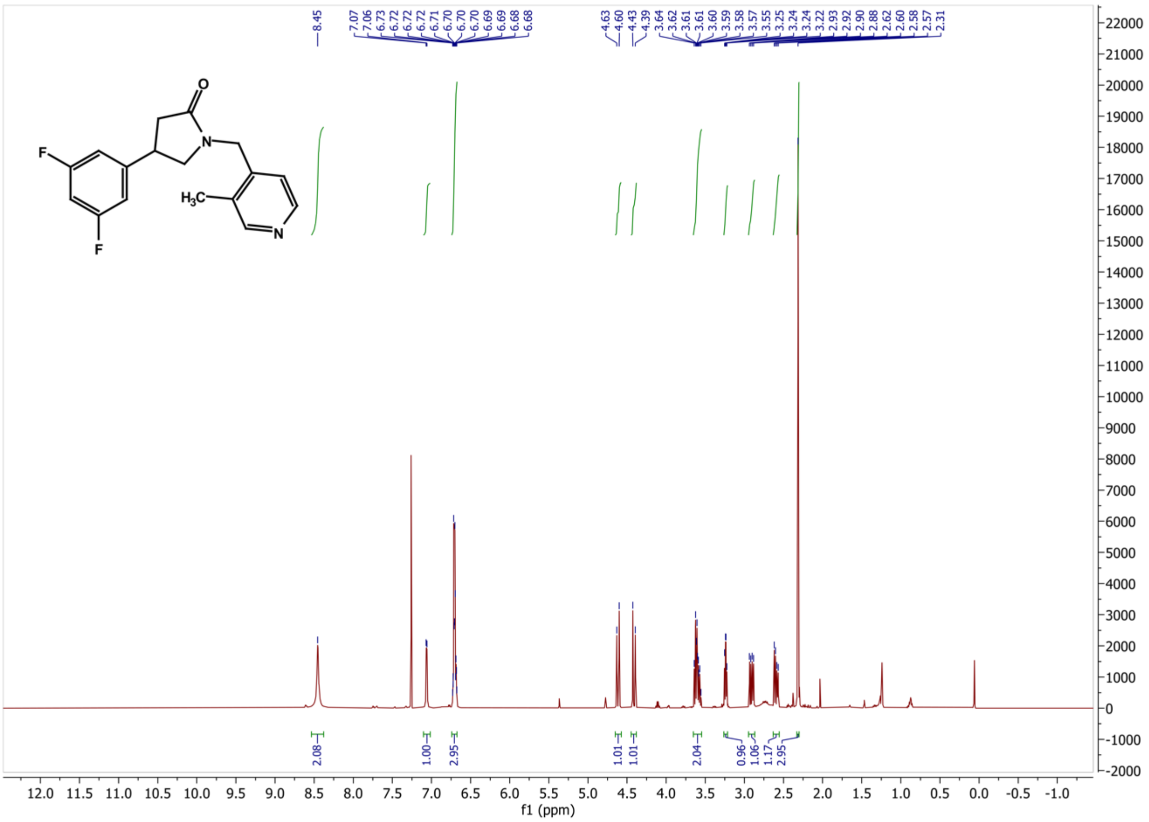

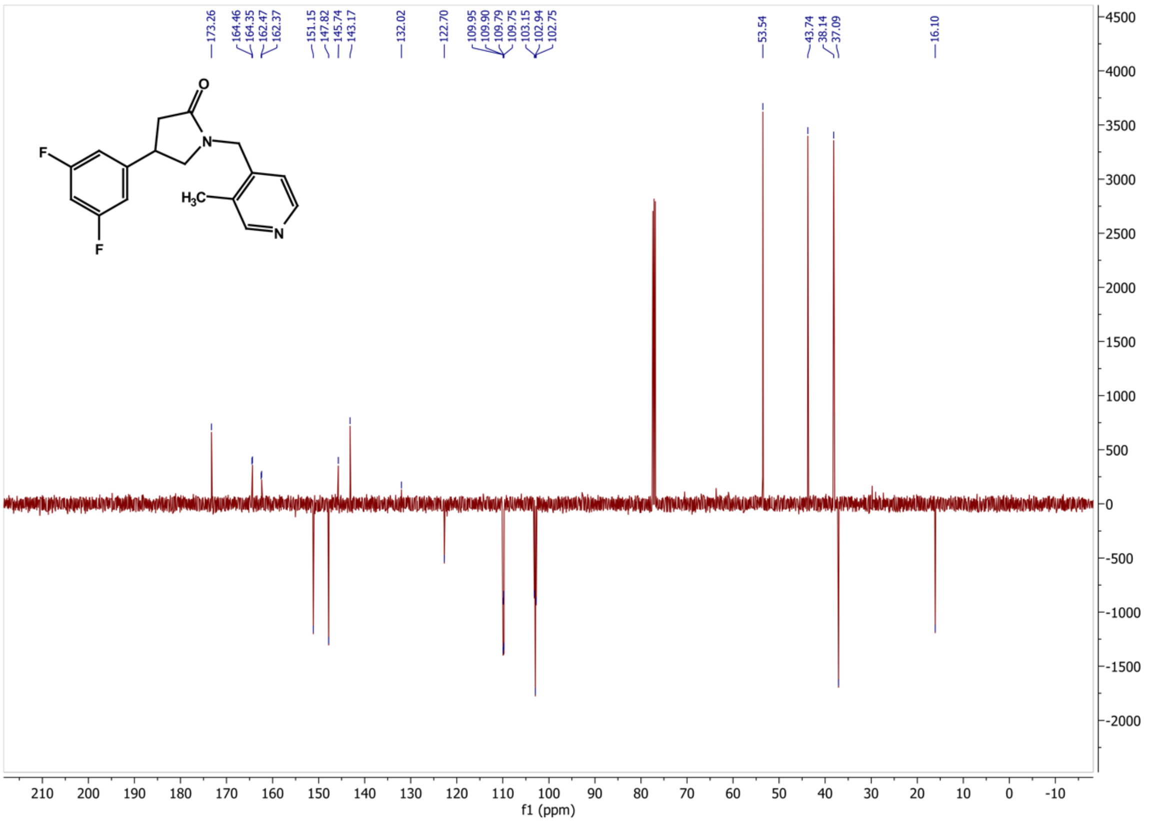

(1r,3r,5r,7r)-spiro[adamantane-2,2’-[1,3]dioxane]-4’,6’-dion-[3-((1,2,3,3-tetrakis(tert-butoxylcarbonyl)guanidino-3-iodonium)methyl)]ylide

Acquisition Time (sec)	3.2768	Comment	mFBG precursor co-evaporated with cyclohexane		Date	26 Feb 2019 10:57:20
Date Stamp	26 Feb 2019 10:57:20					
File Name	C:\Users\Alex\Dropbox\Work\VUmc\Contract research\[18F]MFBG as tracer (Bart de Keizer)\mFBG project\AP011 mFBG (2)\AP011 mFBG\3\fid					
Frequency (MHz)	500.20	Nucleus	1H	Number of Transients 16	Origin	spect
Original Points Count	32768	Owner	NMR	Points Count 32768	Pulse Sequence	zg30
Receiver Gain	20.20	SW(cyclical) (Hz)	10000.00	Solvent ACETONITRILE-d3	Spectrum Offset (Hz)	3069.2346
Spectrum Type	STANDARD	Sweep Width (Hz)	9999.70	Temperature (degree C) 25.160		

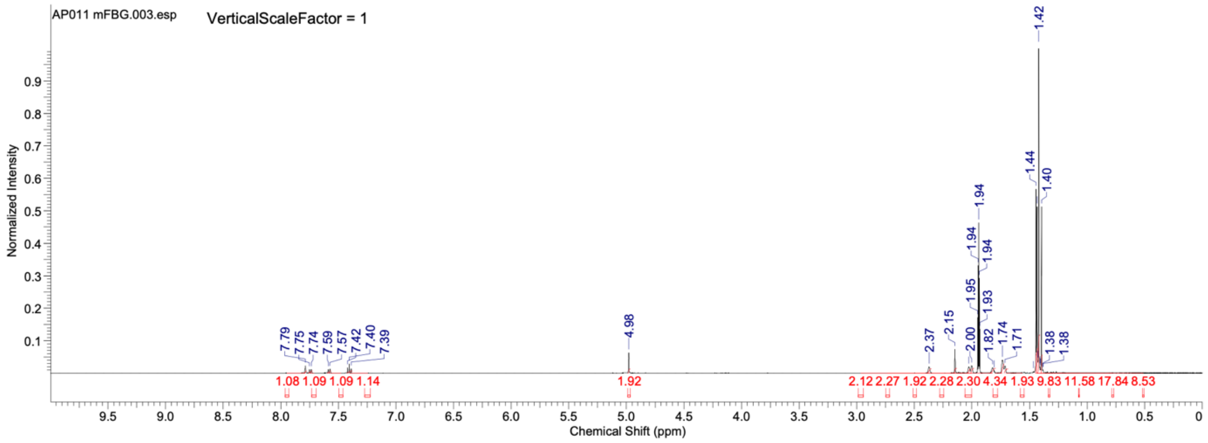

No.	(ppm)	(Hz)	Height
1	1.38	691.2	0.0098
2	1.38	692.1	0.0108
3	1.40	697.9	0.5130
4	1.40	702.1	0.0136
5	1.41	704.6	0.0209
6	1.42	710.4	1.0000
7	1.43	714.0	0.0386
8	1.43	717.7	0.5126
9	1.44	722.0	0.5665
10	1.47	733.6	0.0056
11	1.71	856.6	0.0224
12	1.74	868.2	0.0406
13	1.81	904.2	0.0076
14	1.82	910.6	0.0172
15	1.93	965.5	0.1364
16	1.94	967.9	0.2926
17	1.94	970.4	0.4630
18	1.94	972.8	0.3320
19	1.95	975.3	0.1723
20	2.00	1001.2	0.0235
21	2.03	1013.7	0.0201
22	2.15	1074.1	0.0738
23	2.37	1186.5	0.0202
24	4.98	2490.5	0.0631
25	7.39	3695.9	0.0144
26	7.40	3703.8	0.0295
27	7.42	3711.8	0.0177
28	7.57	3787.8	0.0129
29	7.59	3795.7	0.0112
30	7.74	3869.3	0.0121
31	7.74	3870.2	0.0108
32	7.75	3877.2	0.0113
33	7.78	3894.0	0.0141
34	7.79	3895.5	0.0233

Acquisition Time (sec)	1.1010	Comment	mFBG precursor co-evaporated with cyclohexane		Date	26 Feb 2019 11:25:04
Date Stamp	26 Feb 2019 11:25:04					
File Name	C:\Users\Alex\Dropbox\Work\VUmc\Contract research\[18F]MFBG as tracer (Bart de Keizer)\mFBG project\AP011 mFBG (2)\AP011 mFBG\4\fid					
Frequency (MHz)	125.78	Nucleus	13C	Number of Transients 512	Origin	spect
Original Points Count	32768	Owner	NMR	Points Count 32768	Pulse Sequence	jmod
Receiver Gain	2050.00	SW(cyclical) (Hz)	29761.90	Solvent ACETONITRILE-d3	Spectrum Offset (Hz)	12703.8379
Spectrum Type	APT	Sweep Width (Hz)	29761.00	Temperature (degree C) 25.160		

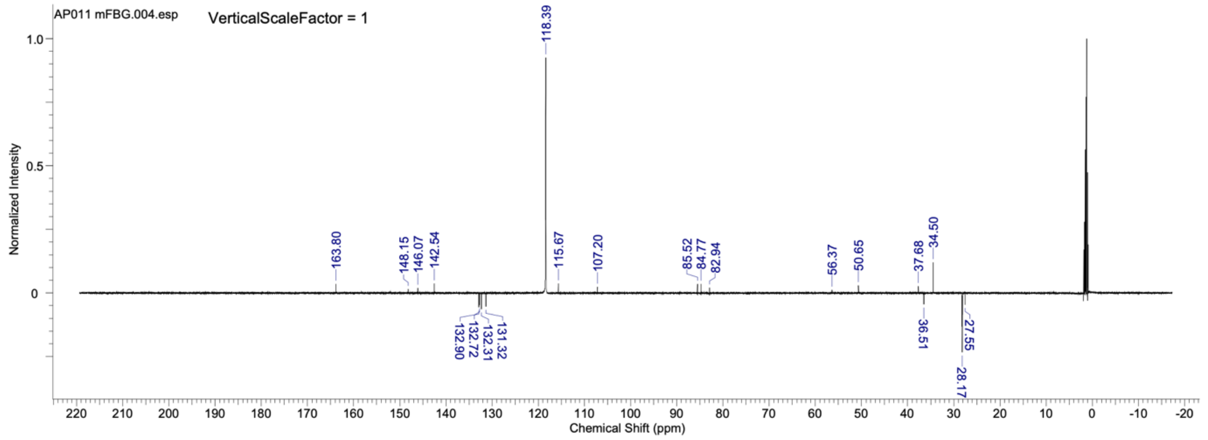

No.	(ppm)	(Hz)	Height
1	27.55	3464.5	-0.0466
2	28.14	3539.0	-0.0852
3	28.17	3542.7	-0.2335
4	28.20	3546.3	-0.1328
5	34.50	4339.2	0.1195
6	36.51	4591.7	-0.0454
7	37.68	4739.7	0.0269
8	50.65	6370.1	0.0301
9	56.37	7090.3	0.0122
10	82.94	10431.8	0.0200
11	84.77	10662.5	0.0354
12	85.52	10756.1	0.0349
13	107.20	13483.6	0.0236
14	115.67	14548.1	0.0366
15	118.39	14890.5	0.9260
16	131.32	16517.2	-0.0538
17	132.31	16641.6	-0.0640
18	132.72	16692.5	-0.0482
19	132.90	16716.1	-0.0560
20	142.54	17927.7	0.0368
21	146.07	18371.8	0.0194
22	148.15	18633.4	0.0145
23	163.80	20601.6	0.0351

Meta-fluorobenzylguanidine

Acquisition Time (sec)	3.2768	Date	15 Feb 2019 14:52:00			Date Stamp	15 Feb 2019 14:52:00
File Name	C:\Users\Alex\Dropbox\Work\VUmc\Contract research\[18F]MFBG as tracer (Bart de Keizer)\mFBG project\AP-006 mfbg ref\AP-004 mfbg ref\1\fid						
Frequency (MHz)	500.20	Nucleus	1H	Number of Transients	16	Origin	spect
Original Points Count	32768	Owner	NMR	Points Count	32768	Pulse Sequence	zg30
Receiver Gain	16.00	SW(cyclical) (Hz)	10000.00	Solvent	DEUTERIUM OXIDE		
Spectrum Offset (Hz)	3108.8296	Spectrum Type	STANDARD	Sweep Width (Hz)	9999.70	Temperature (degree C)	25.160

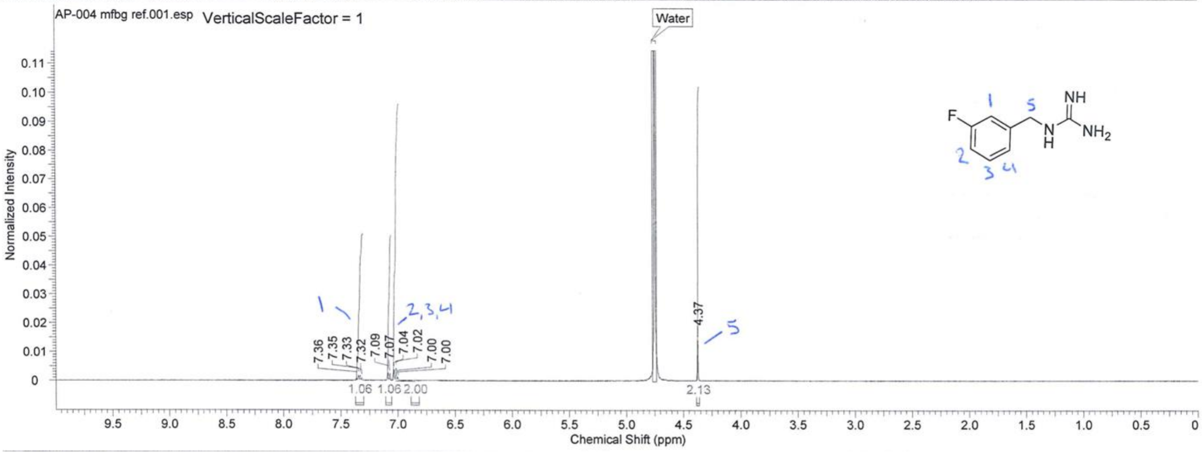

Acquisition Time (sec)	1.1010	Date	15 Feb 2019 15:21:52			Date Stamp	15 Feb 2019 15:21:52
File Name	C:\Users\Alex\Dropbox\Work\VUmc\Contract research\[18F]MFBG as tracer (Bart de Keizer)\mFBG project\AP-006 mfbg ref\AP-004 mfbg ref\2\fid						
Frequency (MHz)	125.78	Nucleus	13C	Number of Transients	512	Origin	spect
Original Points Count	32768	Owner	NMR	Points Count	32768	Pulse Sequence	jmod
Receiver Gain	2050.00	SW(cyclical) (Hz)	29761.90	Solvent	DEUTERIUM OXIDE		
Spectrum Offset (Hz)	12577.5391	Spectrum Type	APT	Sweep Width (Hz)	29761.00	Temperature (degree C)	25.160

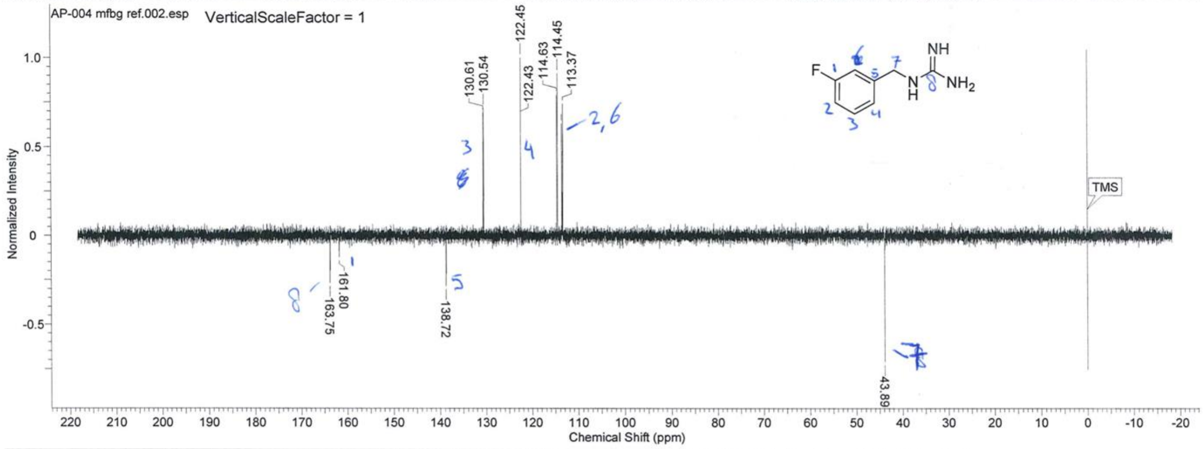

No.	(ppm)	Annotation	Layer No.	Created By	Created At	Modified By	Modified At
1	[0.03 .. 0.05]	TMS	1	alex	Wed 2/20/2019 8:43:15 AM		

No.	(ppm)	(Hz)	Height	No.	(ppm)	(Hz)	Height	No.	(ppm)	(Hz)	Height	No.	(ppm)	(Hz)	Height
1	43.89	5519.9	-0.7102	4	114.45	14395.4	0.9124	7	122.45	15400.9	1.0000	10	138.72	17448.1	-0.2846
2	113.37	14259.2	0.7415	5	114.63	14417.2	0.7949	8	130.54	16418.1	0.7177	11	161.80	20350.9	-0.1227
3	113.54	14281.0	0.6306	6	122.43	15398.1	0.6625	9	130.61	16427.2	0.6921	12	163.75	20595.2	-0.2669

5-(3-Cyano-5-(pyridin-2-ylethynyl)phenyl)-2,4-dimethoxy-8-methyldibenzo[b,d]thiophen-5-ium trifluoromethanesulfonate

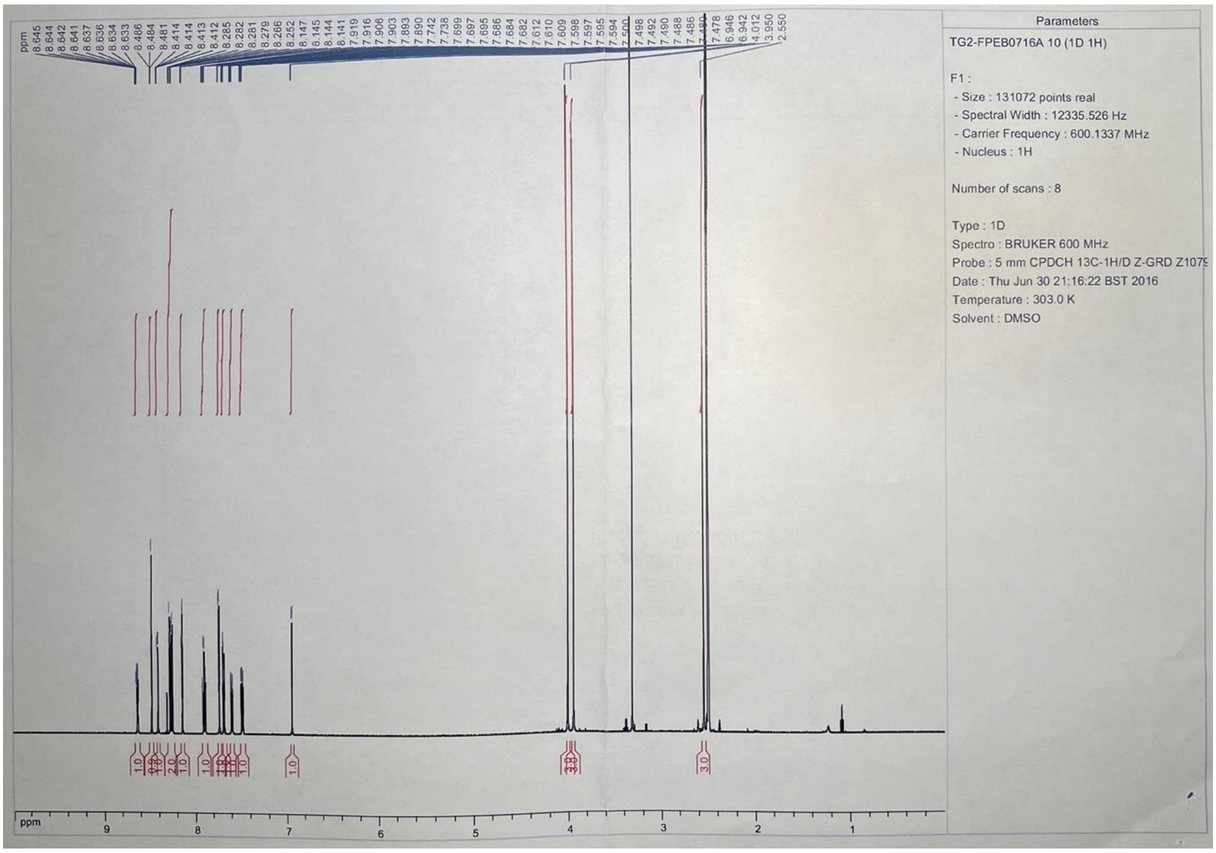

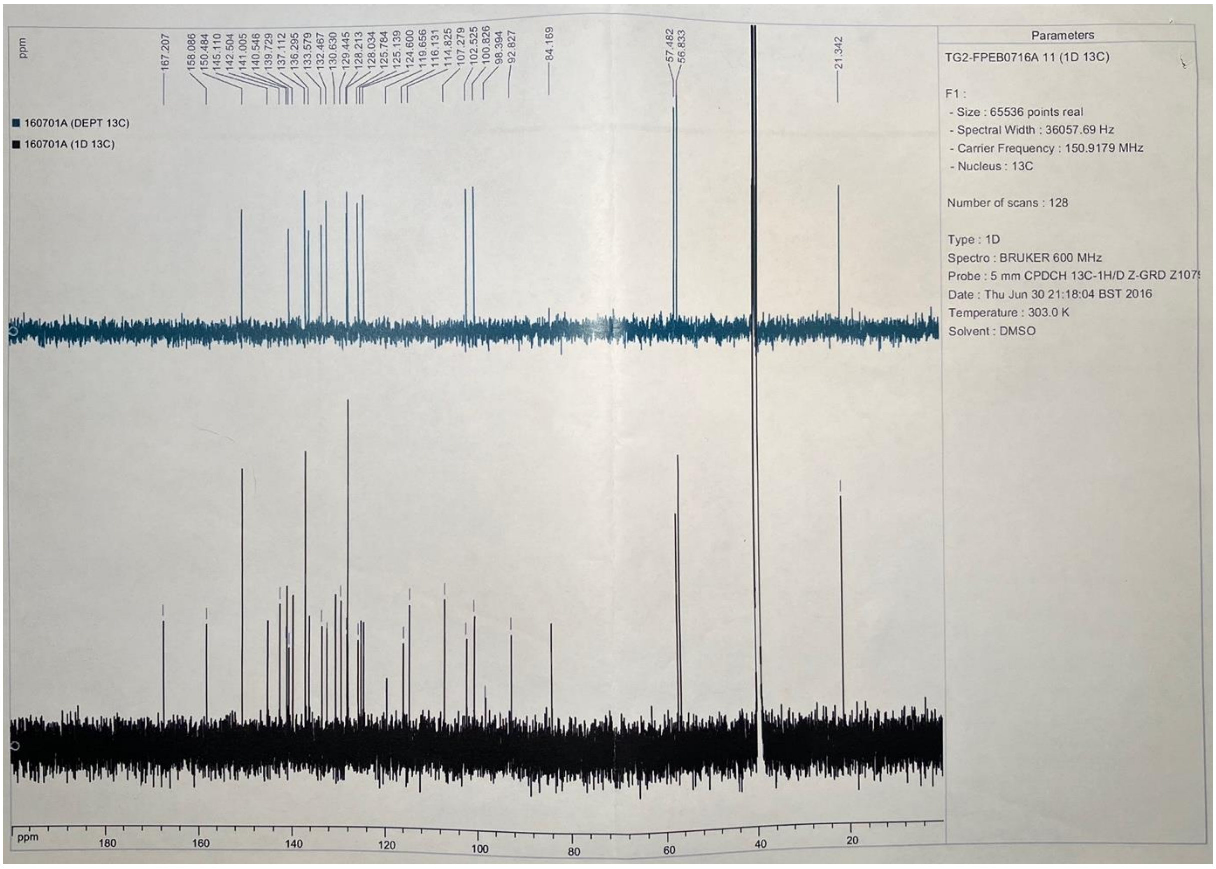

3-fluoro-5-(pyridin-2-ylethynyl)benzonitrile

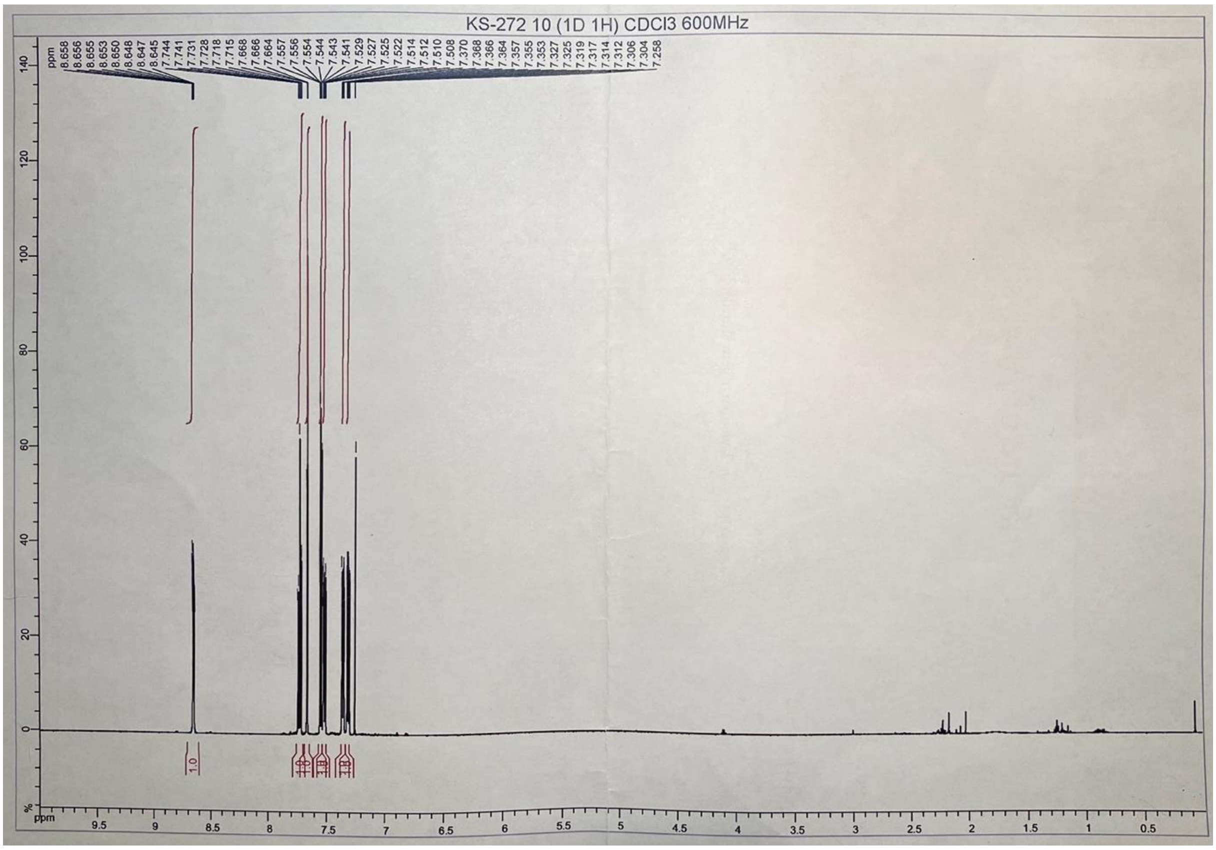

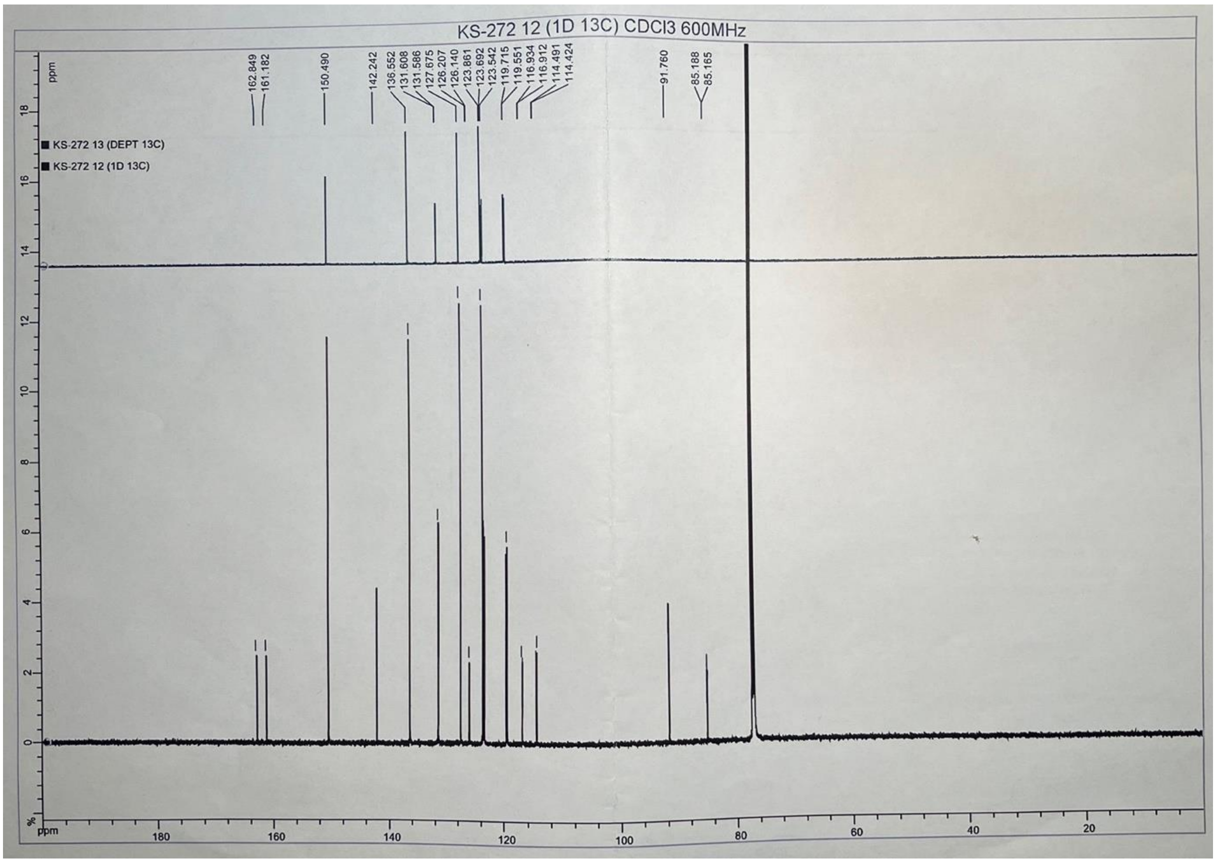

6. References

[1] H. H. Coenen, A. D. Gee, M. Adam, G. Antoni, C. S. Cutler, Y. Fujibayashi, J. M. Jeong, R. H. Mach, T. L. Mindt, V. W. Pike, A. D. Windhorst, *Nucl. Med. Biol.* **2017**, *55*, v–xi.

[2] M. M. Herth, S. Ametamey, D. Antuganov, A. Bauman, M. Berndt, A. F. Brooks, G. Bormans, Y. S. Choe, N. Gillings, U. O. Häfeli, M. L. James, K. Kopka, V. Kramer, R. Krasikova, J. Madsen, L. Mu, B. Neumaier, M. Piel, F. Rösch, T. Ross, R. Schibli, P. J. H. Scott, V. Shalgunov, N. Vasdev, W. Wadsak, B. M. Zeglis, *Nucl. Med. Biol.* **2021**, *93*, 19–21.

[3] S. Li, Z. Cai, X. Wu, D. Holden, R. Pracitto, M. Kapinos, H. Gao, D. Labaree, N. Nabulsi, R. E. Carson, Y. Huang, *ACS Chem. Neurosci.* **2019**, *10*, 1544–1554.
